# Bromination Functionalization of Diazo Compounds with CBr_4_ via Convergent Paired Electrolysis

**DOI:** 10.1021/acs.orglett.5c05051

**Published:** 2026-01-13

**Authors:** Qian Wang, Wentian Wu, Haibo Mei, Jorge Escorihuela, Romana Pajkert, Gerd-Volker Röschenthaler, Jianlin Han

**Affiliations:** † Jiangsu Co-Innovation Center of Efficient Processing and Utilization of Forest Resources, College of Chemical Engineering, 74584 Nanjing Forestry University, Nanjing 210037, China; ‡ Department of Organic Chemistry, Faculty of Pharmacy and Food Sciences, 16781 Universitat de València, Avda. Vicente Andrés Estellés s/n, Burjassot, 46100 Valencia, Spain; § School of Science, 84498 Constructor University Bremen gGmbH, Campus Ring 1, 28759 Bremen, Germany

## Abstract

An electrochemical bromination of diazo compounds with CBr_4_ as a bromine source via convergent paired electrolysis has been developed, which affords α-bromo phosphonates as products in up to 96% yields. This work represents the first example of direct anodic oxidation of diazos to couple with an *in situ*-generated nucleophile from the cationic reduction of CBr_4_ by using CH_2_Cl_2_ as a hydrogen source. This reaction features mild conditions, good substrate compatibility, and scale-up applicability, which represents a new electrochemical reaction mode of diazo compounds and also provides easy access to α-bromo phosphonates.

## Graphical abstract

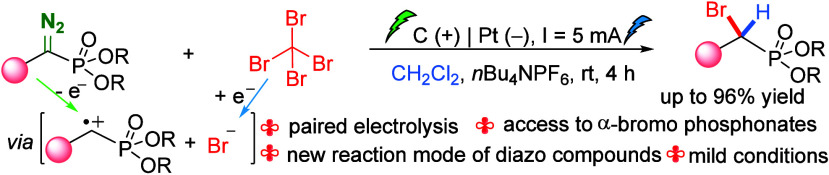

Diazo compounds belong to an extremely important type of organic building blocks, which are found in many natural products^1^ and bioactive molecules.^2^ Diazo compounds feature privileged functional units and can act as powerful and versatile reagents with diverse reactivity patterns.^3^ In recent decades, diazo compounds have found extensive applications in organic synthesis, polymer synthesis, medicinal chemistry, material science, and various other fields. As a result, continuous efforts have been devoted to the development of transformations involving these organic compounds in past decades.^3–5^

Among various transformations employing diazo compounds, electrochemical multicomponent reactions of diazo compounds have attracted increasing attention in recent years, which is due to the elimination of the need for external oxidants or reductants and the efficiency of generating higher molecular complexity under mild and sustainable conditions.^6^ In 2022, the Huang group developed an electrochemical carbenoid insertion reaction of diazo compounds with thiols and salicylic acids. The reaction proceeded via anodic oxidation of thiols into sulfur radicals, followed by coupling with free carbene from α-diazoester to afford the corresponding products.^7^ In 2023, the Lei group developed an interesting electrochemical difunctionalization of diazo compounds with two different nucleophiles via the anodic oxidation of thiols or *N*-methyl anilines to generate radical intermediates.^8^ Later in 2024, an electrochemical selenidation-triggered difunctionalization of diazo compounds with varieties of nucleophiles was reported, which proceeded via anodic oxidation of diselenides, affording a diverse array of selenium-containing pyrazole esters and alkoxy esters as products.^9^ The electrochemical cycloaddition reaction between alkenes and diazo esters was also developed, which proceeded via anodic oxidation of olefins followed by a [2 + 1] cycloaddition with diazo compounds toward cyclopropane synthesis.^10^ Although some elegant works have been developed, these reactions focus on diazo compounds acting as radical acceptors (Scheme 1a).^11^ To the best of our knowledge, no electrochemical reaction via direct anodic oxidation of diazo compounds has been explored until now.

**Scheme 1:**
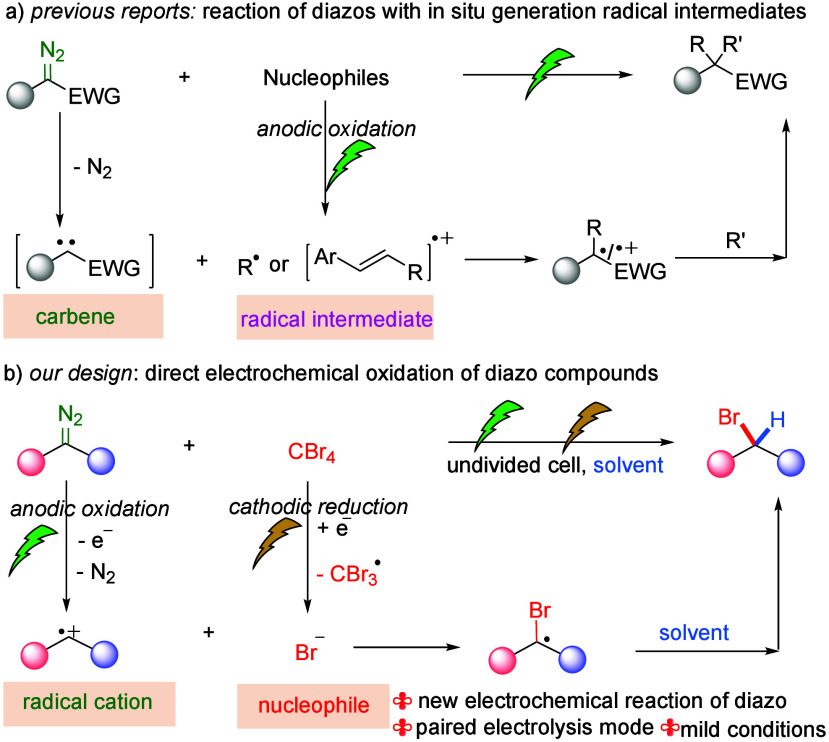
Electrochemical Reactions of Diazo Compounds

In view of the above limitations^7–11^ and in continuation of our interest in electrochemical transformations^12^ and diazo compound functionalization,^13^ we envision that electrochemical radical functionalization of diazo compounds via direct anodic oxidation of diazo compounds should be feasible by using suitable nucleophiles. Herein, we report an electrochemical bromination reaction of diazo compounds via convergent paired electrolysis with CBr_4_ as a bromine source and CH_2_Cl_2_ as a hydrogen source (Scheme 1b). The diazo compounds undergo anodic oxidation to generate carbon radical cations. Meanwhile, CBr_4_ is reduced at the cathode to produce the final product. This work is the first example of the direct anodic oxidation of diazo compounds to couple with the nucleophile. This paired electrolysis mode differs from the previous electrochemical reactions of diazo compounds, which involve only a single electrode in the reaction. Moreover, this reaction provides a new and efficient method for the synthesis of α-bromo phosphonates.

To test this hypothesis, there are two problems that should be taken into consideration. One is the high oxidative potential of diazo compounds, which makes the anodic oxidation difficult. Thus, the nucleophile may preferentially oxidize at the anode to form a radical species. The second challenge is whether the nucleophilic precursors could couple with the *in situ*-generated radical cation intermediate from diazo compounds in this electrosynthesis. Our proof-of-concept work commenced by testing the reaction of diethyl diazo­(phenyl)­methyl phosphonate (**1a**) with tetrabromomethane (CBr_4_, **2**) as a bromine source under electrochemical conditions (Table 1). Extensive optimization revealed that the reaction of **1a** with CBr_4_ could occur to generate desired α-bromo phosphonate **3a** in 58% yield when it was conducted in dichloromethane with *n*Bu_4_NBF_4_ as an electrolyte in an undivided cell equipped with a graphite anode and a graphite cathode under a constant current of 5 mA for 4 h at room temperature (entry 1). Then, several common organic solvents were screened for this electrochemical transformation. Acetonitrile and 1,2-dichloroethane were also suitable solvents for this reaction, achieving desired product **3a** in 46 and 57% yields, respectively (entries 2 and 3). However, no desired product **3a** was detected when the reaction used protic solvent MeOH as the reaction medium (entry 4). This is mainly due to the dual roles of the solvent, reaction media, and hydrogen atom source. Other electrolytes, including *n*Bu_4_NI, *n*Bu_4_NPF_6_, *n*Bu_4_NAc, and *n*Bu_4_NClO_4_, were also used for this reaction (entries 5–8). *n*Bu_4_NPF_6_ was demonstrated to be the most effective one, which afforded a slightly increased yield (60%, entry 6). No desired product **3a** was detected when the reaction was conducted by using *n*Bu_4_NI or *n*Bu_4_NAc as the electrolyte (entries 5 and 7). It was found that other electrode combinations, such as (+)­C|Pt(−), (+)­Pt|Pt(−), and (+)­Pt|C(−), also worked well to furnish corresponding product **3a**, and the best yield was observed using (+)­C|Pt(−) as electrodes (65% yield, entry 9). Finally, variations in the loading amount of diazo compound **1a** and current density were carried out (entries 12–15). Fortunately, the yield could be further increased to 96% when 3.0 equiv of diazo **1a** was used (entry 13). The current density is crucial for this transformation, and decreased yields were observed under a decreased or increased current density (entries 14 and 15).

**Table 1:**
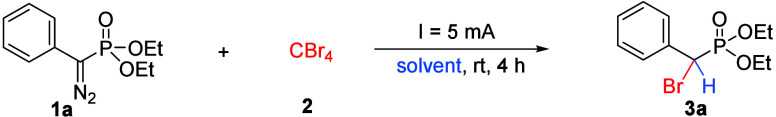
Optimization of Reaction Conditions^a^

entry	electrolyte	solvent	electrode	yield (%)^b^
1	*n*Bu_4_NBF_4_	CH_2_Cl_2_	C/C	58
2	*n*Bu_4_NBF_4_	MeCN	C/C	46
3	*n*Bu_4_NBF_4_	ClCH_2_CH_2_Cl	C/C	57
4	*n*Bu_4_NBF_4_	MeOH	C/C	n.d.
5	*n*Bu_4_NI	CH_2_Cl_2_	C/C	n.d.
6	*n*Bu_4_NPF_6_	CH_2_Cl_2_	C/C	60
7	*n*Bu_4_NA_C_	CH_2_Cl_2_	C/C	n.d.
8	*n*Bu_4_NClO_4_	CH_2_Cl_2_	C/C	25
9	*n*Bu_4_NPF_6_	CH_2_Cl_2_	C/Pt	65
10	*n*Bu_4_NPF_6_	CH_2_Cl_2_	Pt/Pt	57
11	*n*Bu_4_NPF_6_	CH_2_Cl_2_	Pt/C	64
12	*n*Bu_4_NPF_6_	CH_2_Cl_2_	C/Pt	36^c^
13	*n*Bu_4_NPF_6_	CH_2_Cl_2_	C/Pt	96^d^
14	*n*Bu_4_NPF_6_	CH_2_Cl_2_	C/Pt	51^d^ ^,^ ^e^
16	*n*Bu_4_NPF_6_	CH_2_Cl_2_	C/Pt	58^d^ ^,^ ^f^

a Reaction conditions: diazo **1a** (0.2 mmol, 2.0 equiv), CBr_4_ **2** (0.1 mmol), electrolyte (0.7 mmol), solvent (12 mL), I = 5 mA, at room temperature for 4 h.

b Isolated yield based on **2**.

c **1a** (1.0 equiv).

d **1a** (3.0 equiv).

e I = 2.5 mA.

f I = 7.5 mA.

After establishing the optimized reaction conditions, we then examined the substrate scope of diazo compounds **1** for this electrochemical reaction to access structurally diverse α-bromo phosphonates (Scheme 2). Generally, all of the employed aryl diazos bearing substituents at different positions of the phenyl ring, including alkyl, halo, and aryl, were all tolerated in this electrochemical bromination reaction, providing desired α-bromo phosphonates **3** in good to excellent chemical yields. Notably, the substrate with electron-withdrawing properties (CF_3_, **1m**) worked well, leading to corresponding product **3m** in 91% yield. However, no desired product was detected for the substrate with a strong electron-donating group (OMe, **1k**). The reaction did not show an obvious steric hindrance effect. For example, *ortho*-, *meta*-, and *para*-methyl-substituted phenyl diazo substrates were all well engaged in the reaction, generating corresponding products **3b**–**3d** in 73–74% yields. Subsequently, phenyl diazo substrates bearing functional groups were examined in this electrochemical reaction. We were delighted to find that phenyl diazos bearing a cyano group (**1n**) and an ester group (**1p**) were also compatible with this transformation, affording desired products **3n** and **3p** in 87 and 70% yields, respectively. It is worth mentioning that naphthyl-substituted diazo was also a viable substrate in this reaction, affording corresponding product **3q** in 69% yield. Finally, various phosphonate groups on the diazo substrates were examined. Pleasingly, substrates containing different esters, including methyl, isopropyl, and butyl, also displayed good reactivity to provide products **3r**–**3t** in 57–86% yields. The length of the alkyl chain shows an effect on the reaction outcome, and a 57% yield was obtained for the case with the butyl phosphonate group (**3t**). To demonstrate the robust nature of the protocol, we performed the reaction on a 2.0 mmol scale, and desired product **3a** was obtained in 81% yield, underlining the promising potential of this electrochemical process to construct functionalized α-bromo phosphonates.

**Scheme 2:**
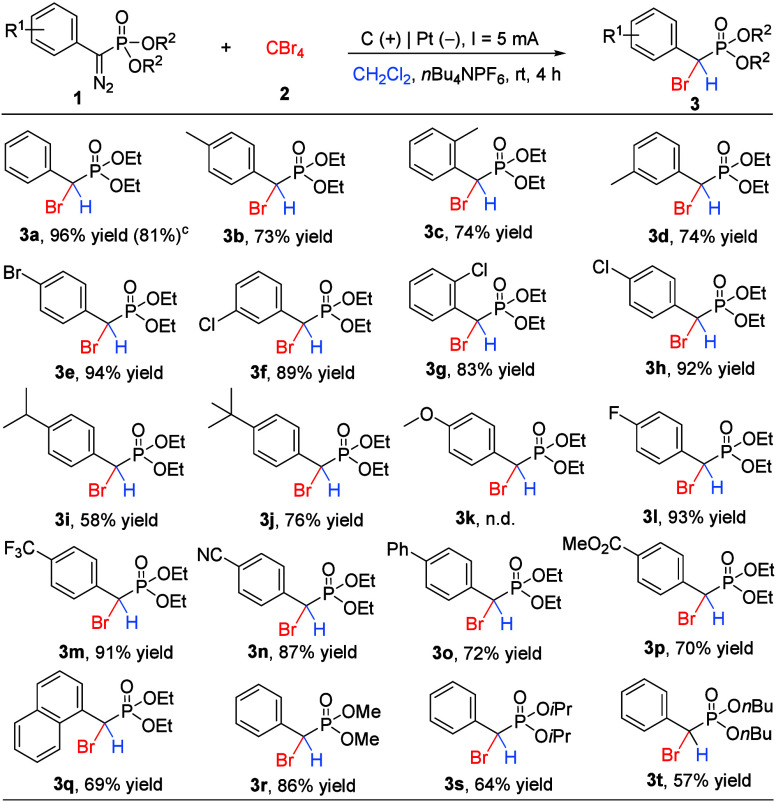
Substrate Scope of Diazo^a^ ^,^ ^b^

a Reactions conditions: diazo **1** (0.3 mmol), CBr_4_ **2** (0.1 mmol), *n*Bu_4_NPF_6_ (0.7 mmol), CH_2_Cl_2_ (12 mL), a carbon anode, a platinum cathode, and a constant current of 5 mA for 4 h at room temperature.
b Isolated yield based on **2**.
c Scale-up synthesis with 2.0 mmol of **2**.

Encouraged by the above results, we further examined the reaction generality by employing diazo compounds bearing different substituents as substrates (Scheme 3). First, the stable diazo ester (**1aa**) was used as a substrate in this electrochemical transformation under the standard conditions (Scheme 3a), which did not afford desired bromination product **4**. Then, the reaction of the α-diazo ester bearing a phenyl group (**1ab**) with CBr_4_ was carried out. Interestingly, α-diazo-α-phenyl ester (**1ab**) was compatible with the procedure to generate desired product **5** in 57% yield. After careful isolation of the reaction mixture, α-keto ester **6** was also obtained in 10% yield, which was generated from the reaction of diazo with oxygen.^11a^ It is worth mentioning that introducing a phenylcarbonyl group onto the diazo phosphonate (**1ac**) was also successful, and the reaction generated desired α-bromo-β-keto phosphonate **7** in 23% yield. Finally, β-diazo-α,α-difluoroethylphosphonate (**1ad**) was also assayed in this electrochemical reaction. No desired bromination product was detected with all of the diazo **1ad** consumed, and ketone product **8** was obtained in 28% yield.

**Scheme 3:**
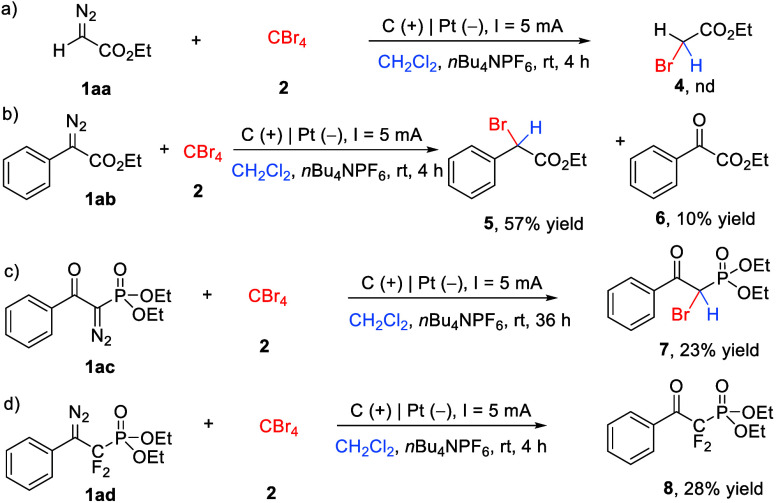
Reaction of Other Diazo Compounds

To determine whether the diazo substrates are oxidized at the anode, several control experiments, as well as the cyclic voltammetry experiments, were carried out. First, a reaction between diazo phosphonate **1a** and CBr_4_ **2** was conducted under the standard conditions but without passing any electricity (Scheme 4a). The corresponding bromination of diazo **3a** was not observed, with almost all of the starting materials remaining. Then, a radical-trapping reaction of diazo **1a** and CBr_4_ **2** was carried out with the addition of 10.0 equiv of radical scavenger 2,2,6,6-tetramethyl-1-piperidinyl-N-oxyl (TEMPO). The reaction was totally suppressed, and no desired product **3a** was obtained (Scheme 4b). TEMPO-trapped dichloromethyl adduct **9** was detected by ESI-MS. These results clearly reveal that this electrochemical transformation involves a dichloromethyl radical species. Then, radical trapping experiments with the addition of 10.0 equiv of 1,1-diphenylethene (DPE) or butylated hydroxytoluene (BHT) were conducted. The formation of desired bromination product **3a** was inhibited in these two reactions. Fortunately, DPE-trapped tribromomethyl radical **10** was detected by ESI-MS, which indicates that the tribromomethyl radical may be generated in this electrochemical process. Also, radical intermediate **11** generated from the bromination of diazo substrate **1a** was detected, and the result discloses that the α-bromo phosphonate radical is involved in this electrochemical process.

**Scheme 4:**
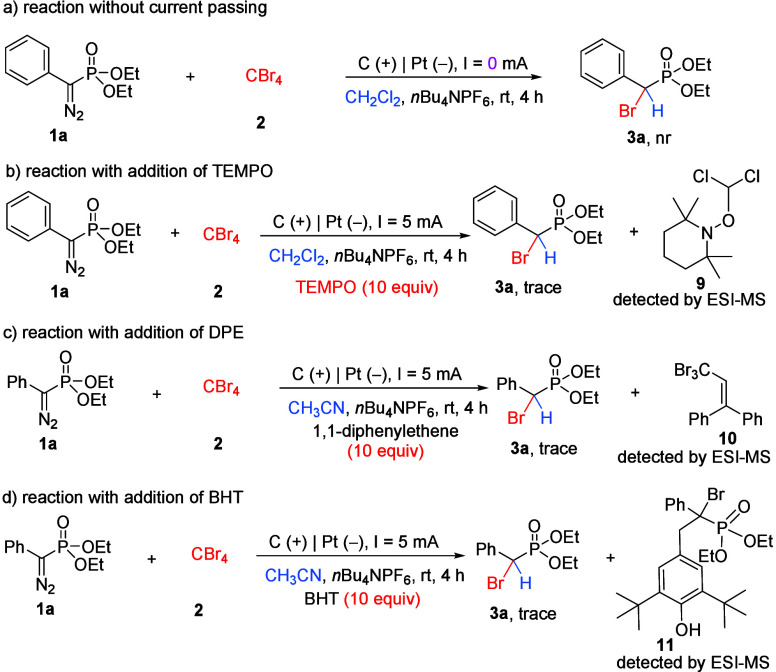
Control Experiments

Subsequently, to gain information about the oxidation and reduction potentials of substrates and reagents in this electrochemical process, cyclic voltammetry experiments (CV) were conducted to elucidate the possible mechanism, and the results are shown in Figure 1. As shown in Figure 1a, there was an oxidation peak of diazo **1a** observed at 2.0 V (vs Ag/AgCl), whereas no oxidation peak was observed for CBr_4_. On the other hand, there was a reductive peak at – 1.7 V (vs Ag/AgCl), while there were no reduction peaks observed for diazo **1a** (Figure 1b). These results disclose that this reaction should involve the anodic oxidation of diazo **1a** and the cathodic reduction of CBr_4_.^14^

**Figure 1:**
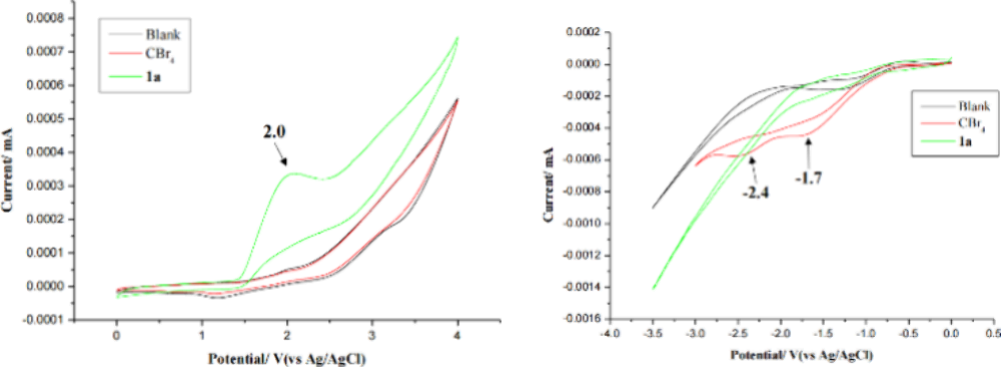
Cyclic voltammetry experiments. (Left) Oxidation. (Right) Reduction.

Based on the above experimental results and related literature reports,^7–11,14^ combined with B3LYP-D3/6-311+G­(d,p) density functional theory (DFT) calculations applying the SMD model to mimic the use of CH_2_Cl_2_ as a solvent,^15^ a plausible reaction mechanism was proposed in Scheme 5. Initially, diazo substrate **1a** undergoes anodic oxidation at the anode to generate radical cation intermediate **A** with the release of N_2_. This process proceeds via a transition state with an energy barrier of 28.3 kcal/mol, delivering radical cation intermediate **A** in an endergonic step (Δ*G* = 11.0 kcal/mol). On the other hand, the cationic reduction of CBr_4_ occurs to generate a bromine anion and CBr_3_ radical.^14^ The coupling reaction of radical cation intermediate **A** with a bromine anion gives radical intermediate **B** in a highly exergonic step (Δ*G* = – 59.0 kcal/mol). In a subsequent step, generated radical intermediate **B** absorbs the hydrogen atom from dichloromethane to generate desired product **3a** (pathway a) and forms the dichloromethyl radical, which was detected by ESI-MS. The Gibbs energy associated with this final step was found to be 8.8 kcal/mol. In an alternative pathway (pathway b), radical cation intermediate **A** may absorb the hydrogen atom from dichloromethane, leading to the formation of cation intermediate **C**. According to DFT calculations, the formation of cationic intermediate **C** was found to proceed with an exergonicity of −10.7 kcal/mol. Then, intermediate **C** undergoes nucleophilic attack by a bromine anion to afford desired product **3a** (pathway b) with an exergonicity of −41.4 kcal/mol. DFT calculations suggest that a radical cation can form without immediate N_2_ release, but this pathway has a higher barrier (Δ*G* ^⧧^ = 34.3 kcal/mol) than that of the prompt N_2_ extrusion route (Δ*G* ^⧧^ = 28.3 kcal/mol), making it less favorable.

**Scheme 5:**
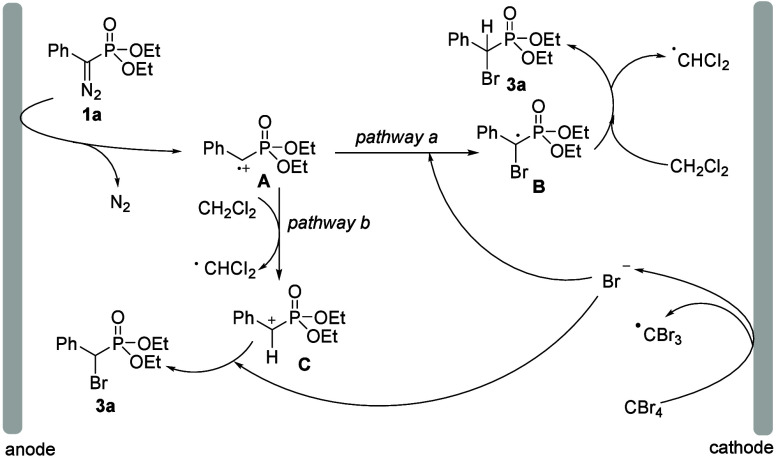
Proposed Mechanism

We also investigated by means of DFT calculations the reactivity of several diazo compounds bearing different substituents by analyzing the energy barrier associated with the transition state, which delivers radical cation intermediate **A** (Figure 2). As mentioned above, the activation Gibbs energy for the transition state of diazo phosponate **1a** was computed to be 28.3 kcal/mol. In contrast, for diazo ester (**1aa**) a Gibbs energy barrier of 37.5 kcal/mol was computed, which is in line with the experimental observation of no formation of desired bromination product **4**. However, for α-diazo-α-phenyl ester (**1ab**), the barrier was lower (Δ*G* ^⧧^ = 28.8 kcal/mol), showing the feasibility for the reaction under study, in agreement with the obtained 57% yield of **5**. Interestingly, when introducing a phenylketo group to the diazo phosphonate (**1ac**) which experimentally afforded desired product **7** with 23% yield, it was found to have an activation Gibbs energy of 33.0 kcal/mol. Finally, for fluorinated diazo compound β-diazo-α,α-difluoroethyl­phosphonate (**1ad**), a barrier of 29.0 kcal/mol was computed.

**Figure 2:**
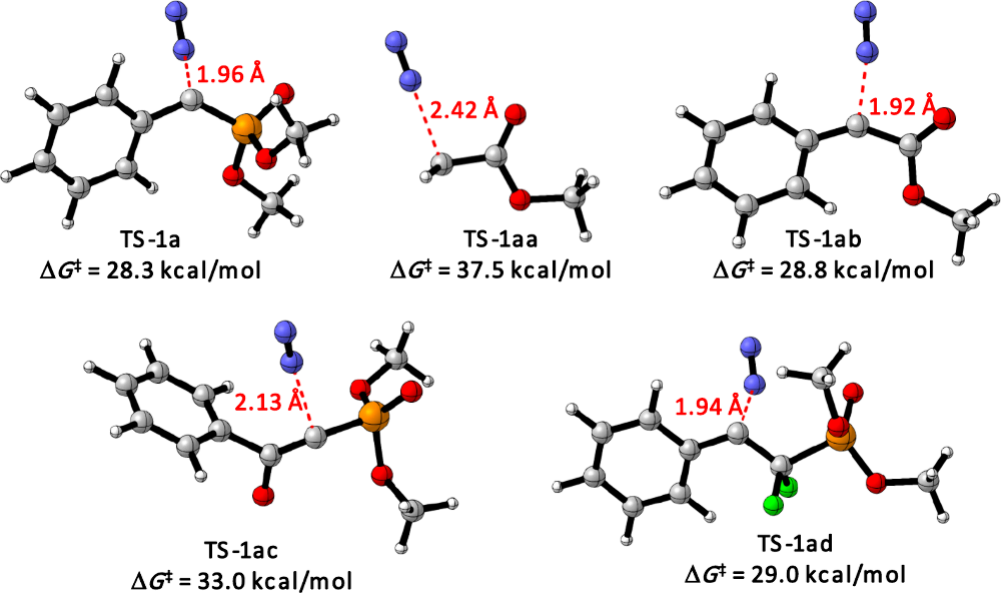
DFT-optimized geometries of transition states and DFT-computed activation Gibbs energies. Bond lengths are shown in Å, and activation Gibbs energies, in kcal/mol.

In summary, we have developed for the first time an electrochemical bromination reaction of diazo compounds via convergent paired electrolysis, affording α-bromo phosphonates as products in up to 96% yield. Combined experimental and computational studies disclose that the reaction proceeds via direct anodic oxidation to generate carbon radical cations to couple with the bromide anion generated at the cathode by using CH_2_Cl_2_ as a hydrogen source. This reaction represents a new electrochemical mode of diazo compounds and affords a sustainable and practical strategy for the synthesis of α-bromo phosphonates.

## Supplementary Material



## Data Availability

The data underlying this study are available in the published article and its Supporting Information.

## Notes

Experimental procedures; details of DFT calculation; full spectroscopic data for compounds **3** and **5**–**8**; and copies of ^1^H NMR, ^19^F NMR, ^31^P NMR, and ^13^C NMR spectra (PDF)

## References

[1] Nawrat C. C., Moody C. J. (2011). Natural products containing a diazo group. Nat. Prod. Rep..

[2] Mix K. A., Aronoff M. R., Raines R. T. (2016). Diazo Compounds: Versatile Tools
for Chemical Biology. ACS Chem. Biol..

[3] Cheng Q., Deng Y., Lankelma M., Doyle M. P. (2017). Cycloaddition
Reactions of Enol-Diazo Compounds. Chem. Soc.
Rev..

[4] Zhang Z., Gevorgyan V. (2024). Visible Light-Induced
Reactions of
Diazo Compounds and Their Precursors. Chem.
Rev..

[5] Khanal H. D., Thombal R. S., Maezono S. M. B., Lee Y. R. (2018). Designs and Strategies
for the Halo-Functionalization of Diazo Compounds. Adv. Synth. Catal..

[6] Zeng L., Wang J., Wang D., Yi H., Lei A. (2023). Comprehensive Comparisons between Directing and Alternating Current
Electrolysis in Organic Synthesis. Angew. Chem.,
Int. Ed..

[7] He Z., Zhao W., Li Y., Yu Y., Huang F. (2022). Electrochemical
S–H and O–H insertion reactions from thiols or salicylic
acids with diazo esters. Org. Biomol. Chem..

[8] Yang D., Guan Z., Peng Y., Zhu S., Wang P., Huang Z., Alhumade H., Gu D., Yi H., Lei A. (2023). Electrochemical oxidative difunctionalization of diazo
compounds
with two different nucleophiles. Nature Commun..

[9] Zhan L., Tao Y. C., Gao L., He M. X., Pan Y. M., Zhang Y., Ma X. L., Mo Z. Y. (2024). Electrochemical
Oxidative Difunctionalization of Diazo Compounds with Diselenides
and Nucleophiles. Org. Lett..

[10] Rybicka-Jasińska K., Szeptuch Z., Kubiszewski H., Kowaluk A. (2023). Electrochemical Cycloaddition
Reactions of Alkene Radical Cations: A Route toward Cyclopropanes
and Cyclobutanes. Org. Lett..

[11] Ruan M., Chen L., Wen Z., Yang F., Ma C., Lu C., Yang G., Gao M. (2022). Electrochemical two-electron oxygen reduction reaction (ORR) induced
aerobic oxidation of α-diazoesters. Chem.
Commun..

[12] He J., Zhou X., Mei A., Makarem A., Javahershenas R., Soloshonok V. A., Han J. (2025). Electrochemical reaction of indole-tethered alkynes enabling stereoselective
synthesis of iodovinyl spiroindolenine-cyclopentanes. Chem. Commun..

[13] Mei H., Wang L., Pajkert R., Wang Q., Xu J., Liu J., Röschenthaler G. V., Han J. (2021). In Situ Generation of Unstable Difluoromethylphosphonate-Containing
Diazoalkanes and Their Use in [3 + 2] Cycloaddition Reactions with
Vinyl Sulfones. Org. Lett..

[14] Zhou Z., Yuan Y., Cao Y., Qiao J., Yao A., Zhao J., Zuo W., Chen W., Lei A. (2019). Synergy of
Anodic Oxidation and Cathodic Reduction Leads to Electrochemical C—H
Halogenation. Chin. J. Chem..

[15] See the Supporting Information for computational details.

