# Supplementary material for: Bromination Functionalization of Diazo Compounds with CBr4 via Convergent Paired Electrolysis
Source: Org Lett. 2026 Jan 13;28(3):1025–30. doi: 10.1021/acs.orglett.5c05051 (PMC12848972; doi:10.1021/acs.orglett.5c05051)

Supporting Information

Bromination Functionalization of Diazo Compounds with CBr_4 via Convergent Paired Electrolysis

Qian Wang,† Wentian Wu,† Haibo Mei,*,† Jorge Escorihuela,*,‡ Romana Pajkert,§ Gerd-Volker Röschenthaler,*,§ and Jianlin Han*,†

† Jiangsu Co-Innovation Center of Efficient Processing and Utilization of Forest Resources, College of Chemical Engineering, Nanjing Forestry University, Nanjing 210037, China.

‡ Department of Organic Chemistry, Faculty of Pharmacy and Food Sciences, Universitat de València, Avda. Vicente Andrés Estellés s/n, Burjassot 46100, Valencia, Spain.

§ School of Science, Constructor University Bremen gGmbH, Campus Ring 1, 28759 Bremen, Germany

Email: meihb@njfu.edu.cn; Jorge.Escorihuela@uv.es; hanjl@njfu.edu.cn

Table of Contents

1. General information……………………………………………………………..…………S2

2. Graphical guide for the set-up……..……………………….………………………………S3

3. General procedure for the electrochemical reaction…………………….………..……....S5

4. General procedure for the cyclic voltammetry (CV) experiments …………………..……….S6

5. Control experiments……………………………………………………………………...S9

6. Scale-up synthesis……………………………………..…………………….………………..S13

7. General procedure for the synthesis of diazo compounds..………………….……………..S13

8. Differential scanning calorimetry (DSC) analysis of compound 1a………..…………………S16

9. Computational information……………………………………………..…….…….…..S17

10. Characterization data of 3, 5, 6, 7 and 8………………………………………..….…..…..S29

11. ^{1}H, ^{13}C, ^{19}F and ^{31}P NMR spectra for compound 3, 5, 6, 7 and 8……….…………….................S41

1. General information

The instrument for electrolysis is DC power supply UTP1303 made in China. The size of electrodes is 1.0 × 1.0 cm^2. The carbon electrodes were fabricated by cutting a 10 cm × 10 cm carbon sheet into 1 cm × 1 cm segments. Faraday of electrons per mole of substrate is 7.5 F/mol under constant current conditions. All the commercial reagents including solvents were used directly without further purification. Compounds **1ab, 1ac, 1ad** were synthesized according to the literature reports.[1-3] All α-aryl-α-diazophosphonates **1** were prepared following a literature procedure.[4] All the experiments were monitored by thin layer chromatography (TLC) with UV light. The TLC employed 0.25 mm silica gel coated on glass plates. Purification of products was carried out by silica gel 60 F-254 TLC plates of 20 cm × 20 cm and column chromatography with silica gel 60 (300-400 mesh). NMR spectra were recorded on Bruker 400 MHz and 600 MHz spectrometers. High resolution mass spectra (HRMS) were measured on Agilent 6210 ESI/TOF MS instrument.

2. Graphical guide for the set-up

As experimental set-up, the anode electrode was graphite flakes (10 mm × 10 mm × 1 mm) and cathode electrode was platinum sheet (10 mm × 10 mm × 0.1 mm), rubber plugs, an undivided three-necked bottle were used.

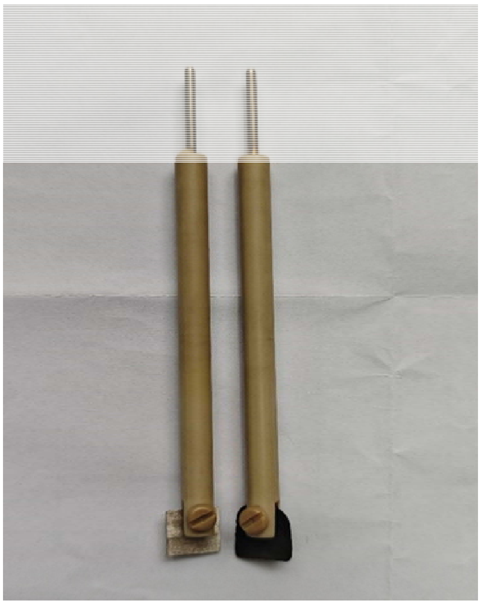

A) A graphite anode (1.0 cm × 1.0 cm × 1 mm) and a platinum sheet cathode (1.0 cm × 1.0 cm × 0.1 mm)

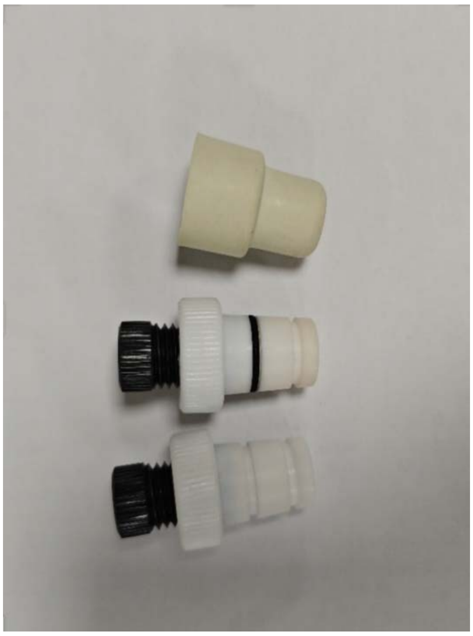

B) Plastic plugs and rubber plug

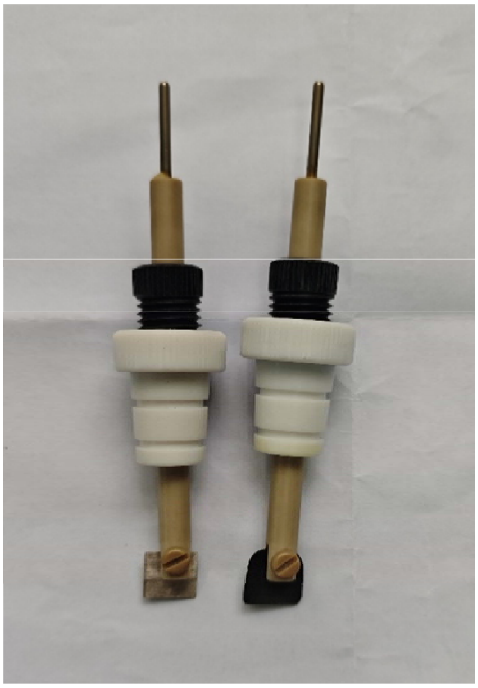

C) Graphite anode and cathode

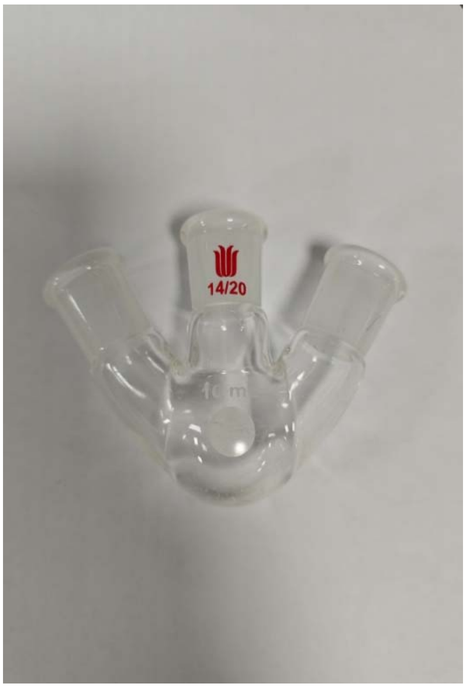

D) Electrochemical cell

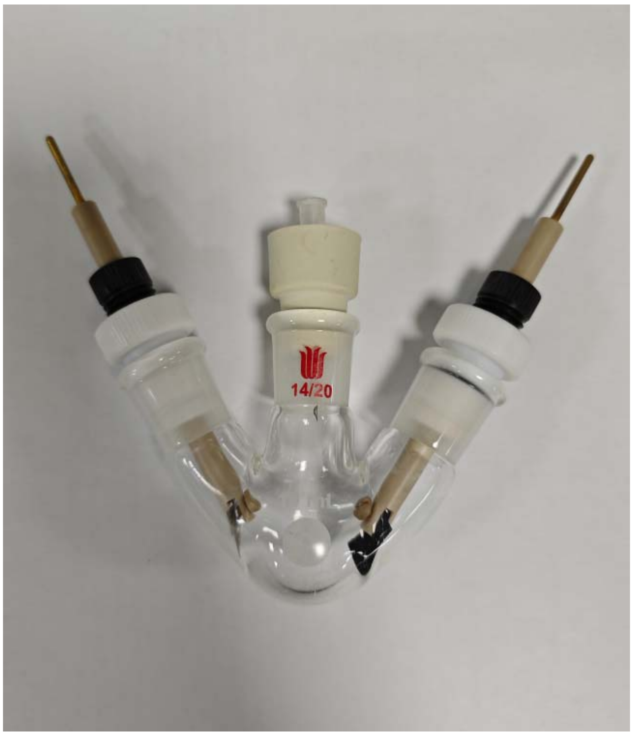

E) Electrochemical cell

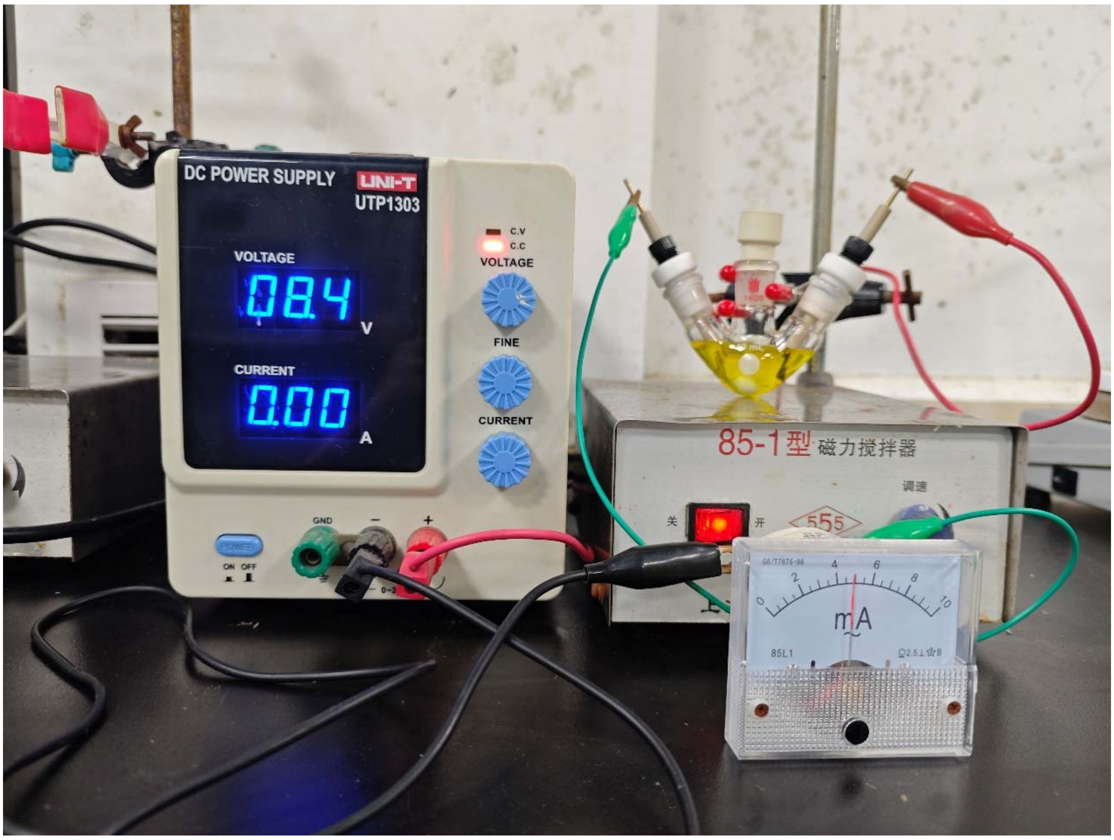

Figure S1. Graphical guide for the set-up.

3. General procedure for the electrochemical reaction

In an oven-dried undivided three-necked bottle equipped with a stir bar, diazo **1** (0.3 mmol, 3.0 equiv.), CBr_4 **2** (0.1 mmol), $TBAPF_6$ (0.7 mmol), DCM (12 mL, 0.008 M) were added. The bottle was equipped with a carbon anode (1.0 cm × 1.0 cm × 1 mm) and a platinum sheet cathode (1.0 cm × 1.0 cm × 0.1 mm). The distance between the working electrode and the counter electrode was 1.0 cm. The reaction mixture was stirred and electrolyzed at a constant current of 5 mA for 4 h at room temperature. Finally, upon completion of the reaction as determined by TLC, the reaction mixture was concentrated on a rotary evaporator. The resulting residue was directly purified by TLC plate of 20 cm × 20 cm using petroleum ether/ethyl acetate (4:1, v/v) as eluent to afford the corresponding product **3**.

4. General procedure for the cyclic voltammetry (CV) experiments

The cyclic voltammetry experiments were carried out with a computer-controlled electrochemical analyzer for electrochemical measurements. The cyclic voltammetry experiments were measured at room temperature. Cyclic voltammetry (CV) was performed on an electrochemical workstation (Chenhua CHI760E). The experiment was performed in a three-electrode cell with DCM (12 mL) containing 0.7 mmol $TBAPF_6$ as blank background. The concentration of the CBr_4 was 0.008 M; the concentration of the 1a was 0.025 M. The scan speed was 200 mV/s. The potential ranges investigated were 0 to 4.0 V (initial potential: 0 V; direction of initial scan: oxidative; switching potential: 4.0 V; final potential: 0 V) vs. Ag/AgCl (saturated aqueous KCl (3 M). CV plotting convention is IUPAC.

Working electrode: The working electrode is a platinum wire (0.5 mm diameter, 35 mm length) working electrode, which was washed with water and ethanol before measurements.

Reference electrode: The reference electrode is Ag/AgCl (saturated aqueous KCl (3 M), which was washed with water and ethanol before measurements.

Counter electrode: The counter electrode is a platinum flake cylindrical (2 mm diameter) working electrode, which was washed with water and ethanol before measurements.

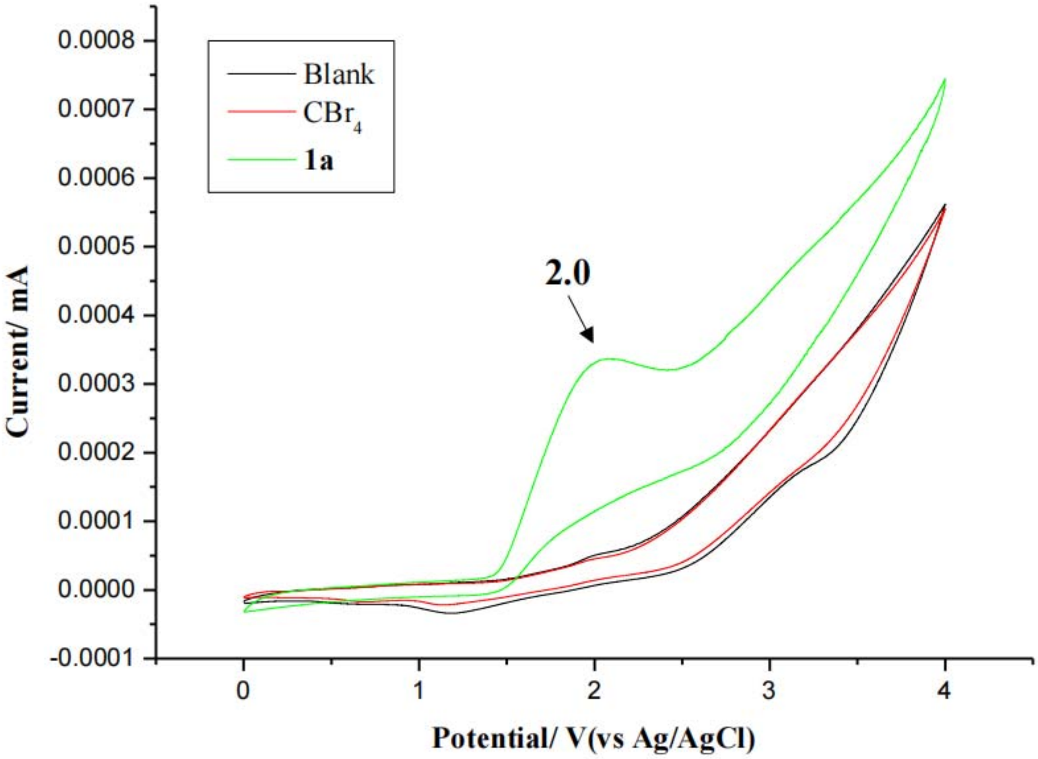

Figure S2. Cyclic voltammetry experiment (oxidation) (the direction of scan is positive direction).

The cyclic voltammetry experiments were carried out with a computer-controlled electrochemical analyzer for electrochemical measurements. The cyclic voltammetry experiments were measured at room temperature. Cyclic voltammetry (CV) was performed on an electrochemical workstation (Chenhua CHI760E). The experiment was performed in a three-electrode cell with DCM (12 mL) containing 0.7 mmol $TBAPF_6$ as blank background. The concentration of the CBr_4 was 0.008 M; the concentration of the 1a was 0.025 M. The scan speed was 200 mV/s. The potential ranges investigated were -3.5 V to 0 V (initial potential: 0 V; direction of initial scan: reductive; switching potential: -3.5 V for $TBAPF_6$ and **1a**, -3.0 V for CBr_4; final potential: 0 V) vs. Ag/AgCl (saturated aqueous KCl (3 M). CV plotting convention is IUPAC.

Working electrode: The working electrode is a platinum wire (0.5 mm diameter, 35 mm length) working electrode, which was washed with water and ethanol before measurements.

Reference electrode: The reference electrode is Ag/AgCl (saturated aqueous KCl (3 M), which was

washed with water and ethanol before measurements.

Counter electrode: The counter electrode is a platinum flake cylindrical (2 mm diameter) working electrode, which was washed with water and ethanol before measurements.

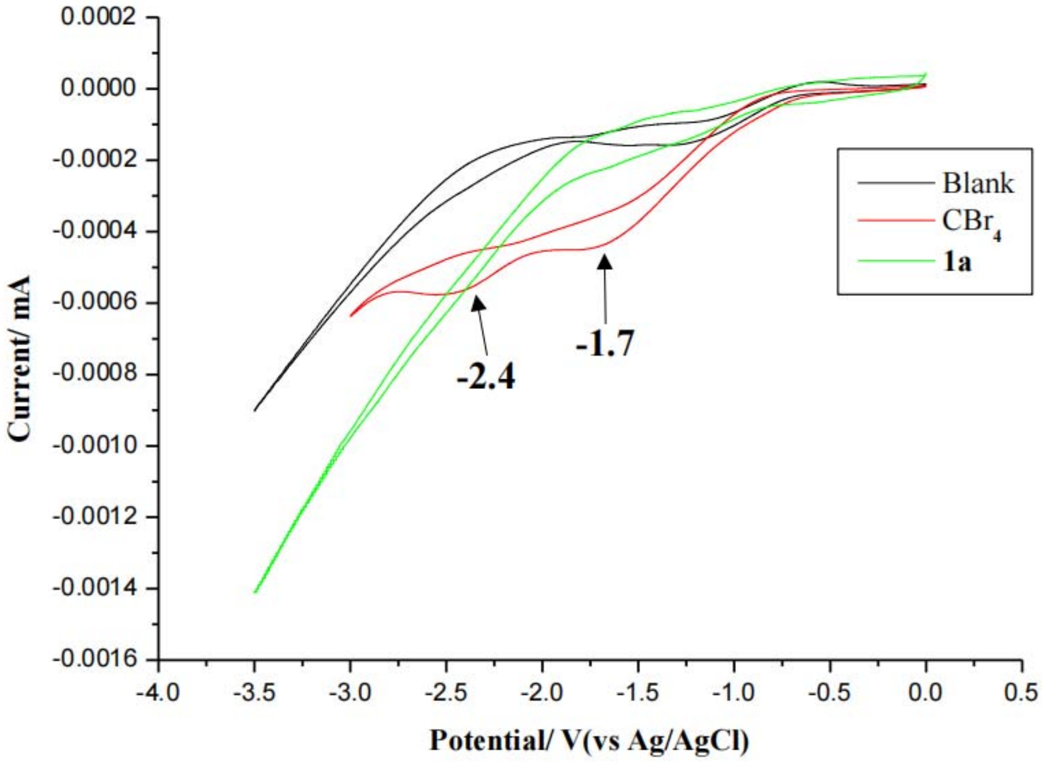

Figure S3. Cyclic voltammetry experiment (reduction) (the direction of scan is negative direction).

5. Control experiments

5.1. reaction without current passing

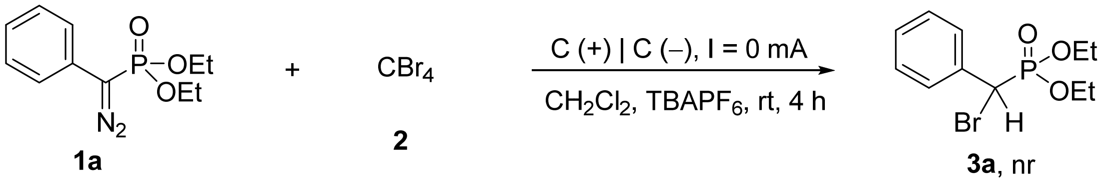

In an oven-dried undivided three-necked bottle equipped with a stir bar, diethyl (diazo(phenyl)methyl)phosphonate **1a** (76.3 mg, 0.3 mmol, 3.0 equiv.), tetrabromomethane **2** (33.2 mg, 0.1 mmol), $TBAPF_6$ (271.2 mg, 0.7 mmol), DCM (12 mL) were added. The bottle was equipped with a carbon anode (1.0 cm × 1.0 cm × 1 mm) and a platinum sheet cathode (1.0 cm × 1.0 cm × 0.1 mm). The reaction without current passing and stirred for 4 h at room temperature. Finally, almost no conversion of diethyl (diazo(phenyl)methyl)phosphonate **1a** and no target product **3a** was formed.

5.2. Radical-trapping experiment with TEMPO

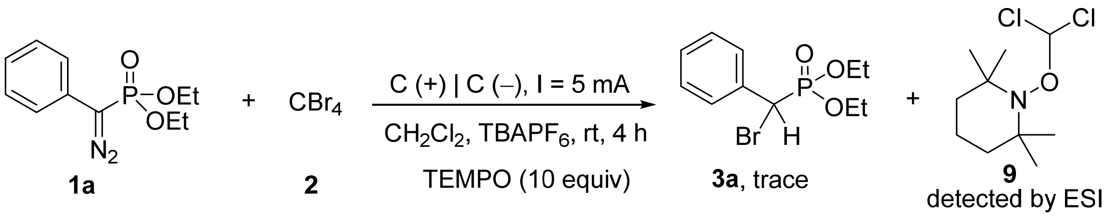

In an oven-dried undivided three-necked bottle equipped with a stir bar, diethyl (diazo(phenyl)methyl)phosphonate **1a** (76.3 mg, 0.3 mmol, 3.0 equiv.), tetrabromomethane **2** (33.2 mg, 0.1 mmol), $TBAPF_6$ (271.2 mg, 0.7 mmol), TEMPO (156.2 mg, 1.0 mmol, 10.0 equiv.), DCM (12 mL) were added. The bottle was equipped with a carbon anode (1.0 cm × 1.0 cm × 1 mm) and a platinum sheet cathode (1.0 cm × 1.0 cm × 0.1 mm). The reaction mixture was stirred and electrolyzed at a constant current of 5 mA for 4 h at room temperature. The reaction was analyzed by ESI-MS analysis of the crude reaction mixture.

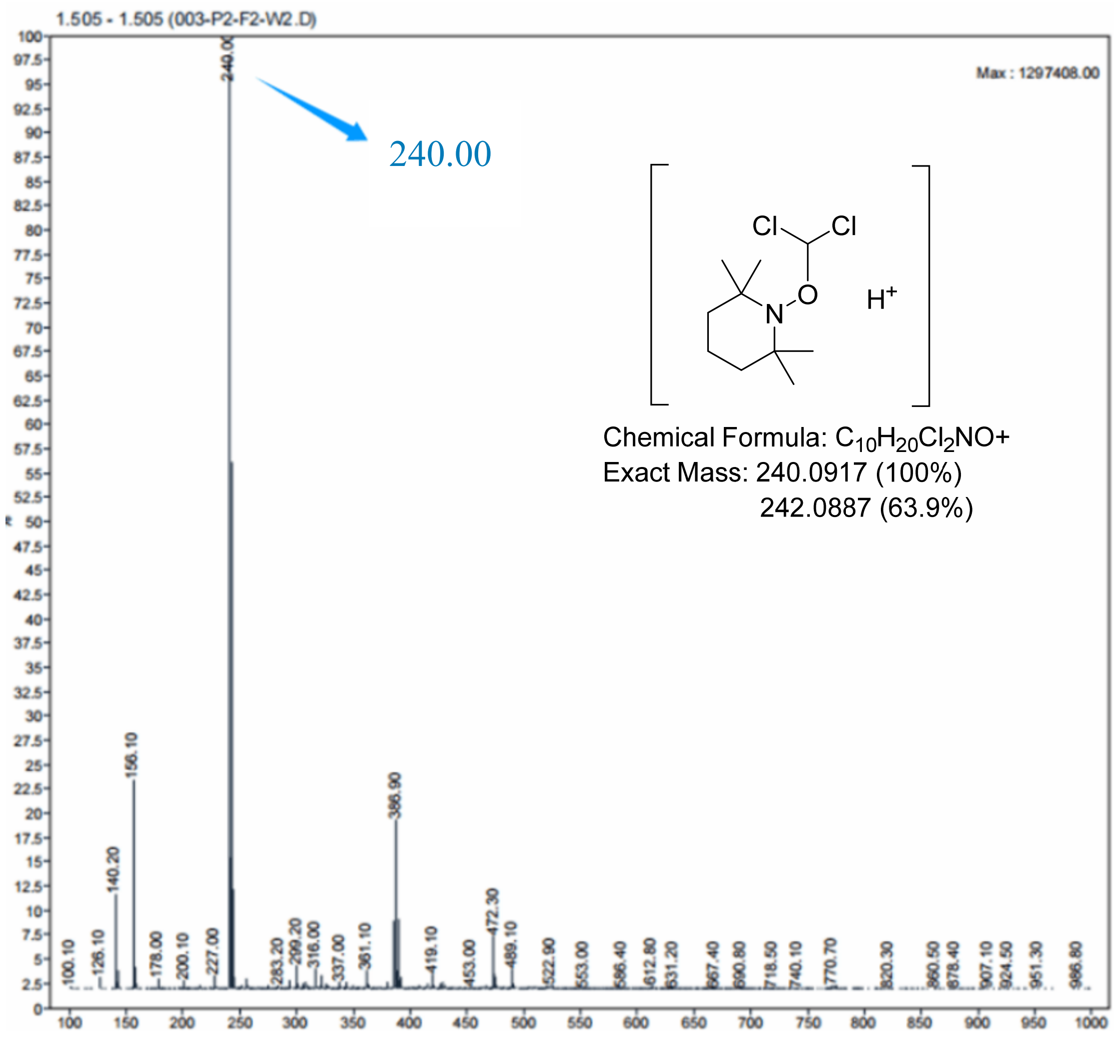

Figure S4. ESI-MS detection of TEMPO-trapping adduct.

5.3. Radical-trapping experiment with DPE

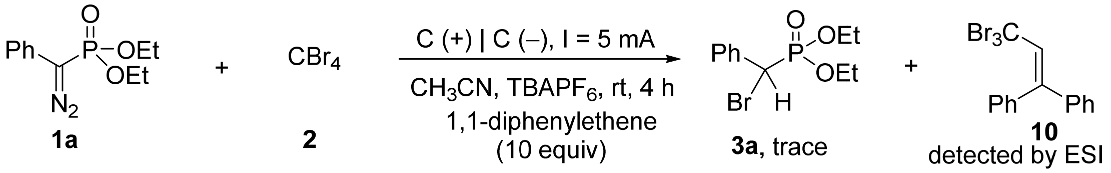

In an oven-dried undivided three-necked bottle equipped with a stir bar, diethyl (diazo(phenyl)methyl)phosphonate **1a** (76.3 mg, 0.3 mmol, 3.0 equiv.), tetrabromomethane **2** (33.2 mg, 0.1 mmol), $TBAPF_6$ (271.2 mg, 0.7 mmol), DPE (180.2 mg, 1.0 mmol, 10.0 equiv.), MeCN (12 mL) were added. The bottle was equipped with a carbon anode (1.0 cm × 1.0 cm × 1 mm) and a platinum sheet cathode (1.0 cm × 1.0 cm × 0.1 mm). The reaction mixture was stirred and electrolyzed at a constant current of 5 mA for 4 h at room temperature. The reaction was analyzed

by ESI-MS analysis of the crude reaction mixture.

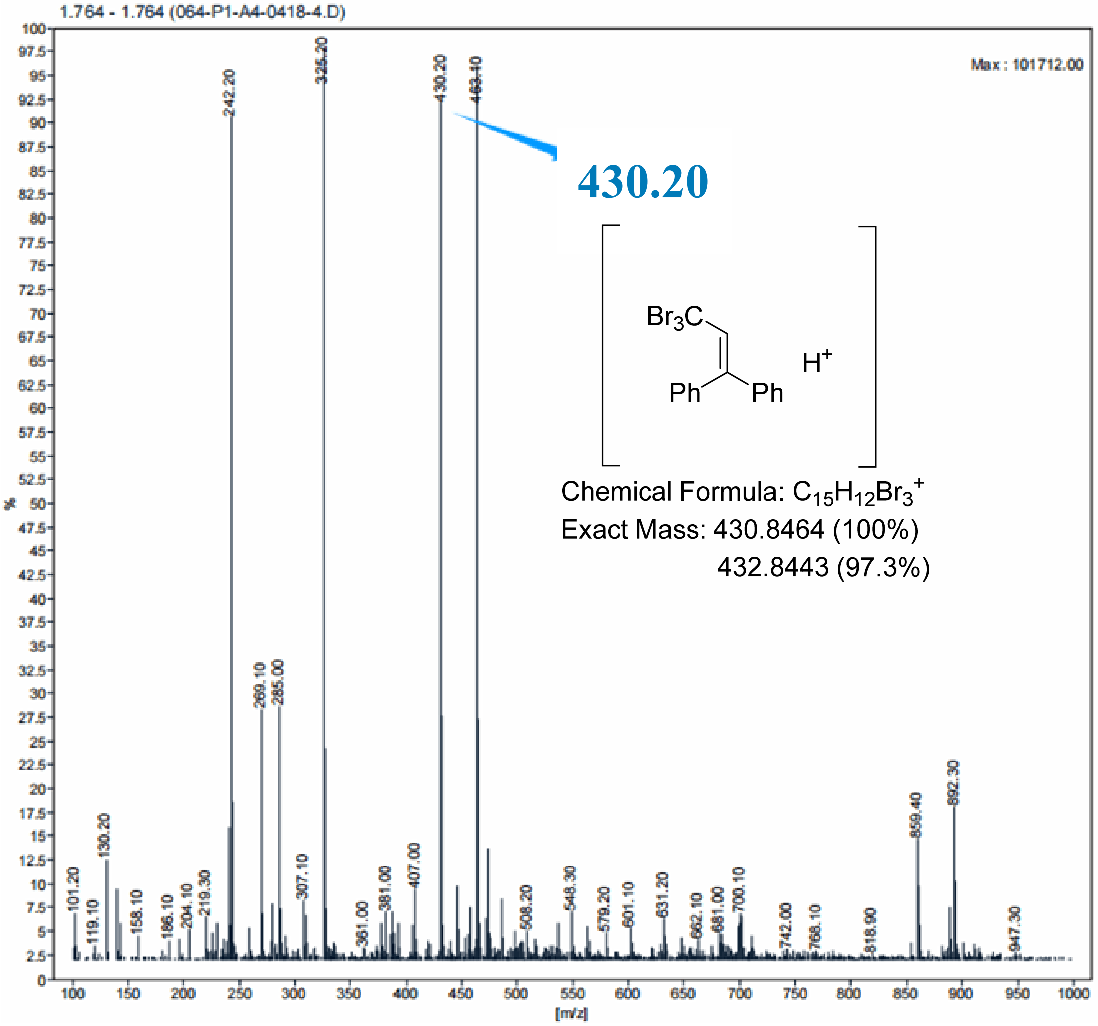

Figure S5. ESI-MS detection of DPE-trapping adduct.

5.4. Radical-trapping experiment with BHT

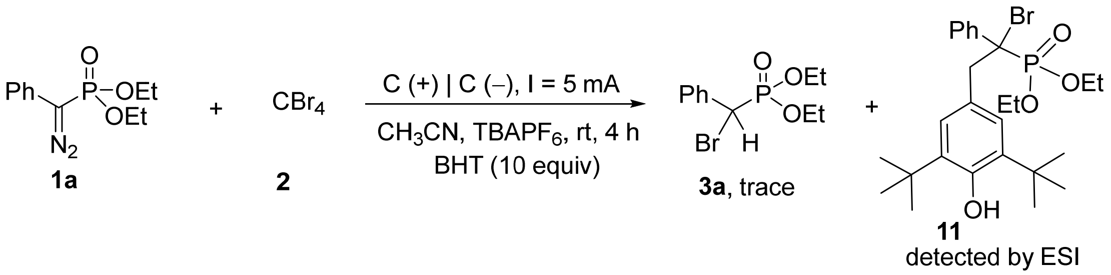

In an oven-dried undivided three-necked bottle equipped with a stir bar, diethyl (diazo(phenyl)methyl)phosphonate **1a** (76.3 mg, 0.3 mmol, 3.0 equiv.), tetrabromomethane **2** (33.2 mg, 0.1 mmol), $TBAPF_6$ (271.2 mg, 0.7 mmol), BHT (220.4 mg, 1.0 mmol, 10.0 equiv.), MeCN

(12 mL) were added. The bottle was equipped with a carbon anode (1.0 cm × 1.0 cm × 1 mm) and a platinum sheet cathode (1.0 cm × 1.0 cm × 0.1 mm). The reaction mixture was stirred and electrolyzed at a constant current of 5 mA for 4 h at room temperature. The reaction was analyzed by ESI-MS analysis of the crude reaction mixture.

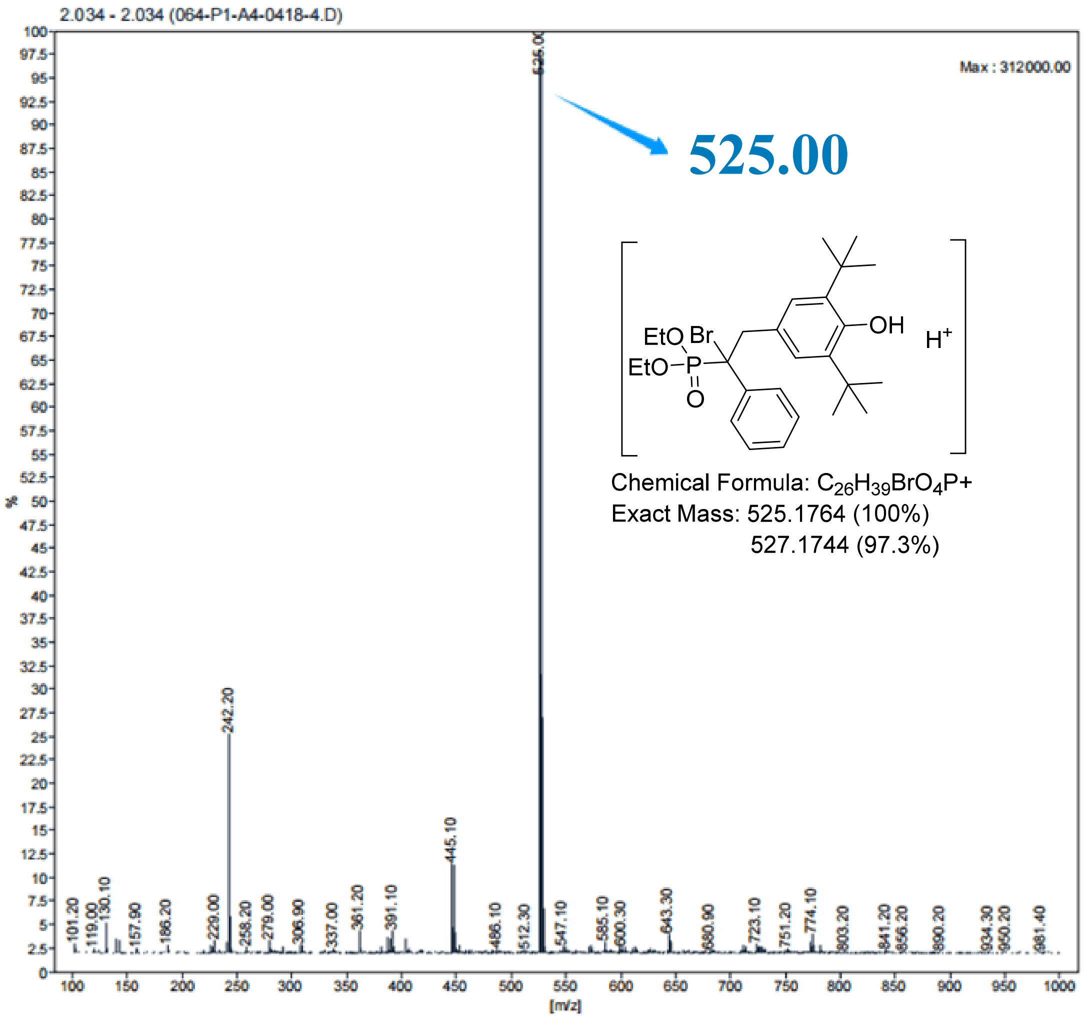

Figure S6. ESI-MS detection of BHT-trapping adduct.

6. Scale-up synthesis

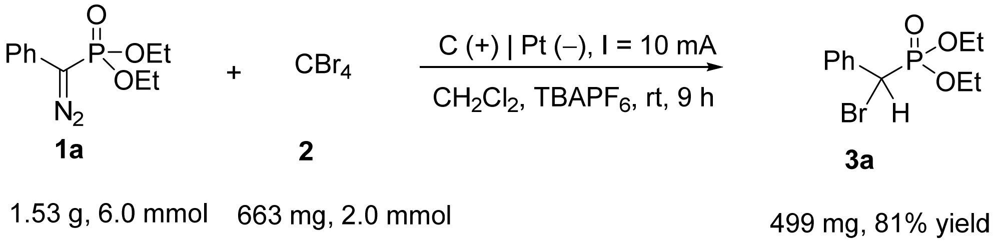

In an oven-dried undivided three-necked bottle equipped with a stir bar, diethyl (diazo(phenyl)methyl)phosphonate **1a** (1.53 g, 6.0 mmol, 3.0 equiv.), tetrabromomethane **2** (663 mg, 2.0 mmol), $TBAPF_6$ (5.42g, 14.0 mmol), DCM (150 mL) were added. The bottle was equipped with a carbon anode (1.0 cm × 1.0 cm × 1 mm) and a platinum sheet cathode (1.0 cm × 1.0 cm × 0.1 mm). The reaction mixture was stirred and electrolyzed at a constant current of 10 mA for 9 h at room temperature. Finally, upon completion of the reaction as determined by TLC, the reaction mixture was concentrated on a rotary evaporator. The resulting residue was directly purified by column chromatography using petroleum ether/ethyl acetate (4:1, v/v) as eluent to afford the corresponding product **3a** (499.3 mg, 81%).

7. General procedure for the synthesis of diazo compounds

7.1. general method for the synthesis of N-tosylhydrazones[4a]

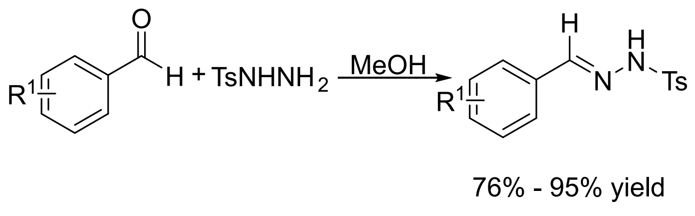

A solution of pure $TsNHNH_2$ (1.86 g, 10.0 mmol) in methanol (10.0 mL, 1 M) was stirred and heated to 60 °C until the $TsNHNH_2$ was completely dissolved. The mixture was cooled to room temperature, and then carbonyl compounds (10.0 mmol, 1.0 equiv.) were dropped into the mixture

slowly. After approximately 30 min the mixture was cooled to 0 °C and the product removed by filtration, washed with petroleum ether then evaporated under vacuum to afford the pure products. The yields ranged from 76% to 95%.

7.2. general method for the coupling of H-phosphonates with N-tosylhydrazones[4a]

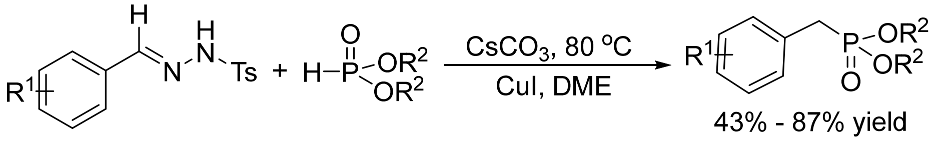

An oven-dried Schlenk tube containing CuI (190.5 mg, 1.0 mmol, 10 mol%), *N*-tosylhydrazones (10.0 mmol), Cs_2CO_3 (4.89 g, 15.0 mmol, 1.5 equiv.) was evacuated and purged with nitrogen three times. Freshly distilled DME (25 mL, 0.4 M) and diethyl *H*-phosphonate (10.0 mmol, 1.0 equiv.) were sequentially added to the system at room temperature. The reaction mixture was heated with stirring at 80 °C for 2 h. The reaction mixture was allowed to cool to ambient temperature, and then transferred to a round-bottom flask. Silica gel (10.0 g) was added, and the solvent was removed under reduced pressure to afford a free-flowing powder. This powder was then dry-loaded onto a silica gel column and purified by flash chromatography using petroleum ether:AcOEt (2:1, v/v) as the eluent to give diethyl benzylphosphonate. The yields ranged from about 43% to 87%.

7.3. general method for the synthesis of α-aryl-α-diazo-phosphonates[4b,c]

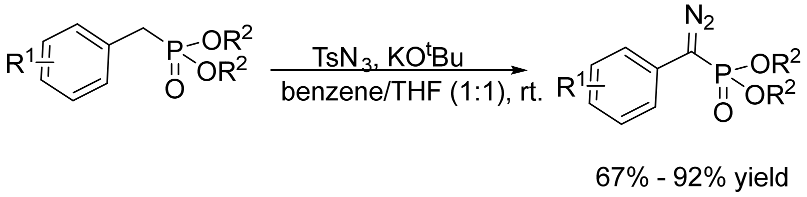

Benzylphosphonate (10.0 mmol) and TsN_3 (2.56 g, 13.0 mmol) were dissolved in benzene (60.0 mL, 0.16 M) and a solution of KO*t*Bu (2.81 g, 25.0 mmol, 1.9 equiv.) in THF (60.0 mL) was added

dropwise for 30 min at r.t. After addition, the reaction mixture was stirred at r.t. for 60 min. Petroleum ether (50.0 mL) was added and the precipitate was filtered off. The solvent was removed under reduced pressure and diazo was isolated by chromatography on a column with PE/EtOAc mixture (3:1 to 1:1 v/v). The yields ranged from 67% to 92%.

8. Differential scanning calorimetry (DSC) analysis of compound 1a

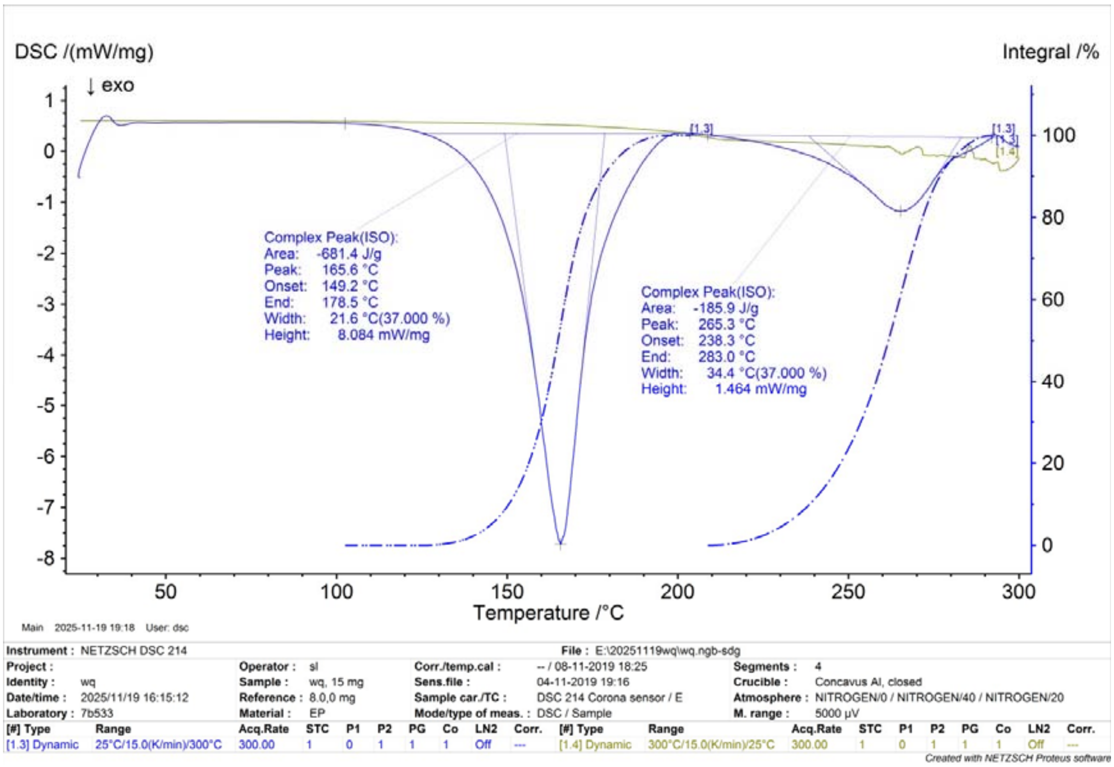

Figure S7. DSC analysis of diazo compound **1a**.

9. Computational information

9.1. Computational details.

All density functional theory calculations were performed using Gaussian16.[5] Geometry optimizations were performed using the B3LYP hybrid functional[6] including Grimme's dispersion (D3 damping function)[7] with the 6-311+G(d,p)[8] basis set in dichloromethane using the SMD solvent model[9] to evaluate the effect of the solvent. Frequency calculations of reactants and transition states were performed at the same level of theory. The optimized geometries were confirmed to be minima (no imaginary frequencies) or transition structures (one imaginary frequency) by frequency calculations. Intrinsic reaction coordinate (IRC) calculations were performed to verify the expected connections of the first-order saddle points with the local minima found on the potential energy surface.[10] CYLview was used to create images of optimized structures.[11]

9.2. Computed energies and cartesian coordinates of optimized structures.

1a

E(electronic) = -1026.36568240
Zero-point correction= 0.195618 (Hartree/Particle)
Thermal correction to Energy= 0.211414
Thermal correction to Enthalpy= 0.212358
Thermal correction to Gibbs Free Energy= 0.150917
Sum of electronic and zero-point Energies= -1026.170064
Sum of electronic and thermal Energies= -1026.154268
Sum of electronic and thermal Enthalpies= -1026.153324
Sum of electronic and thermal Free Energies= -1026.214765

6	-0.033954000	0.876247000	-0.173353000
6	-1.348694000	0.210841000	-0.096654000
6	-2.541624000	0.950494000	-0.188576000
6	-1.434849000	-1.181546000	0.074756000
6	-3.777449000	0.316187000	-0.107144000
6	-2.676599000	-1.806830000	0.154939000
6	-3.854830000	-1.066279000	0.065869000
1	-2.511115000	2.025682000	-0.326524000
1	-0.536998000	-1.779860000	0.149049000
1	-4.683284000	0.907968000	-0.180294000
1	-2.718590000	-2.882288000	0.288596000
1	-4.818718000	-1.558080000	0.129642000
15	1.593683000	0.123455000	-0.116226000
8	2.677622000	1.141260000	-0.141805000
8	1.612240000	-0.869884000	1.152622000
6	1.681283000	-0.336611000	2.498791000
1	2.610050000	0.218830000	2.642147000
1	0.820838000	0.308277000	2.696502000
1	1.655215000	-1.197443000	3.164734000
8	1.534874000	-0.936715000	-1.311981000
6	2.521387000	-1.996370000	-1.445084000
1	3.533104000	-1.585754000	-1.430397000
1	2.394899000	-2.724583000	-0.642406000
1	2.326845000	-2.463426000	-2.408727000
7	0.005505000	2.179912000	-0.284577000
7	0.025435000	3.306484000	-0.376978000

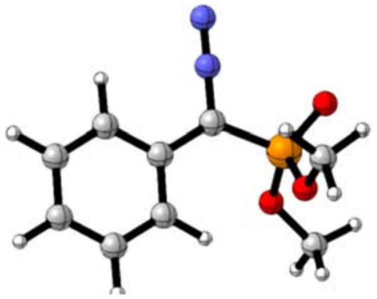

TS-1a

E(electronic) = -1026.30860596
Zero-point correction= 0.191662 (Hartree/Particle)
Thermal correction to Energy= 0.208087
Thermal correction to Enthalpy= 0.209031
Thermal correction to Gibbs Free Energy= 0.145575
Sum of electronic and zero-point Energies= -1026.116944
Sum of electronic and thermal Energies= -1026.100519
Sum of electronic and thermal Enthalpies= -1026.099575
Sum of electronic and thermal Free Energies= -1026.163031
Frequency -387.3516

6	0.004983000	0.639893000	-0.571001000
6	1.362583000	0.275516000	-0.202273000
6	2.398661000	0.782909000	-1.020562000
6	1.711758000	-0.580659000	0.869950000
6	3.723531000	0.437565000	-0.791298000
6	3.036542000	-0.935072000	1.088194000
6	4.041399000	-0.425441000	0.261026000
1	2.134352000	1.441242000	-1.840251000
1	0.935282000	-0.956646000	1.525201000
1	4.507472000	0.829591000	-1.428981000
1	3.292483000	-1.598604000	1.906352000
1	5.075512000	-0.700337000	0.438433000
15	-1.393872000	-0.285053000	0.076389000
8	-1.521029000	-0.715931000	1.503667000
8	-2.621674000	0.616430000	-0.442706000
6	-3.956589000	0.379463000	0.067456000
1	-4.299219000	-0.622971000	-0.201231000
1	-3.981553000	0.504141000	1.151667000
1	-4.594460000	1.123274000	-0.407606000
8	-1.390510000	-1.528025000	-0.968758000
6	-0.834031000	-2.813566000	-0.602616000
1	0.257726000	-2.766455000	-0.589911000
1	-1.205521000	-3.130944000	0.373188000
1	-1.161076000	-3.512897000	-1.371050000
7	-0.332483000	2.314608000	0.295497000
7	-0.341836000	3.417243000	0.271284000

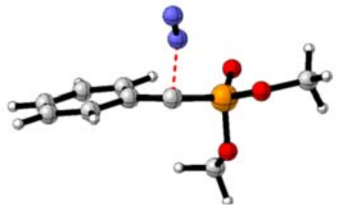

A

E(electronic) = -916.766848732

Zero-point correction= 0.184648 (Hartree/Particle)

Thermal correction to Energy= 0.198708

Thermal correction to Enthalpy= 0.199652

Thermal correction to Gibbs Free Energy= 0.142040

Sum of electronic and zero-point Energies= -916.582200

Sum of electronic and thermal Energies= -916.568141

Sum of electronic and thermal Enthalpies= -916.567197

Sum of electronic and thermal Free Energies= -916.624808

6	-0.096217000	-0.978519000	0.096072000
6	-1.400874000	-0.456459000	0.024365000
6	-2.463982000	-1.409064000	-0.074269000
6	-1.753471000	0.932041000	0.044179000
6	-3.784636000	-1.006396000	-0.153008000
6	-3.075497000	1.325323000	-0.027098000
6	-4.087393000	0.359596000	-0.126987000
1	-2.200947000	-2.460082000	-0.085513000
1	-0.968665000	1.673811000	0.111651000
1	-4.580551000	-1.737517000	-0.229848000
1	-3.335039000	2.377263000	-0.007894000
1	-5.122739000	0.678093000	-0.184673000
15	1.388627000	-0.051911000	0.304080000
8	1.832159000	-0.131563000	1.733321000
8	2.439158000	-0.686800000	-0.751649000
6	3.052225000	-1.973271000	-0.502478000
1	3.651278000	-1.938973000	0.409988000
1	2.290411000	-2.751781000	-0.419902000
1	3.694737000	-2.174665000	-1.358845000
8	1.288526000	1.443927000	-0.306074000

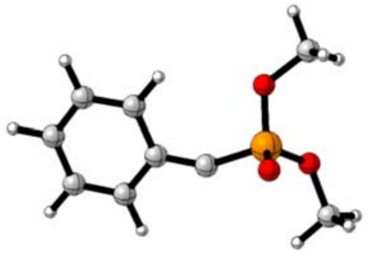

6	2.455158000	2.297729000	-0.420407000
1	2.955567000	2.398877000	0.545526000
1	3.146944000	1.892963000	-1.161735000
1	2.088972000	3.270286000	-0.746960000

B

E(electronic) = -3490.99307359

Zero-point correction= 0.186379 (Hartree/Particle)
Thermal correction to Energy= 0.202071
Thermal correction to Enthalpy= 0.203015
Thermal correction to Gibbs Free Energy= 0.139709
Sum of electronic and zero-point Energies= -3490.806694
Sum of electronic and thermal Energies= -3490.791003
Sum of electronic and thermal Enthalpies= -3490.790058
Sum of electronic and thermal Free Energies= -3490.853364

6	-0.053729000	0.500952000	-0.065781000
6	-1.329008000	-0.151154000	-0.067464000
6	-2.553040000	0.566079000	0.029474000
6	-1.414192000	-1.570771000	-0.168251000
6	-3.772477000	-0.091301000	0.031666000
6	-2.641306000	-2.213525000	-0.166264000
6	-3.830329000	-1.484899000	-0.065377000
1	-2.538337000	1.644403000	0.109800000
1	-0.515959000	-2.163035000	-0.257352000
1	-4.686836000	0.485969000	0.110495000
1	-2.672509000	-3.294148000	-0.249240000
1	-4.786748000	-1.994828000	-0.064390000
15	1.555315000	-0.324890000	-0.000765000
8	2.712281000	0.580064000	0.218694000
8	1.420193000	-1.502498000	1.097441000
6	1.497369000	-1.197089000	2.511442000
1	2.452478000	-0.724945000	2.749245000
1	0.671338000	-0.544690000	2.807350000
1	1.413275000	-2.150195000	3.030841000
8	1.546180000	-1.180988000	-1.356253000
6	2.615082000	-2.117045000	-1.661494000
1	3.584080000	-1.614697000	-1.633217000
1	2.597615000	-2.949493000	-0.955403000
1	2.415834000	-2.478658000	-2.668594000
35	0.062760000	2.401073000	-0.140423000

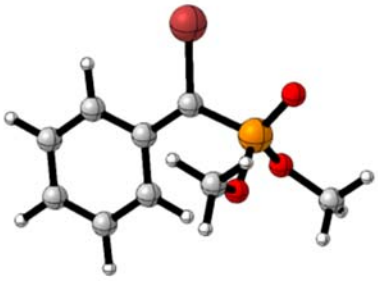

C

E(electronic) = -917.252584699

Zero-point correction= 0.198032 (Hartree/Particle)
Thermal correction to Energy= 0.212172
Thermal correction to Enthalpy= 0.213117
Thermal correction to Gibbs Free Energy= 0.155783
Sum of electronic and zero-point Energies= -917.054553
Sum of electronic and thermal Energies= -917.040412
Sum of electronic and thermal Enthalpies= -917.039468
Sum of electronic and thermal Free Energies= -917.096802

6	-0.116217000	-0.891264000	0.032749000
6	-1.416121000	-0.445344000	-0.006406000
6	-2.457368000	-1.434537000	-0.149271000
6	-1.783083000	0.944481000	0.096920000
6	-3.774648000	-1.051372000	-0.192307000
6	-3.106919000	1.302673000	0.058183000
6	-4.096921000	0.312563000	-0.087251000
1	-2.175336000	-2.478334000	-0.222275000
1	-1.011298000	1.693553000	0.204886000
1	-4.561520000	-1.786654000	-0.301493000
1	-3.395991000	2.342697000	0.139275000
1	-5.138865000	0.611734000	-0.117732000
15	1.453602000	-0.018698000	0.299277000
8	1.826599000	-0.146448000	1.731637000
8	2.460874000	-0.679110000	-0.748009000
6	3.241294000	-1.869947000	-0.428461000
1	3.908040000	-1.663536000	0.408650000
1	2.579953000	-2.705759000	-0.191655000
1	3.815716000	-2.091668000	-1.325099000
8	1.206115000	1.422110000	-0.314301000
6	2.280431000	2.402463000	-0.470154000
1	2.765595000	2.584046000	0.489852000
1	2.998710000	2.042690000	-1.207180000
1	1.795697000	3.309138000	-0.824274000
1	0.043883000	-1.968147000	-0.020745000

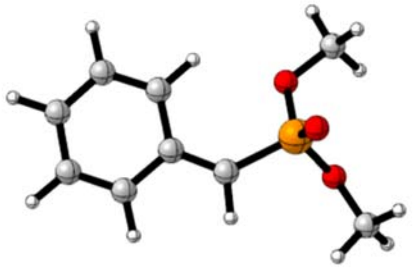

3a

E(electronic) = -3491.64563461

Zero-point correction= 0.199087 (Hartree/Particle)

Thermal correction to Energy= 0.214925

Thermal correction to Enthalpy= 0.215869

Thermal correction to Gibbs Free Energy= 0.152683

Sum of electronic and zero-point Energies= -3491.446548

Sum of electronic and thermal Energies= -3491.430710

Sum of electronic and thermal Enthalpies= -3491.429766

Sum of electronic and thermal Free Energies= -3491.492952

6	0.052414000	-0.323983000	0.591236000
6	1.477095000	-0.017185000	0.243869000
6	2.393926000	0.195561000	1.279447000
6	1.909378000	0.070642000	-1.086645000
6	3.723055000	0.502196000	0.991489000
6	3.237081000	0.373755000	-1.370940000
6	4.147053000	0.591639000	-0.333629000
1	2.065386000	0.123112000	2.310795000
1	1.205106000	-0.097060000	-1.892563000
1	4.424732000	0.667587000	1.801378000
1	3.563125000	0.440802000	-2.402843000
1	5.181274000	0.827374000	-0.558837000
15	-1.193514000	0.794259000	-0.169569000
8	-1.235057000	0.834230000	-1.655185000
8	-2.595671000	0.425100000	0.527971000
6	-3.650225000	-0.297524000	-0.157911000
1	-3.637019000	-0.077771000	-1.226180000
1	-3.523418000	-1.368271000	0.007490000
1	-4.588248000	0.040088000	0.281022000
8	-0.777425000	2.145351000	0.585551000
6	-1.470899000	3.392780000	0.320311000

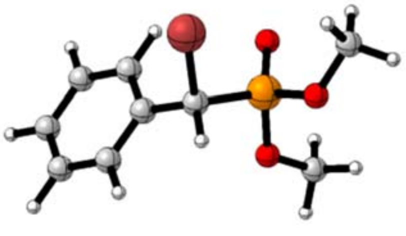

1	-1.370705000	3.666920000	-0.731703000
1	-2.525379000	3.305375000	0.591546000
1	-0.989108000	4.141839000	0.945916000
1	-0.105837000	-0.291329000	1.667568000
35	-0.452721000	-2.213065000	0.091996000

CH_2Cl_2

E(electronic) = -959.778402488

Zero-point correction= 0.029288 (Hartree/Particle)

Thermal correction to Energy= 0.032896

Thermal correction to Enthalpy= 0.033840

Thermal correction to Gibbs Free Energy= 0.003081

Sum of electronic and zero-point Energies= -959.749114

Sum of electronic and thermal Energies= -959.745507

Sum of electronic and thermal Enthalpies= -959.744563

Sum of electronic and thermal Free Energies= -959.775321

6	0.000000000	0.000000000	0.779989000
1	0.901913000	0.000000000	1.383220000
1	-0.901913000	0.000000000	1.383220000
17	0.000000000	1.497430000	-0.219011000
17	0.000000000	-1.497430000	-0.219011000

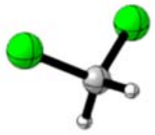

$CHCl_2$ radical

E(electronic) = -959.109890717

Zero-point correction= 0.015530 (Hartree/Particle)

Thermal correction to Energy= 0.019207

Thermal correction to Enthalpy= 0.020152

Thermal correction to Gibbs Free Energy= -0.011786

Sum of electronic and zero-point Energies= -959.094361

Sum of electronic and thermal Energies= -959.090683

Sum of electronic and thermal Enthalpies= -959.089739

Sum of electronic and thermal Free Energies= -959.121676

6	0.011859000	0.701909000	0.000000000
1	-0.474361000	1.668603000	0.000000000
17	0.011859000	-0.172943000	1.485211000
17	0.011859000	-0.172943000	-1.485211000

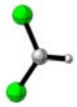

1aa

E(electronic) = -376.750871448

Zero-point correction= 0.075864 (Hartree/Particle)

Thermal correction to Energy= 0.083120

Thermal correction to Enthalpy= 0.084064

Thermal correction to Gibbs Free Energy= 0.044234

Sum of electronic and zero-point Energies= -376.675007

Sum of electronic and thermal Energies= -376.667751

Sum of electronic and thermal Enthalpies= -376.666807

Sum of electronic and thermal Free Energies= -376.706638

6	0.845513000	-0.798393000	-0.000010000
6	-0.322925000	0.063884000	-0.000085000
8	-0.307246000	1.281919000	-0.000068000
8	-1.446914000	-0.679602000	-0.000072000
6	-2.693842000	0.051407000	0.000099000
1	-2.772681000	0.672301000	0.894206000
1	-2.770738000	0.676414000	-0.891270000
1	-3.472269000	-0.709172000	-0.002465000
7	2.016667000	-0.221059000	0.000026000
7	3.019101000	0.295128000	0.000070000
1	0.826127000	-1.877943000	-0.000053000

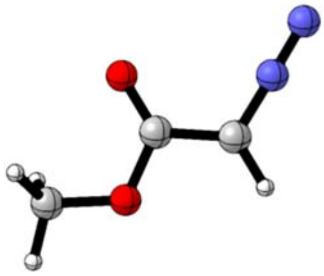

TS-1aa

E(electronic) = -376.679275128

Zero-point correction= 0.070086 (Hartree/Particle)

Thermal correction to Energy= 0.078658

Thermal correction to Enthalpy= 0.079603

Thermal correction to Gibbs Free Energy= 0.035418

Sum of electronic and zero-point Energies= -376.609189

Sum of electronic and thermal Energies= -376.600617

Sum of electronic and thermal Enthalpies= -376.599672

Sum of electronic and thermal Free Energies= -376.643857

Frequency -112.8744

6	-0.400641000	1.236951000	0.232316000
1	-0.648638000	1.749636000	-0.706613000
6	0.520957000	0.134557000	0.014423000
8	0.186372000	-1.035738000	-0.016434000
8	1.778038000	0.594475000	-0.032003000
6	2.846394000	-0.393627000	0.003804000
1	2.770250000	-0.997061000	0.909219000
1	2.798305000	-1.030416000	-0.880020000
1	3.766524000	0.185976000	0.007158000
7	-2.557260000	0.101355000	-0.181242000
7	-3.471595000	-0.422115000	0.117597000

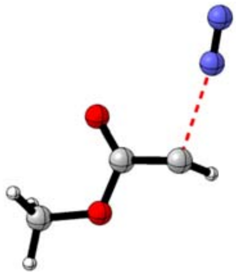

1ab

E(electronic) = -607.876677926

Zero-point correction= 0.157024 (Hartree/Particle)

Thermal correction to Energy= 0.168767

Thermal correction to Enthalpy= 0.169711

Thermal correction to Gibbs Free Energy= 0.118165

Sum of electronic and zero-point Energies= -607.719654

Sum of electronic and thermal Energies= -607.707911

Sum of electronic and thermal Enthalpies= -607.706967

Sum of electronic and thermal Free Energies= -607.758513

6	-0.576289000	0.692603000	-0.098636000
6	0.805794000	0.165128000	-0.044192000
6	1.828475000	0.925048000	0.546302000
6	1.125148000	-1.082704000	-0.601790000
6	3.136858000	0.449107000	0.576767000
6	2.433426000	-1.557821000	-0.555671000
6	3.445511000	-0.796382000	0.029876000
1	1.602728000	1.887716000	0.993046000
1	0.352318000	-1.677908000	-1.068616000
1	3.912797000	1.049733000	1.038345000

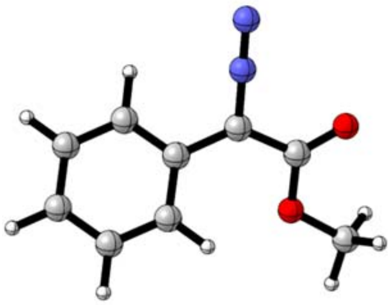

1	2.662745000	-2.524929000	-0.989632000
1	4.463077000	-1.169322000	0.059249000
6	-1.857686000	-0.013373000	0.009657000
8	-2.945545000	0.529054000	-0.080564000
8	-1.689541000	-1.324991000	0.235506000
6	-2.896536000	-2.111353000	0.364114000
1	-3.485530000	-1.767850000	1.215971000
1	-3.491934000	-2.047846000	-0.547933000
1	-2.555572000	-3.132041000	0.525362000
7	-0.726025000	1.993859000	-0.222249000
7	-0.852281000	3.110201000	-0.324023000

TS-1ab

E(electronic) = -607.825501709

Zero-point correction= 0.153050 (Hartree/Particle)

Thermal correction to Energy= 0.165426

Thermal correction to Enthalpy= 0.166370

Thermal correction to Gibbs Free Energy= 0.112812

Sum of electronic and zero-point Energies= -607.672451

Sum of electronic and thermal Energies= -607.660076

Sum of electronic and thermal Enthalpies= -607.659132

Sum of electronic and thermal Free Energies= -607.712690

Frequency -343.9741

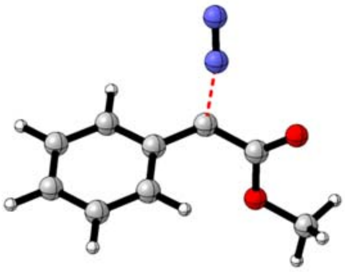

6	0.546800000	0.561862000	-0.538535000
6	-0.776760000	0.073580000	-0.222181000
6	-1.864591000	0.534982000	-0.994833000
6	-1.024024000	-0.869262000	0.801909000
6	-3.151253000	0.069660000	-0.756988000
6	-2.308018000	-1.345699000	1.026628000
6	-3.371300000	-0.873395000	0.250479000
1	-1.675876000	1.257605000	-1.780586000
1	-0.200162000	-1.224971000	1.409967000
1	-3.980506000	0.429026000	-1.355287000
1	-2.488830000	-2.075099000	1.807878000
1	-4.374782000	-1.242671000	0.432095000
6	1.762913000	-0.051233000	-0.025152000
8	2.661833000	0.456673000	0.624230000
8	1.830943000	-1.312312000	-0.529700000
6	3.095126000	-1.994981000	-0.374850000
1	3.893623000	-1.443732000	-0.875412000
1	3.338961000	-2.117244000	0.681826000
1	2.955730000	-2.966503000	-0.845577000
7	0.674057000	2.278247000	0.355240000
7	0.631125000	3.378269000	0.326189000

1ac

E(electronic) = -1139.73366534

Zero-point correction= 0.205290 (Hartree/Particle)

Thermal correction to Energy= 0.222979

Thermal correction to Enthalpy= 0.223923

Thermal correction to Gibbs Free Energy= 0.157406

Sum of electronic and zero-point Energies= -1139.528375

Sum of electronic and thermal Energies= -1139.510686

Sum of electronic and thermal Enthalpies= -1139.509742

Sum of electronic and thermal Free Energies= -1139.576260

6	0.327978000	-0.151198000	-0.116812000
6	-0.732104000	0.804454000	0.254063000
8	-0.418201000	1.924446000	0.633690000
15	2.073376000	0.137829000	0.253651000
8	2.368061000	0.475301000	1.668907000
8	2.784718000	-1.202118000	-0.292993000
6	2.924048000	-2.369885000	0.561039000
1	3.572549000	-2.142852000	1.408244000
1	1.948507000	-2.710902000	0.917855000
1	3.376466000	-3.139959000	-0.061296000
8	2.495413000	1.215144000	-0.847316000
6	3.742219000	1.954004000	-0.718110000
1	3.732926000	2.551204000	0.194918000
1	4.594128000	1.270431000	-0.717678000
1	3.789976000	2.604598000	-1.589203000
7	0.052872000	-1.202189000	-0.853929000
7	-0.157403000	-2.107264000	-1.488631000
6	-2.163401000	0.388429000	0.149135000
6	-2.596045000	-0.894429000	0.508683000
6	-3.098420000	1.344700000	-0.269320000
6	-3.950133000	-1.216187000	0.443892000
6	-4.446766000	1.013136000	-0.351372000
6	-4.874448000	-0.267500000	0.006208000
1	-1.889482000	-1.632138000	0.871521000
1	-2.757586000	2.338999000	-0.532638000
1	-4.282545000	-2.204818000	0.739295000
1	-5.164608000	1.751736000	-0.689722000
1	-5.926743000	-0.522893000	-0.050867000

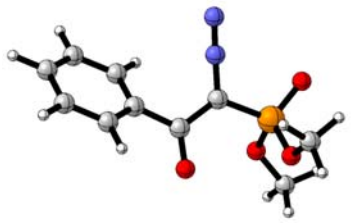

TS-1ac

E(electronic) = -1139.67566044

Zero-point correction= 0.200738 (Hartree/Particle)

Thermal correction to Energy= 0.219438

Thermal correction to Enthalpy= 0.220382

Thermal correction to Gibbs Free Energy= 0.150303

Sum of electronic and zero-point Energies= -1139.474923

Sum of electronic and thermal Energies= -1139.456222

Sum of electronic and thermal Enthalpies= -1139.455278

Sum of electronic and thermal Free Energies= -1139.525358

Frequency -193.4320

6	0.437115000	0.176808000	-0.857157000
6	-0.721646000	-0.642392000	-0.901944000
8	-0.475526000	-1.683465000	-1.538213000
15	2.035055000	-0.354783000	-0.242298000
8	2.761457000	-1.136497000	-1.277332000
8	2.769573000	0.990162000	0.239267000
6	3.339097000	1.915564000	-0.723763000
1	4.157320000	1.439566000	-1.266483000
1	2.572575000	2.258666000	-1.422678000
1	3.714460000	2.756276000	-0.143001000
8	1.754912000	-1.071941000	1.161144000
6	2.843528000	-1.616330000	1.959383000
1	3.398793000	-2.361103000	1.386227000
1	3.507095000	-0.814278000	2.288381000
1	2.372824000	-2.086803000	2.820489000

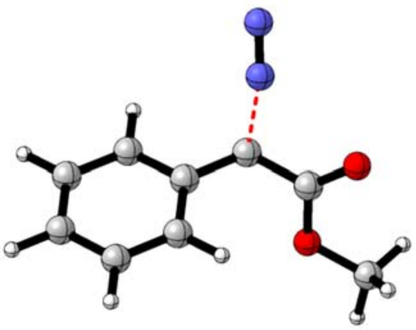

7	0.076477000	1.647719000	0.620773000
7	0.001315000	2.611557000	1.136401000
6	-2.075963000	-0.303381000	-0.407333000
6	-2.618808000	0.980376000	-0.541186000
6	-2.839675000	-1.332376000	0.158750000
6	-3.915716000	1.232228000	-0.101925000
6	-4.128754000	-1.069819000	0.612725000
6	-4.667342000	0.211766000	0.483149000
1	-2.044071000	1.766795000	-1.015253000
1	-2.414715000	-2.325407000	0.248365000
1	-4.341264000	2.222143000	-0.220658000
1	-4.713400000	-1.863034000	1.064531000
1	-5.674337000	0.413249000	0.831193000

1ad

E(electronic) = -1264.21800975

Zero-point correction= 0.206747 (Hartree/Particle)

Thermal correction to Energy= 0.225736

Thermal correction to Enthalpy= 0.226681

Thermal correction to Gibbs Free Energy= 0.157342

Sum of electronic and zero-point Energies= -1264.011263

Sum of electronic and thermal Energies= -1263.992273

Sum of electronic and thermal Enthalpies= -1263.991329

Sum of electronic and thermal Free Energies= -1264.060668

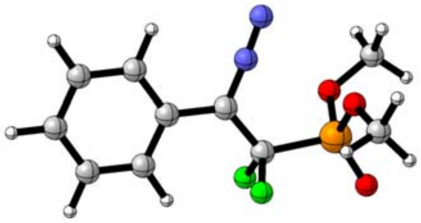

6	-0.662303000	0.337355000	-0.004620000
6	-2.130260000	0.191087000	0.006672000
6	-2.962585000	1.297149000	0.256429000
6	-2.728699000	-1.058414000	-0.232512000
6	-4.346610000	1.155109000	0.266670000
6	-4.115015000	-1.188883000	-0.219618000
6	-4.933469000	-0.087961000	0.029134000
1	-2.536867000	2.276620000	0.443733000
1	-2.122939000	-1.931465000	-0.430098000
1	-4.966850000	2.022919000	0.461848000
1	-4.554166000	-2.162594000	-0.406869000
1	-6.011931000	-0.196366000	0.037572000
15	2.143724000	-0.425882000	-0.191877000
8	2.946936000	-1.648647000	-0.417431000
8	2.204992000	0.758231000	-1.250432000
6	3.349477000	1.652800000	-1.379120000
1	3.445275000	2.257072000	-0.476166000
1	4.260554000	1.079819000	-1.559131000
1	3.130310000	2.285904000	-2.235875000
8	2.375443000	0.328574000	1.199908000
6	2.726290000	-0.380662000	2.424371000
1	1.843145000	-0.880610000	2.825069000
1	3.519187000	-1.103177000	2.229714000
1	3.073209000	0.382725000	3.117836000
7	-0.153725000	1.520986000	0.212031000
7	0.223237000	2.570421000	0.406570000
6	0.292703000	-0.779576000	-0.248961000
9	0.090056000	-1.796768000	0.683086000
9	0.034665000	-1.382761000	-1.472719000

TS-1ad

E(electronic) = -1264.16484574

Zero-point correction= 0.202720 (Hartree/Particle)
Thermal correction to Energy= 0.222352
Thermal correction to Enthalpy= 0.223296
Thermal correction to Gibbs Free Energy= 0.151744
Sum of electronic and zero-point Energies= -1263.962126
Sum of electronic and thermal Energies= -1263.942494
Sum of electronic and thermal Enthalpies= -1263.941550
Sum of electronic and thermal Free Energies= -1264.013102

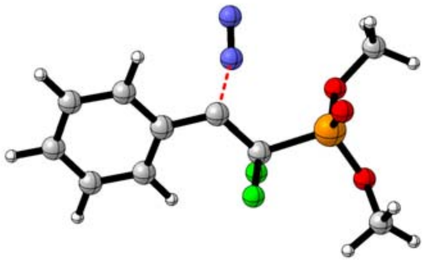

6	-0.748789000	0.295551000	0.518991000
6	-2.142731000	0.021111000	0.230579000
6	-3.090021000	0.771384000	0.967192000
6	-2.620560000	-0.974867000	-0.656067000
6	-4.451364000	0.552173000	0.817296000
6	-3.982826000	-1.215106000	-0.775574000
6	-4.898672000	-0.448265000	-0.050050000
1	-2.727757000	1.526655000	1.655311000
1	-1.926774000	-1.564722000	-1.238053000
1	-5.163966000	1.141216000	1.382948000
1	-4.336705000	-1.992166000	-1.443454000
1	-5.961763000	-0.633665000	-0.158388000
15	2.099722000	-0.053166000	0.286502000
8	2.374365000	0.145698000	1.731060000
8	2.987267000	-1.172327000	-0.441374000
6	3.335737000	-2.425738000	0.211122000
1	2.509823000	-3.132180000	0.115014000
1	4.212178000	-2.802285000	-0.313276000
1	3.573054000	-2.258409000	1.262860000
8	2.266562000	1.200782000	-0.683070000
6	3.137181000	2.320062000	-0.356404000
1	2.844125000	2.759885000	0.597948000
1	4.177708000	1.992503000	-0.321264000
1	3.000273000	3.040700000	-1.160260000
7	-0.388102000	1.944549000	-0.440861000
7	-0.415356000	3.046272000	-0.451028000
6	0.322923000	-0.562802000	-0.081170000
9	0.169581000	-1.797011000	0.540913000
9	0.245682000	-0.826325000	-1.456703000

Alternative TS

E(electronic) = -3600.49680690
Zero-point correction= 0.193204 (Hartree/Particle)
Thermal correction to Energy= 0.211135
Thermal correction to Enthalpy= 0.212079
Thermal correction to Gibbs Free Energy= 0.143792
Sum of electronic and zero-point Energies= -3600.303603
Sum of electronic and thermal Energies= -3600.285672
Sum of electronic and thermal Enthalpies= -3600.284727
Sum of electronic and thermal Free Energies= -3600.353015
Frequency -398.1540

6	-0.085383000	0.484455000	-0.407963000
6	1.345989000	0.100988000	-0.144870000
6	2.131475000	-0.491010000	-1.142902000
6	1.887991000	0.269025000	1.138086000
6	3.437395000	-0.890196000	-0.864847000
6	3.190338000	-0.135772000	1.409720000
6	3.972083000	-0.714952000	0.409288000
1	1.731650000	-0.646198000	-2.135748000
1	1.289163000	0.708622000	1.924533000
1	4.033846000	-1.339297000	-1.650900000
1	3.593675000	0.002373000	2.406486000
1	4.988950000	-1.024924000	0.622195000
15	-1.347014000	-0.777311000	0.136119000
8	-2.731809000	-0.484659000	-0.308599000
8	-0.752057000	-2.174680000	-0.381919000
6	-1.169190000	-2.779637000	-1.635902000
1	-2.251858000	-2.714575000	-1.750580000
1	-0.670096000	-2.285332000	-2.470951000
1	-0.859066000	-3.821653000	-1.580303000
8	-1.035735000	-0.818912000	1.695791000
6	-1.736104000	-1.727573000	2.591170000
1	-2.807568000	-1.520657000	2.577366000
1	-1.540776000	-2.762264000	2.303649000
1	-1.331618000	-1.533378000	3.582375000
7	-0.325876000	0.449332000	-2.059229000
7	-0.722960000	1.330485000	-2.676741000
35	-0.580572000	2.264922000	0.317972000

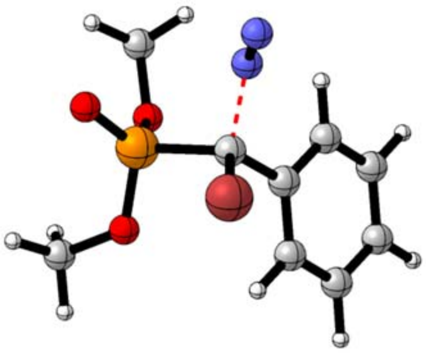

10. Characterization data of 3, 5, 6, 7 and 8

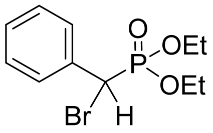

Compound **3a**:[12] purified by TLC plate (petroleum ether : ethyl acetate = 4:1), 29.4 mg, 96% yield, colorless oil. ^{1}H NMR (400 MHz, $CDCl_3$): δ = 7.58 (d, *J* = 7.6 Hz, 2H), 7.40-7.30 (m, 3H), 4.87 (d, *J* = 13.1 Hz, 1H), 4.29-4.17 (m, 2H), 4.13-3.99 (m, 1H), 3.94-3.83 (m, 1H), 1.35 (t, *J* = 7.1 Hz, 3H), 1.16 (t, *J* = 7.1 Hz, 3H). ^{13}C NMR (100 MHz, $CDCl_3$) δ = 134.6 (d, *J* = 3.5 Hz), 129.5 (d, *J* = 6.6 Hz), 129.0 (d, *J* = 2.2 Hz), 128.7 (d, *J* = 1.2 Hz), 64.1 (t, *J* = 6.9 Hz), 41.5 (d, *J* = 159.3 Hz), 16.4 (d, *J* = 6.0 Hz), 16.2 (d, *J* = 5.7 Hz). ^{31}P NMR (162 MHz, $CDCl_3$): δ = 17.15 (s).

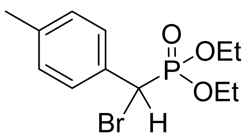

Compound **3b**:[12] purified by TLC plate (petroleum ether : ethyl acetate = 4:1), 23.6 mg, 73% yield, colorless oil. ^{1}H NMR (400 MHz, $CDCl_3$) : δ = 7.46 (d, *J* = 7.1 Hz, 2H), 7.17 (d, *J* = 7.9 Hz, 2H), 4.86 (d, *J* = 12.8 Hz, 1H), 4.27-4.19 (m, 2H), 4.13-4.01 (m, 1H), 3.93-3.86 (m, 1H), 2.36 (s, 3H), 1.35 (t, *J* = 7.0 Hz, 3H), 1.18 (t, *J* = 7.1 Hz, 3H). ^{13}C NMR (100 MHz, $CDCl_3$) δ = 139.1 (d, *J* = 2.3 Hz), 131.6 (d, *J* = 3.2 Hz), 129.4, 129.3, 64.1 (t, *J* = 7.0 Hz), 41.6 (d, *J* = 160.2 Hz), 21.3, 16.4 (d, *J* = 5.9 Hz), 16.3 (d, *J* = 5.9 Hz). ^{31}P NMR (162 MHz, $CDCl_3$): δ = 17.25 (s).

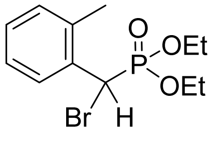

Compound **3c**: purified by TLC plate (petroleum ether : ethyl acetate = 4:1), 23.8 mg, 74% yield, colorless solid. ^{1}H NMR (600 MHz, $CDCl_3$) : δ = 7.89 (d, *J* = 7.5 Hz, 1H), 7.30-7.20 (m, 2H), 7.16

(d, J = 7.4 Hz, 1H), 5.18 (d, J = 13.9 Hz, 1H), 4.29-4.21 (m, 2H), 4.12-4.04 (m, 1H), 3.95-3.84 (m, 1H), 2.41 (s, 3H), 1.36 (t, J = 7.0 Hz, 3H), 1.16 (t, J = 7.0 Hz, 3H). ^{13}C NMR (150 MHz, $CDCl_3$): δ = 136.2, 133.0 (d, J = 2.1 Hz), 130.6 (d, J = 3.8 Hz), 130.4, 128.9 (d, J = 1.6 Hz), 126. 8, 64.2 (d, J = 6.9 Hz), 64.1 (d, J = 6.8 Hz), 37.5 (d, J = 159.4 Hz), 19.6, 16.4 (d, J = 5.9 Hz), 16.2 (d, J = 5.6 Hz). ^{31}P NMR (162 MHz, $CDCl_3$): δ = 17.97 (s). HRMS (ESI) m/z: $[M+H]^+$ calcd for $C_{12}H_{19}BrO_3P^+$ 321.0250, found 321.0247.

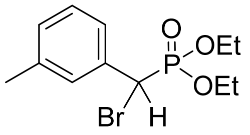

Compound **3d**: purified by TLC plate (petroleum ether : ethyl acetate = 4:1), 23.7 mg, 74% yield, colorless oil. ^{1}H NMR (400 MHz, $CDCl_3$) : δ = 7.37 (d, J = 10.5 Hz, 2H), 7.25 (t, J = 7.6 Hz, 1H), 7.15 (d, J = 7.6 Hz, 1H), 4.84 (d, J = 13.0 Hz, 1H), 4.30-4.19 (m, 2H), 4.12-4.02 (m, 1H), 3.95-3.83 (m, 1H), 2.37 (s, 3H), 1.36 (t, J = 7.1 Hz, 3H), 1.18 (t, J = 7.1 Hz, 3H). ^{13}C NMR (100 MHz, $CDCl_3$): δ = 138.5, 134.4 (d, J = 3.3 Hz), 130.2 (d, J = 6.9 Hz), 129.9 (d, J = 2.1 Hz), 128. 6 (d, J = 1.2 Hz), 126.6 (d, J = 6.6 Hz), 64.1 (d, J = 7.1 Hz), 64.0 (d, J = 6.8 Hz), 41.6 (d, J = 159.3 Hz), 21.4, 16.4 (d, J = 6.0 Hz), 16.2 (d, J = 5.9 Hz). ^{31}P NMR (162 MHz, $CDCl_3$): δ = 17.24 (s). HRMS (ESI) m/z: $[M+Na]^+$ calcd for $C_{12}H_{18}BrNaO_3P^+$ 343.0070, found 343.0072.

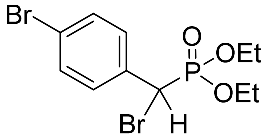

Compound **3e**:[13] purified by TLC plate (petroleum ether : ethyl acetate = 4:1), 36.4 mg, 94% yield, colorless oil. ^{1}H NMR (600 MHz, $CDCl_3$) : δ = 7.49 (d, J = 8.4 Hz, 2H), 7.45 (d, J = 7.3 Hz, 2H), 4.82 (d, J = 13.2 Hz, 1H), 4.26-4.20 (m, 2H), 4.12-4.05 (m, 1H), 3.98-3.90 (m, 1H), 1.35 (t, J = 7.1

Hz, 3H), 1.20 (t, J = 7.1 Hz, 3H). ^{13}C NMR (100 MHz, $CDCl_3$) δ = 133.8 (d, J = 3.6 Hz), 131.9 (d, J = 1.2 Hz), 131.1 (d, J = 6.5 Hz), 123.2 (d, J = 2.8 Hz), 64.4 (d, J = 7.1 Hz), 64.1 (d, J = 6.8 Hz), 40.6 (d, J = 159.4 Hz), 16.4 (d, J = 5.9 Hz), 16.3 (d, J = 5.8 Hz). ^{31}P NMR (162 MHz, $CDCl_3$): δ = 16.53 (s).

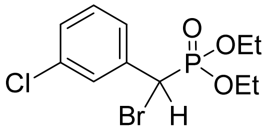

Compound **3f**:[12] purified by TLC plate (petroleum ether : ethyl acetate = 4:1), 30.5 mg, 89% yield, colorless oil. ^{1}H NMR (400 MHz, $CDCl_3$) : δ = 7.66 (s, 1H), 7.57 (d, J = 6.2 Hz, 1H), 7.47-7.35 (m, 2H), 4.92 (d, J = 13.3 Hz, 1H), 4.38-4.28 (m, 2H), 4.27-4.14 (m, 1H), 4.12-4.01 (m, 1H), 1.46 (t, J = 7.0 Hz, 3H), 1.31 (t, J = 7.0 Hz, 3H). ^{13}C NMR (100 MHz, $CDCl_3$) δ = 136.7 (d, J = 3.2 Hz), 134.4 (d, J = 1.3 Hz), 129.9 (d, J = 1.3 Hz), 129.6 (d, J = 6.6 Hz), 129.2 (d, J = 2.2 Hz), 127.7 (d, J = 6.4 Hz), 64.4 (d, J = 7.1 Hz), 64.2 (d, J = 6.9 Hz), 40.4 (d, J = 158.7 Hz), 16.4 (d, J = 5.8 Hz), 16.2 (d, J = 5.7 Hz). ^{31}P NMR (162 MHz, $CDCl_3$): δ = 16.50 (s).

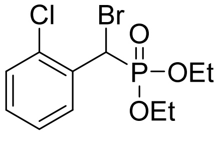

Compound **3g**:[12] purified by TLC plate (petroleum ether : ethyl acetate = 4:1), 28.4 mg, 83% yield, colorless oil. ^{1}H NMR (400 MHz, $CDCl_3$) : δ = 8.00 (d, J = 7.8 Hz, 1H), 7.39-7.26 (m, 3H), 5.55 (d, J = 13.8 Hz, 1H), 4.34-4.25 (m, 2H), 4.12-4.04 (m, 1H), 4.00-3.90 (m, 1H), 1.38 (t, J = 7.1 Hz, 3H), 1.18 (t, J = 7.1 Hz, 3H). ^{13}C NMR (100 MHz, $CDCl_3$) δ = 133.5 (d, J = 10.2 Hz), 132.8, 132.2 (d, J = 3.6 Hz), 130.1 (d, J = 1.9 Hz), 129.5, 127.5 (d, J = 1.9 Hz), 64.4 (d, J = 7.1 Hz), 64.1 (d, J = 6.9 Hz), 36.3 (d, J = 161.5 Hz), 16.4 (d, J = 5.9 Hz), 16.2 (d, J = 5.8 Hz). ^{31}P NMR (162 MHz, $CDCl_3$):

δ = 16.90 (s).

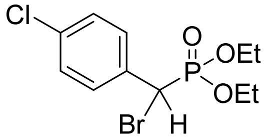

Compound **3h**:[12] purified by TLC plate (petroleum ether : ethyl acetate = 4:1), 31.5 mg, 92% yield, colorless oil. ^{1}H NMR (400 MHz, $CDCl_3$) : δ = 7.51 (d, J = 7.2 Hz, 2H), 7.34 (d, J = 8.4 Hz, 2H), 4.84 (d, J = 13.2 Hz, 1H), 4.31-4.18 (m, 2H), 4.13-4.04 (m, 1H), 3.99-3.88 (m, 1H), 1.35 (t, J = 7.1 Hz, 3H), 1.20 (t, J = 7.1 Hz, 3H). ^{13}C NMR (100 MHz, $CDCl_3$) δ = 134.9 (d, J = 2.6 Hz), 133.3 (d, J = 3.5 Hz), 130.8 (d, J = 6.6 Hz), 128.9 (d, J = 1.2 Hz), 64.3 (d, J = 7.2 Hz), 64.1 (d, J = 7.1 Hz), 40.6 (d, J = 159.6 Hz), 16.4 (d, J = 5.9 Hz), 16.3 (d, J = 5.8 Hz). ^{31}P NMR (162 MHz, $CDCl_3$): δ = 16.66 (s).

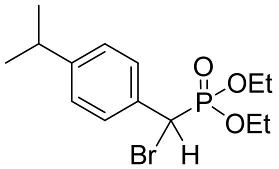

Compound **3i**: purified by TLC plate (petroleum ether : ethyl acetate = 4:1), 20.3 mg, 58% yield, colorless oil. ^{1}H NMR (400 MHz, $CDCl_3$) : δ = 7.49 (d, J = 7.1 Hz, 2H), 7.22 (d, J = 8.1 Hz, 2H), 4.87 (d, J = 12.8 Hz, 1H), 4.29-4.17 (m, 2H), 4.11-4.03 (m, 1H), 3.95-3.85 (m, 1H), 2.98-2.83 (m, 1H), 1.35 (t, J = 7.0 Hz, 3H), 1.25 (d, J = 6.9 Hz, 6H), 1.17 (t, J = 7.0 Hz, 3H). ^{13}C NMR (100 MHz, $CDCl_3$): δ = 149.9, 131. 8 (d, J = 3.2 Hz), 129.5 (d, J = 6.6 Hz), 126.8, 64.1 (d, J = 7.5 Hz), 64.0 (d, J = 7.1 Hz), 41.6 (d, J = 160.1 Hz), 33.9, 23.8, 16.4 (d, J = 5.9 Hz), 16.2 (d, J = 5.9 Hz). ^{31}P NMR (162 MHz, $CDCl_3$): δ = 17.30 (s). HRMS (ESI) m/z: $[M+Na]^+$ calcd for $C_{14}H_{22}BrNaO_3P^+$ 371.0383, found 371.0389.

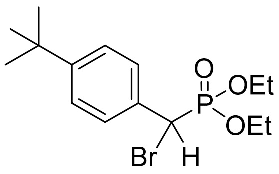

Compound **3j**: purified by TLC plate (petroleum ether : ethyl acetate = 4:1), 27.7 mg, 76% yield, colorless oil. ^{1}H NMR (400 MHz, $CDCl_3$) : δ = 7.49 (d, *J* = 8.3 Hz, 2H), 7.37 (d, *J* = 8.3 Hz, 2H), 4.88 (d, *J* = 12.8 Hz, 1H), 4.28-4.19 (m, 2H), 4.12-4.02 (m, 1H), 3.98-3.80 (m, 1H), 1.35 (t, *J* = 7.0 Hz, 3H), 1.32 (s, 9H), 1.16 (t, *J* = 7.1 Hz, 3H). ^{13}C NMR (100 MHz, $CDCl_3$): δ = 152.2 (d, *J* = 2.3 Hz), 131.4 (d, *J* = 3.4 Hz), 129.2 (d, *J* = 6.6 Hz), 125.7 (d, *J* = 1.1 Hz), 64.1 (d, *J* = 6.9 Hz), 64.0 (d, *J* = 7.1 Hz), 41.5 (d, *J* = 160.2 Hz), 34.7, 31.2, 16.4 (d, *J* = 5.9 Hz), 16.2 (d, *J* = 5.7 Hz). ^{31}P NMR (162 MHz, $CDCl_3$): δ = 17.30 (s). HRMS (ESI) m/z: $[M+Na]^+$ calcd for $C_{15}H_{24}BrNaO_3P^+$ 385.0539, found 385.0541.

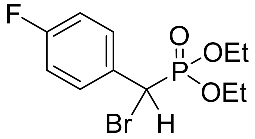

Compound **3l**:[13] purified by TLC plate (petroleum ether : ethyl acetate = 4:1), 30.2 mg, 93% yield, colorless oil. ^{1}H NMR (600 MHz, $CDCl_3$) : δ = 7.61-7.53 (m, 2H), 7.05 (t, *J* = 8.6 Hz, 2H), 4.86 (d, *J* = 13.2 Hz, 1H), 4.28-4.21 (m, 2H), 4.12-4.02 (m, 1H), 3.97-3.85 (m, 1H), 1.35 (t, *J* = 7.1 Hz, 3H), 1.18 (t, *J* = 7.1 Hz, 3H). ^{19}F NMR (376 MHz, $CDCl_3$) δ = -111.94 (d, *J* = 3.3 Hz, 1F). ^{13}C NMR (100 MHz, $CDCl_3$) δ = 162.9 (d, *J* = 249.2 Hz), 131.4 (dd, *J* = 8.5, 6.7 Hz), 130.6 (t, *J* = 3.2 Hz), 115.7 (d, *J* = 21.8 Hz), 64.3 (d, *J* = 7.1 Hz), 64.1 (d, *J* = 7.0 Hz), 40.6 (d, *J* = 160.6 Hz), 16.4 (d, *J* = 5.9 Hz), 16.2 (d, *J* = 5.7 Hz). ^{31}P NMR (162 MHz, $CDCl_3$): δ = 16.89 (d, *J* = 3.3 Hz, 1P).

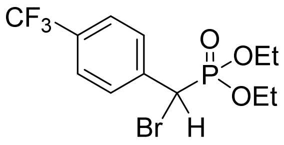

Compound **3m**: purified by TLC plate (petroleum ether : ethyl acetate = 4:1), 34.0 mg, 91% yield, colorless oil. ^{1}H NMR (600 MHz, $CDCl_3$) : δ = 7.70 (d, *J* = 7.8 Hz, 2H), 7.63 (d, *J* = 8.3 Hz, 2H), 4.90 (d, *J* = 13.5 Hz, 1H), 4.28-4.22 (m, 2H), 4.14-4.08 (m, 1H), 4.02-3.93 (m, 1H), 1.36 (t, *J* = 7.1 Hz, 3H), 1.21 (t, *J* = 7.1 Hz, 3H). ^{13}C NMR (100 MHz, $CDCl_3$): δ = 138.8, 130.9 (d, *J* = 32.9 Hz), 129.9 (d, *J* = 6.2 Hz), 125.8-125.4 (m), 123.8 (d, *J* = 272.5 Hz), 64.5 (d, *J* = 7.2 Hz), 64.2 (d, *J* = 6.8 Hz), 40.3 (d, *J* = 158.2 Hz), 16.4 (d, *J* = 5.8 Hz), 16.2 (d, *J* = 5.7 Hz). ^{19}F NMR (376 MHz, $CDCl_3$): δ = -62.8 (s, 3F). ^{31}P NMR (162 MHz, $CDCl_3$): δ = 16.38 (s). HRMS (ESI) m/z: $[M+Na]^+$ calcd for $C_{12}H_{15}BrF_3NaO_3P^+$ 396.9787, found 396.9787.

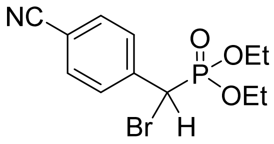

Compound **3n**:[14] purified by TLC plate (petroleum ether : ethyl acetate = 4:1), 28.8 mg, 87% yield, colorless oil. ^{1}H NMR (600 MHz, $CDCl_3$): δ = 7.73-7.56 (m, 5H), 4.87 (d, *J* = 13.7 Hz, 1H), 4.29-4.19 (m, 2H), 4.13-4.06 (m, 1H), 4.03-3.90 (m, 1H), 1.34 (t, *J* = 7.0 Hz, 3H), 1.20 (t, *J* = 7.0 Hz, 3H). ^{13}C NMR (100 MHz, $CDCl_3$) δ = 140.1 (d, *J* = 3.7 Hz), 132.4 (d, *J* = 1.3 Hz), 130.3 (d, *J* = 6.2 Hz), 118.2, 112.8, 64.6 (d, *J* = 7.0 Hz), 64.2 (d, *J* = 7.1 Hz), 40.2 (d, *J* = 157.5 Hz), 16.4 (d, *J* = 5.8 Hz), 16.3 (d, *J* = 5.7 Hz). ^{31}P NMR (162 MHz, $CDCl_3$): δ = 15.97 (s).

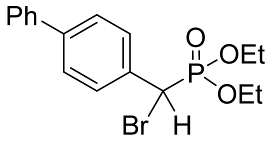

Compound **3o**: purified by TLC plate (petroleum ether : ethyl acetate = 4:1), 27.4 mg, 72% yield,

colorless oil. ^{1}H NMR (600 MHz, $CDCl_3$) : δ = 7.70-7.64 (m, 2H), 7.63-7.60 (m, 4H), 7.47 (t, *J* = 7.7 Hz, 2H), 7.38 (t, *J* = 7.4 Hz, 1H), 4.94 (d, *J* = 13.0 Hz, 1H), 4.31-4.24 (m, 2H), 4.17-4.06 (m, 1H), 4.01-3.91 (m, 1H), 1.38 (t, *J* = 7.1 Hz, 3H), 1.21 (t, *J* = 7.1 Hz, 3H). ^{13}C NMR (100 MHz, $CDCl_3$): δ = 141.9 (d, *J* = 2.2 Hz), 140.2, 133.6 (d, *J* = 3.2 Hz), 129.9 (d, *J* = 6.7 Hz), 128.9, 127.7, 127.4 (d, *J* = 1.1 Hz), 127.1, 64.3 (d, *J* = 7.1 Hz), 64.1 (d, *J* = 7.0 Hz), 41.3 (d, *J* = 159.7 Hz), 16.5 (d, *J* = 5.9 Hz), 16.3 (d, *J* = 5.7 Hz). ^{31}P NMR (162 MHz, $CDCl_3$): δ = 17.06 (s, 1P). HRMS (ESI) m/z: $[M+Na]^+$ calcd for $C_{17}H_{20}BrNaO_3P^+$ 405.0226, found 405.0226.

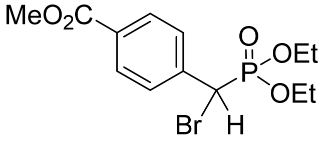

Compound **3p**: purified by TLC plate (petroleum ether : ethyl acetate = 4:1), 25.4 mg, 70% yield, colorless oil. ^{1}H NMR (400 MHz, $CDCl_3$): δ = 8.03 (d, *J* = 8.3 Hz, 2H), 7.64 (d, *J* = 8.2 Hz, 2H), 4.90 (d, *J* = 13.5 Hz, 1H), 4.28-4.19 (m, 2H), 4.12-4.03 (m, 1H), 3.99-3.87 (m, 4H), 1.35 (t, *J* = 7.1 Hz, 3H), 1.18 (t, *J* = 7.1 Hz, 3H). ^{13}C NMR (100 MHz, $CDCl_3$): δ = 166. 5, 139.6 (d, *J* = 3.6 Hz), 130.6 (d, *J* = 2.3 Hz), 129.9 (d, *J* = 1.3 Hz), 129.6 (d, *J* = 6.2 Hz), 64.4 (d, *J* = 7.1 Hz), 64.2 (d, *J* = 6.8 Hz), 52.3, 40.6 (d, *J* = 157.8 Hz), 16.4 (d, *J* = 5.9 Hz), 16.2 (d, *J* = 5.8 Hz). ^{31}P NMR (162 MHz, $CDCl_3$): δ = 16.49 (s). HRMS (ESI) m/z: $[M+Na]^+$ calcd for $C_{13}H_{18}BrNaO_5P^+$ 386.9968, found 386.9965.

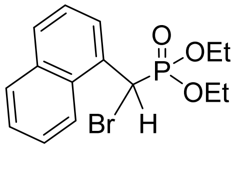

Compound **3q**: purified by TLC plate (petroleum ether : ethyl acetate = 4:1), 24.7 mg, 69% yield, colorless oil. ^{1}H NMR (400 MHz, $CDCl_3$) : δ = 8.16 (d, *J* = 75.3 Hz, 2H), 7.95-7.83 (m, 2H), 7.62

(t, J = 7.5 Hz, 1H), 7.54 (t, J = 7.3 Hz, 2H), 5.96-5.68 (m, 1H), 4.37-4.26 (m, 2H), 4.12-3.99 (m, 1H), 3.91-3.68 (m, 1H), 1.37 (t, J = 7.1 Hz, 3H), 1.04 (t, J = 6.9 Hz, 3H). ^{13}C NMR (100 MHz, $CDCl_3$): δ = 133.8, 130.8, 130.4, 129.8, 129.3 (d, J = 5.5 Hz), 129.1, 126.9, 126.0, 125.6, 122.4, 64.3 (d, J = 6.8 Hz), 64.1 (d, J = 6.8 Hz), 16.5 (d, J = 5.9 Hz), 16.1 (d, J = 5.7 Hz). ^{31}P NMR (162 MHz, $CDCl_3$): δ = 17.80 (s). HRMS (ESI) m/z: $[M+Na]^+$ calcd for $C_{15}H_{18}BrNaO_3P^+$ 379.0070, found 379.0069.

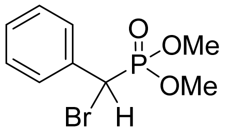

Compound **3r**:[15] purified by TLC plate (petroleum ether : ethyl acetate = 4:1), 24.1 mg, 86% yield, colorless oil. ^{1}H NMR (400 MHz, $CDCl_3$) : δ = 7.58 (d, J = 7.6 Hz, 2H), 7.40-7.33 (m, 3H), 4.90 (d, J = 13.1 Hz, 1H), 3.87 (d, J = 10.8 Hz, 3H), 3.63 (d, J = 10.7 Hz, 3H). ^{13}C NMR (100 MHz, $CDCl_3$) δ = 134.3 (d, J = 3.4 Hz), 129.5 (d, J = 6.6 Hz), 129.2 (d, J = 2.3 Hz), 128.8, 54.8 (d, J = 7.2 Hz), 54.6 (d, J = 7.0 Hz), 40.9 (d, J = 159.9 Hz). ^{31}P NMR (162 MHz, $CDCl_3$): δ = 19.48 (s).

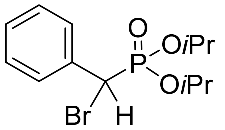

Compound **3s**: purified by TLC plate (petroleum ether : ethyl acetate = 4:1), 21.6 mg, 64% yield, colorless oil. ^{1}H NMR (400 MHz, $CDCl_3$) : δ = 7.58 (d, J = 7.3 Hz, 2H), 7.40-7.29 (m, 3H), 4.90-4.77 (m, 1H), 4.82 (d, J = 13.2 Hz, 1H), 4.64-4.48 (m, 1H), 1.37 (d, J = 6.2 Hz, 3H), 1.34 (d, J = 6.2 Hz, 3H), 1.28 (d, J = 6.2 Hz, 3H), 0.97 (d, J = 6.2 Hz, 3H). ^{13}C NMR (100 MHz, $CDCl_3$): δ = 134.9 (d, J = 3.3 Hz), 129.6 (d, J = 6.6 Hz), 128.9 (d, J = 2.1 Hz), 128.6 (d, J = 1.2 Hz), 72.9 (d, J = 7.0 Hz), 72.7 (d, J = 7.3 Hz), 42.2 (d, J = 160.8 Hz), 24.3 (d, J = 2.6 Hz), 24.2 (d, J = 3.1 Hz),

23.7 (d, $J = 6.0$ Hz), 23.1 (d, $J = 5.9$ Hz). ^{31}P NMR (162 MHz, $CDCl_3$): δ = 15.54 (s). HRMS (ESI) m/z: $[M+Na]^+$ calcd for $C_{13}H_{20}BrNaO_3P^+$ 357.0226, found 357.0221.

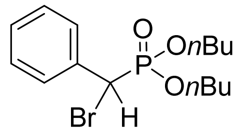

Compound **3t**: purified by TLC plate (petroleum ether : ethyl acetate = 4:1), 20.8 mg, 57% yield, colorless oil. ^{1}H NMR (400 MHz, $CDCl_3$) : δ = 7.57 (d, $J = 7.4$ Hz, 2H), 7.38-7.32 (m, 3H), 4.88 (d, $J = 13.0$ Hz, 1H), 4.25-4.07 (m, 2H), 4.06-3.95 (m, 1H), 3.85-3.75 (m, 1H), 1.71-1.62 (m, 2H), 1.53-1.44 (m, 2H), 1.43-1.33 (m, 2H), 1.28-1.21 (m, 2H), 0.94 (t, $J = 7.4$ Hz, 3H), 0.85 (t, $J = 7.4$ Hz, 3H). ^{13}C NMR (100 MHz, $CDCl_3$): δ = 134.7 (d, $J = 3.1$ Hz), 129.5 (d, $J = 6.6$ Hz), 128.9 (d, $J = 2.1$ Hz), 128. 7 (d, $J = 1.1$ Hz), 67. 8 (d, $J = 5.2$ Hz), 67.7 (d, $J = 5.0$ Hz), 41.5 (d, $J = 159.4$ Hz), 32.5 (d, $J = 5.9$ Hz), 32.4 (d, $J = 5.9$ Hz), 18.6 (d, $J = 11.8$ Hz), 13.5 (d, $J = 7.7$ Hz). ^{31}P NMR (162 MHz, $CDCl_3$): δ = 17.06 (s). HRMS (ESI) m/z: $[M+H]^+$ calcd for $C_{15}H_{25}BrO_3P^+$ 363.0720, found 363.0724.

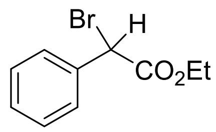

Compound **5**:[16] purified by TLC plate (petroleum ether : ethyl acetate = 4:1), 13.8 mg, 57% yield, colorless oil. ^{1}H NMR (400 MHz, $CDCl_3$) : δ = 7.61-7.54 (m, 2H), 7.43-7.34 (m, 3H), 5.37 (s, 1H), 4.32-4.20 (m, 2H), 1.31 (t, $J = 7.1$ Hz, 3H). ^{13}C NMR (100 MHz, $CDCl_3$) δ = 168.3, 135.9, 129.3, 128.8, 128.7, 62.5, 46.9, 13.9.

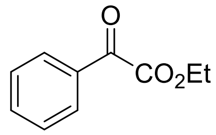

Compound **6**:[17] purified by TLC plate (petroleum ether : ethyl acetate = 4:1), <5.5 mg, <10% yield, colorless oil. ^{1}H NMR (400 MHz, $CDCl_3$) : δ = 8.07-8.01 (m, 2H), 7.69 (t, *J* = 7.4 Hz, 1H), 7.54 (t, *J* = 7.8 Hz, 2H), 4.48 (q, *J* = 7.1 Hz, 2H), 1.45 (t, *J* = 7.1 Hz, 3H). ^{13}C NMR (100 MHz, $CDCl_3$) δ = 186.5, 163.9, 134.9, 132.5, 130.0, 128.9, 62.4, 14.1.

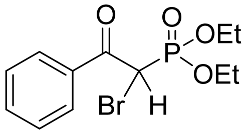

Compound **7**:[18] purified by TLC plate (petroleum ether : ethyl acetate = 4:1), 7.6 mg, 23% yield, colorless oil. ^{1}H NMR (400 MHz, $CDCl_3$) : δ = 8.07-8.01 (m, 2H), 7.64 (t, *J* = 7.4 Hz, 1H), 7.52 (t, *J* = 7.9 Hz, 2H), 5.36 (d, *J* = 13.9 Hz, 1H), 4.37-4.23 (m, 4H), 1.37 (t, *J* = 7.1 Hz, 3H), 1.32 (t, *J* = 7.0 Hz, 3H). ^{13}C NMR (100 MHz, $CDCl_3$) δ = 189.7, 134.6 (d, *J* = 3.8 Hz), 134.2, 129.3, 128.8, 64.6 (t, *J* = 6.3 Hz), 38.4 (d, *J* = 146.2 Hz), 16.3 (t, *J* = 6.2 Hz). ^{31}P NMR (162 MHz, $CDCl_3$): δ = 13.41 (s, 1P).

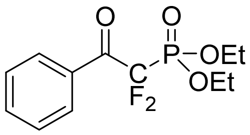

Compound **8**:[19] purified by TLC plate (petroleum ether : ethyl acetate = 4:1), 8.3 mg, 28% yield, colorless oil. ^{1}H NMR (400 MHz, $CDCl_3$) : δ = 8.17 (d, *J* = 8.1 Hz, 2H), 7.67 (t, *J* = 7.4 Hz, 1H), 7.53 (t, *J* = 7.8 Hz, 2H), 4.42-4.32 (m, 4H), 1.40 (t, *J* = 7.1 Hz, 6H). ^{13}C NMR (100 MHz, $CDCl_3$) δ = 188.1, 134.7, 132.0, 130.4 (t, *J* = 2.9 Hz), 128.7, 117.4 (d, *J* = 274.7 Hz), 65.4 (d, *J* = 6.7 Hz), 16.3 (d, *J* = 5.8 Hz). ^{19}F NMR (376 MHz, $CDCl_3$) δ = -110.02 (d, *J* = 95.7 Hz). ^{31}P NMR (162 MHz, $CDCl_3$): δ = 3.52 (t, *J* = 95.5 Hz, 1P).

References

1. Keipour, H.; Jalba, A.; Delage-Laurin, L.; Ollevier, T. *J. Org. Chem.* **2017**, *82*, 3000–3010.

2. Jászay, M.; Zs. Pham, S. T.; Gönczi, K.; Petneházy, I.; Tőke, L. *Synthetic. Commun.* **2010**, *40*, 1574–1579.

3. H. Mei, J. Liu, R. Pajkert, L. Wang, G. V. Röschenthaler, J. L. Han, *Org. Chem. Front.* **2021**, *8*, 767–772.

4. a) W. Miao, Y. Gao, X. Li, Y. Gao, G. Tang, Y. Zhao, *Adv. Synth. Catal.* **2012**, *354*, 2659 – 2664; b) N. A. Zinovyev, I. P. Beletskaya, I. D. Titanyuk, *Synthesis.* **2024**, *56*, 2687–2694; c) P. Beletskaya, I. D. Titanyuk, *J. Org. Chem.* **2022**, *87*, 2748–2757.

5. Gaussian 16, Revision B.01, Frisch, M. J.; Trucks, G. W.; Schlegel, H. B.; Scuseria, G. E.; Robb, M. A.; Cheeseman, J. R.; Scalmani, G.; Barone, V.; Petersson, G. A.; Nakatsuji, H.; Li, X.; Caricato, M.; Marenich, A. V.; Bloino, J.; Janesko, B. G.; Gomperts, R.; Mennucci, B.; Hratchian, H. P.; Ortiz, J. V.; Izmaylov, A. F.; Sonnenberg, J. L.; Williams-Young, D.; Ding, F.; Lipparini, F.; Egidi, F.; Goings, J.; Peng, B.; Petrone, A.; Henderson, T.; Ranasinghe, D.; Zakrzewski, V. G.; Gao, J.; Rega, N.; Zheng, G.; Liang, W.; Hada, M.; Ehara, M.; Toyota, K.; Fukuda, R.; Hasegawa, J.; Ishida, M.; Nakajima, T.; Honda, Y.; Kitao, O.; Nakai, H.; Vreven, T.; Throssell, K.; Montgomery, J. A., Jr.; Peralta, J. E.; Ogliaro, F.; Bearpark, M. J.; Heyd, J. J.; Brothers, E. N.; Kudin, K. N.; Staroverov, V. N.; Keith, T. A.; Kobayashi, R.; Normand, J.; Raghavachari, K.; Rendell, A. P.; Burant, J. C.; Iyengar, S. S.; Tomasi, J.; Cossi, M.; Millam, J. M.; Klene, M.; Adamo, C.; Cammi, R.; Ochterski, J. W.; Martin, R. L.; Morokuma, K.; Farkas, O.; Foresman, J. B.; Fox, D. J. Gaussian, Inc., Wallingford CT, 2016.

6. Becke, A. D. *J. Chem. Phys.* **1993**, *98*, 5648–5652.

7. Grimme, S.; Ehrlich, S.; Goerigk, L. *J. Comput. Chem*. **2011**, *32*, 1456–1465.

8. a) Raghavachari, K.; Binkley, J. S.; Seeger, R.; Pople, J. A. *J. Chem. Phys*. **1980**, *72*, 650-654; b) McLean, A. D.; Chandler, G. S. *J. Chem. Phys*. **1980**, *72*, 5639-5648.

9. Marenich, A. V.; Cramer, C. J.; Truhlar, D. G. *J. Phys. Chem. B* **2009**, *113*, 6378–6396.

10. Gonzalez, C.; Schlegel, H. B. *J. Phys. Chem*. **1990**, *94*, 5523–5527

11. Legault, C. Y. CYLview20. Canada Université de Sherbrooke 2020, http://www.cylview.org

12. H. Firouzabadi, N. Iranpoor, S. Sobhani, *Tetrahedron*. **2004**, *60*, 203-210.

13. F. Eymery, B. Lorga, P. Savignac, Tetrahedron. **1999**, 55, 2671-2686.

14. J.-P. Fu, Y.-H. He, J. Zhong, Y. Yang, X. Deng, Z. Guan, J. Fluor. Chem. **2011**, *132*, 636-640.

15. N.S. Tulsi, A.M. Downey, C.W. Cairo, *Bioorg. Med. Chem.* **2010**, *18*, 8679-8686.

16. Lopchuk, J. M.; Hughes, R. P.; Gribble, G. W, *Org. Lett*. **2013**, *15*, 5218– 5221.

17. K. Matsunaka, T. Iwahama, S. Sakaguchi, Y. Ishii, *Tetrahedron Lett*. **1999**, *40*, 2165-2168.

18. Lin, L.; Romano, C.; Mazet, C, *J. Am. Chem. Soc*. **2016**, *138*, 10344– 10350.

19. Radwan-Olszewska, K.; Palacios, F.; Kafarski, P, *J. Org. Chem*. **2011**, *76*, 1170– 1173.

11. ^{1}H, ^{13}C, ^{19}F and ^{31}P NMR spectra for compound 3, 5, 6, 7 and 8

^{1}H NMR (400 MHz, CDCl3) of **3a**:

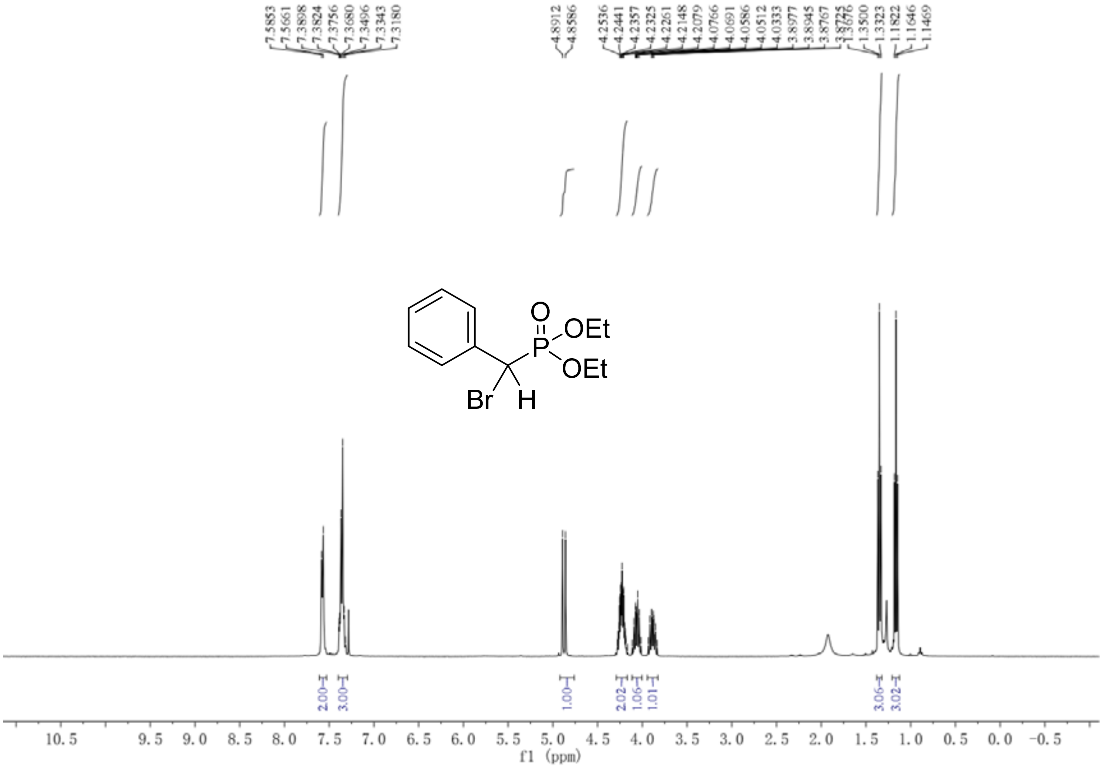

^{13}C NMR (100 MHz, $CDCl_3$) of **3a**:

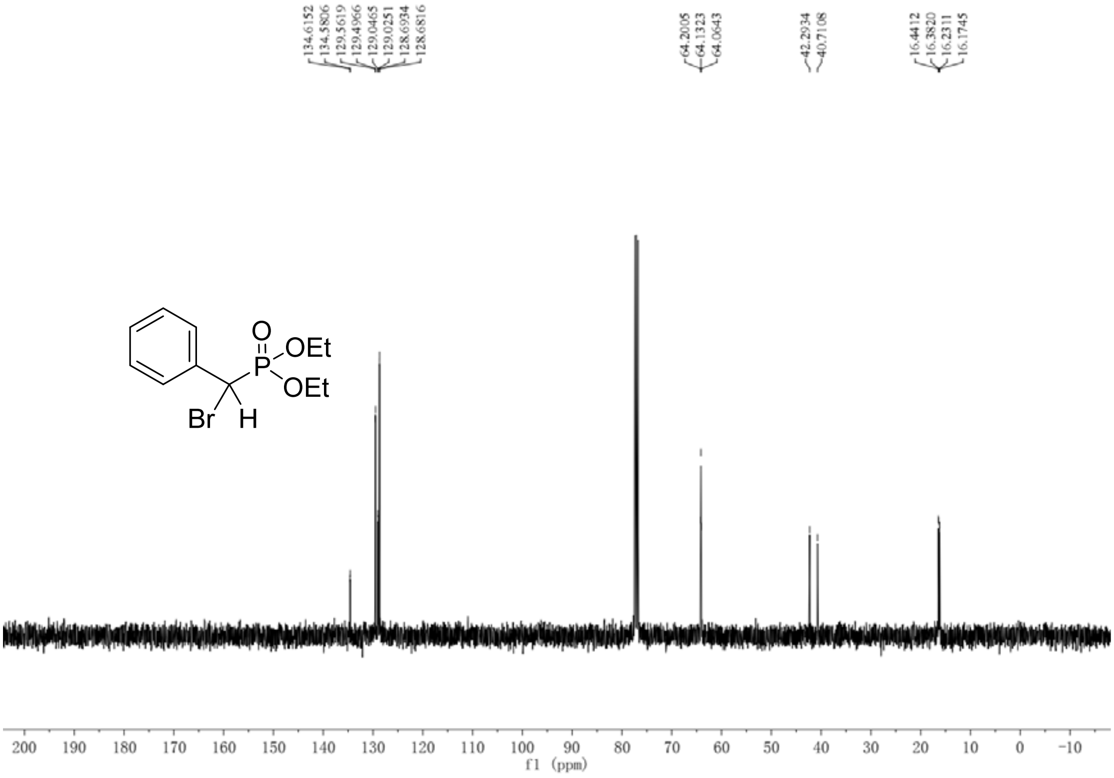

^{31}P NMR (162 MHz, $CDCl_3$) of **3a**:

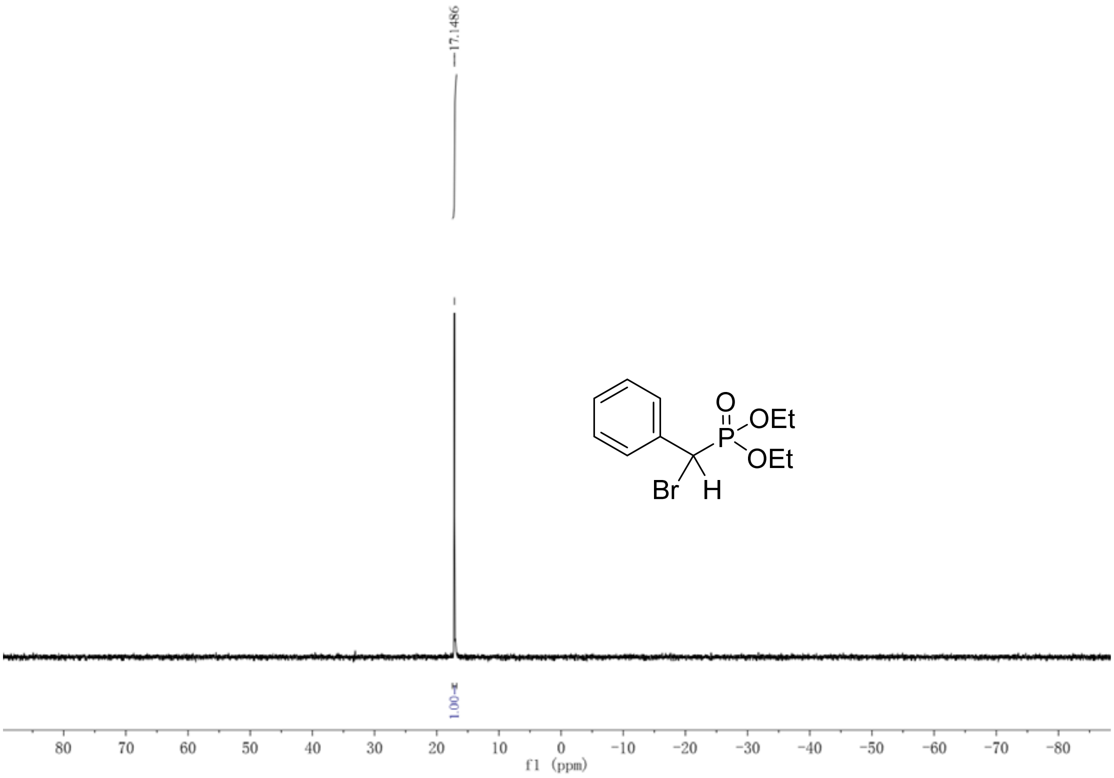

^{1}H NMR (400 MHz, CDCl3) of **3b**:

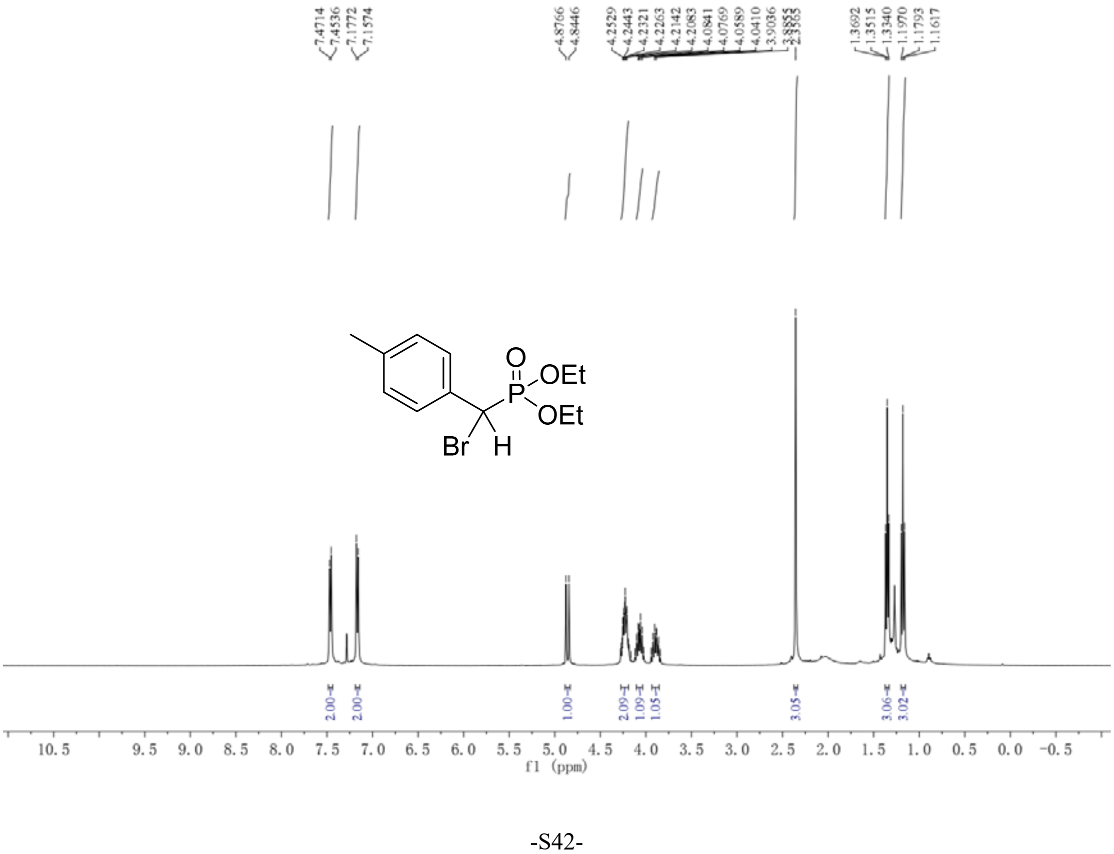

^{13}C NMR (100 MHz, $CDCl_3$) of **3b**:

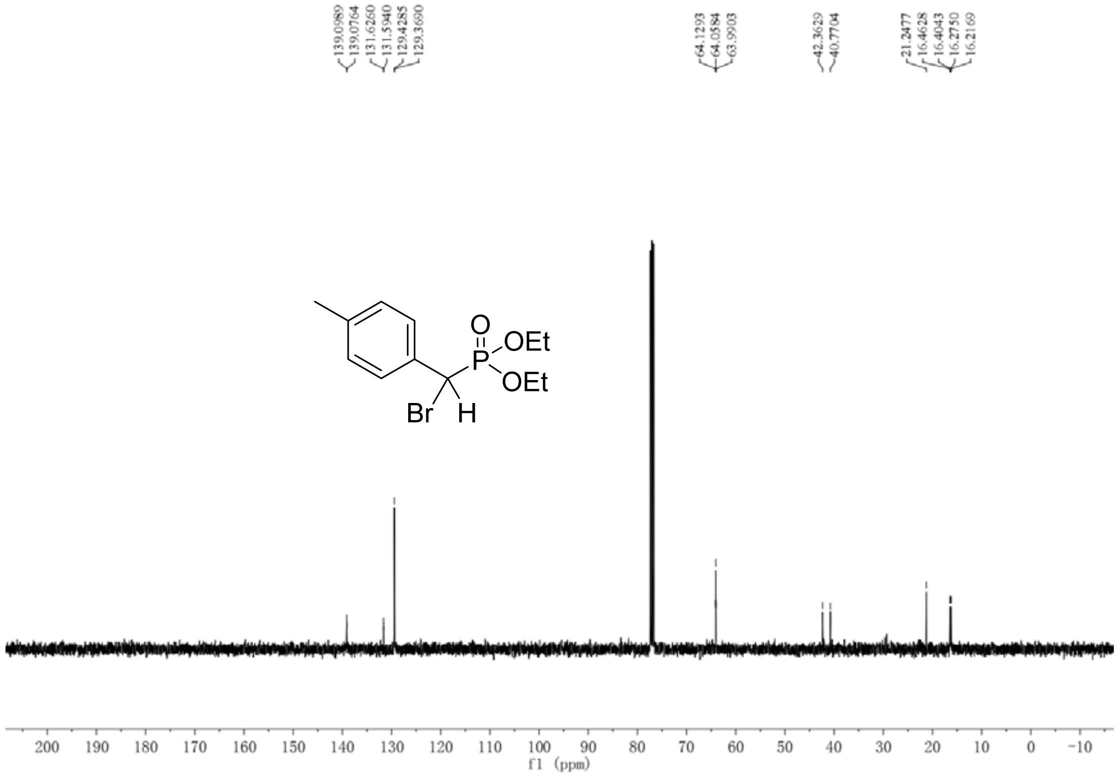

^{31}P NMR (162 MHz, CDCl3) of **3b**:

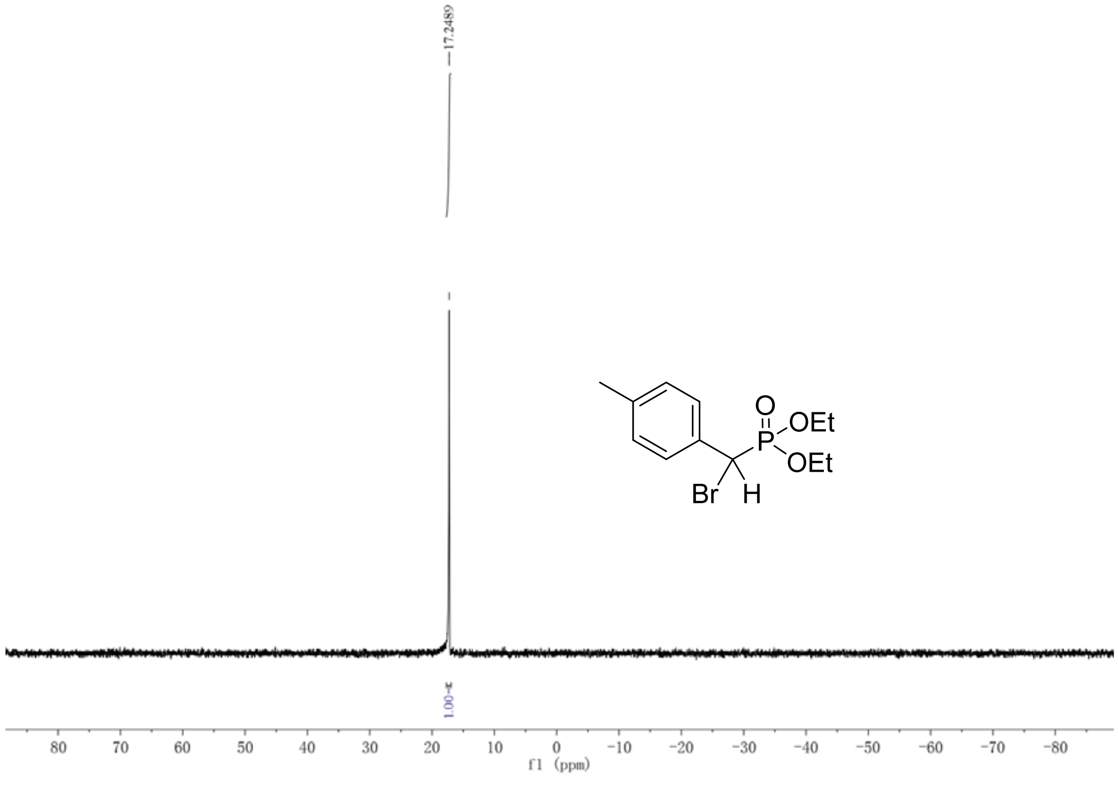

^{1}H NMR (600 MHz, CDCl3) of **3c**:

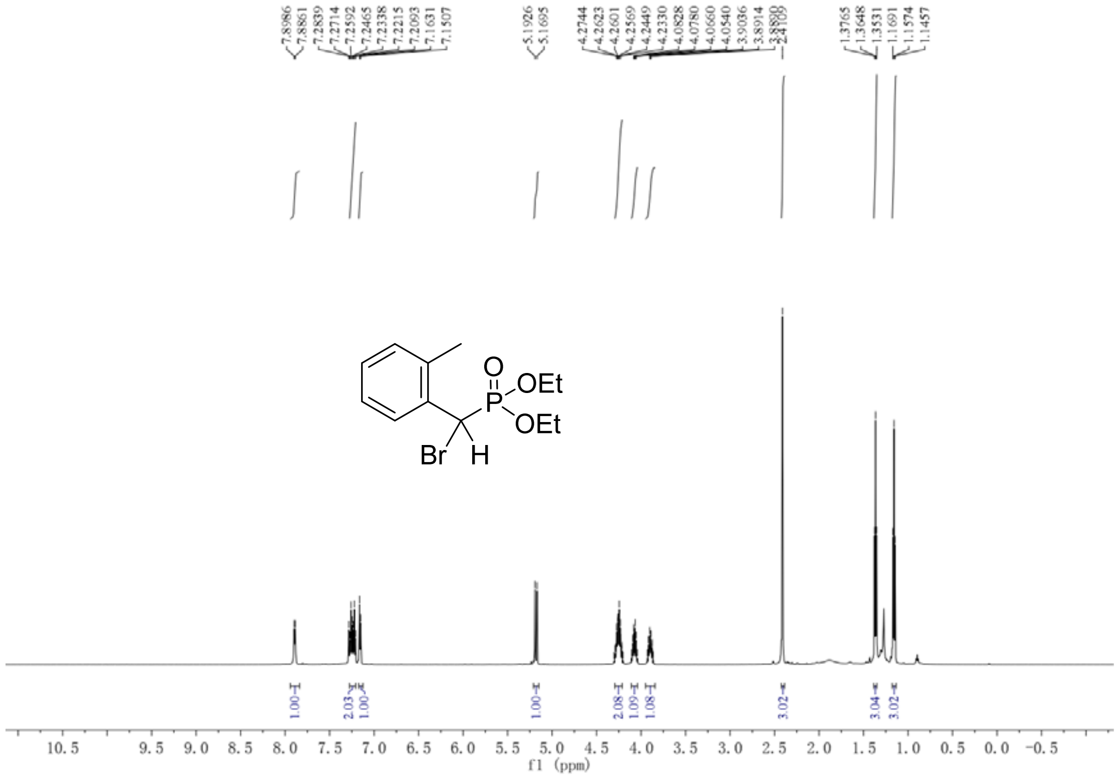

^{13}C NMR (150 MHz, $CDCl_3$) of **3c**:

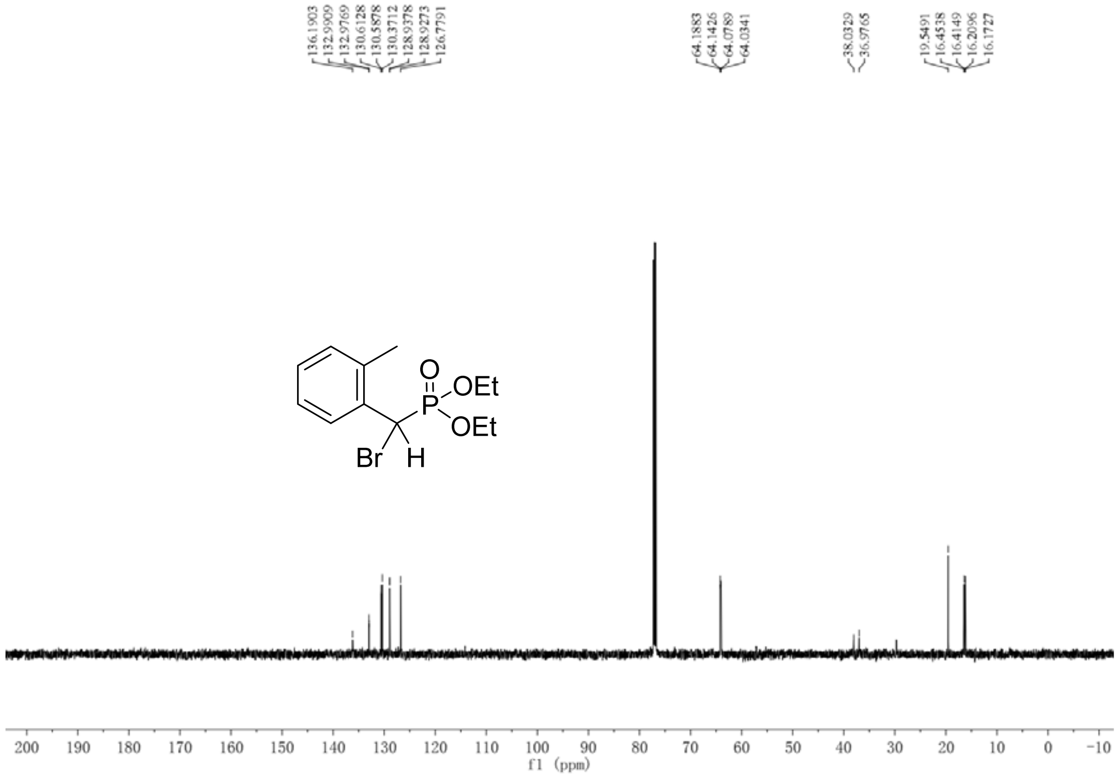

^{31}P NMR (162 MHz, $CDCl_3$) of **3c**:

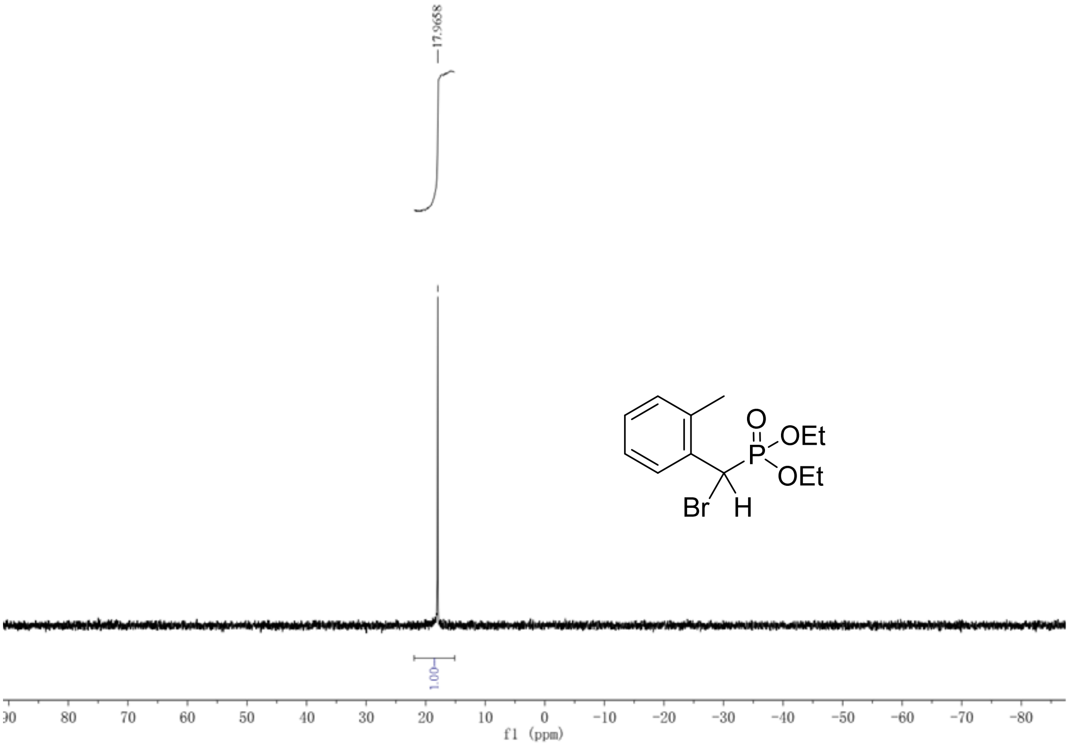

^{1}H NMR (400 MHz, CDCl3) of **3d**:

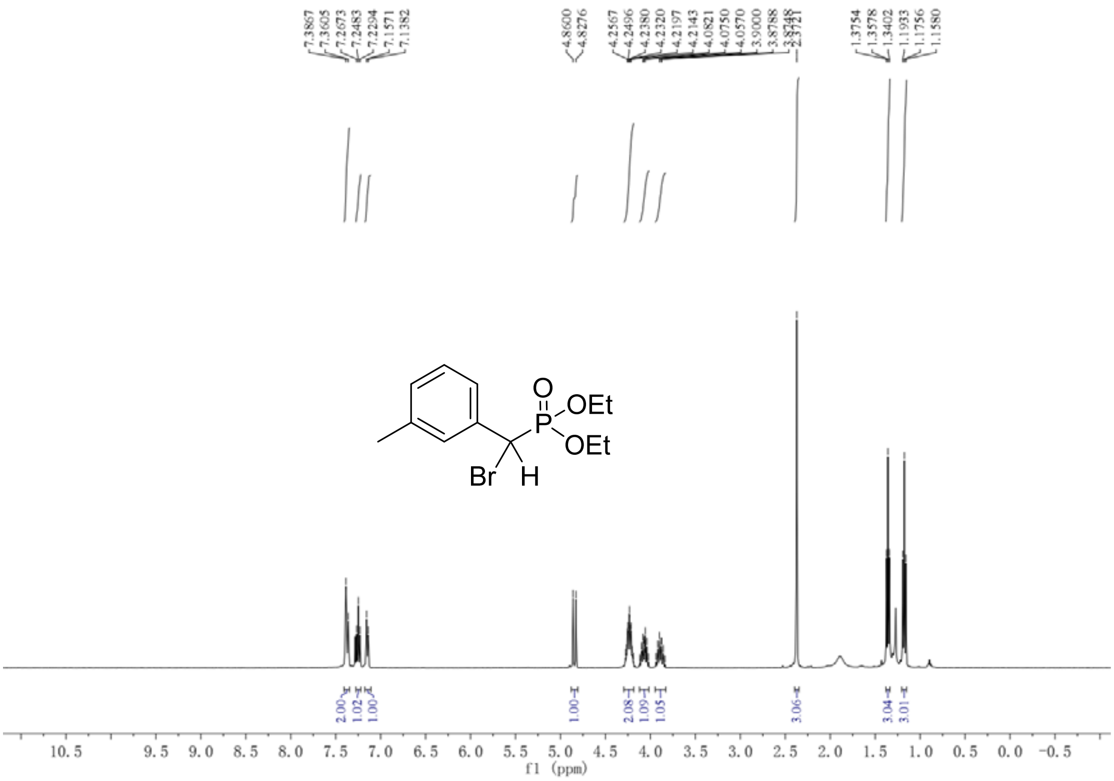

^{13}C NMR (100 MHz, $CDCl_3$) of **3d**:

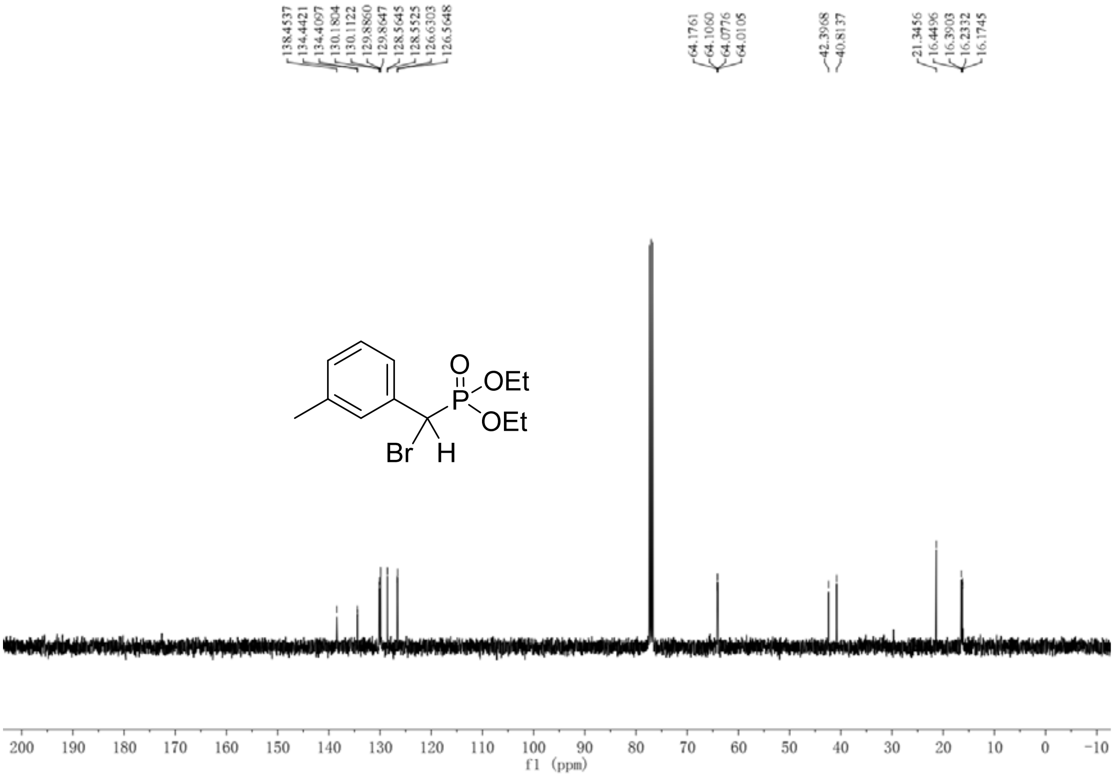

^{31}P NMR (162 MHz, $CDCl_3$) of **3d**:

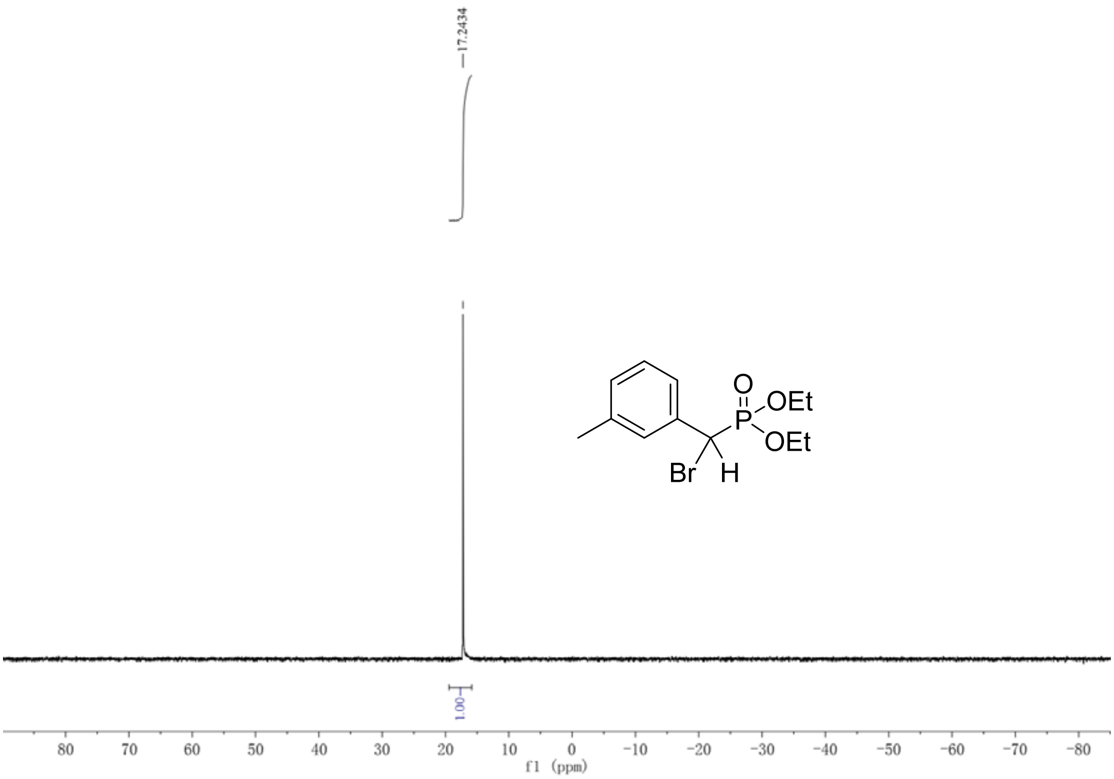

^{1}H NMR (600 MHz, CDCl3) of **3e**:

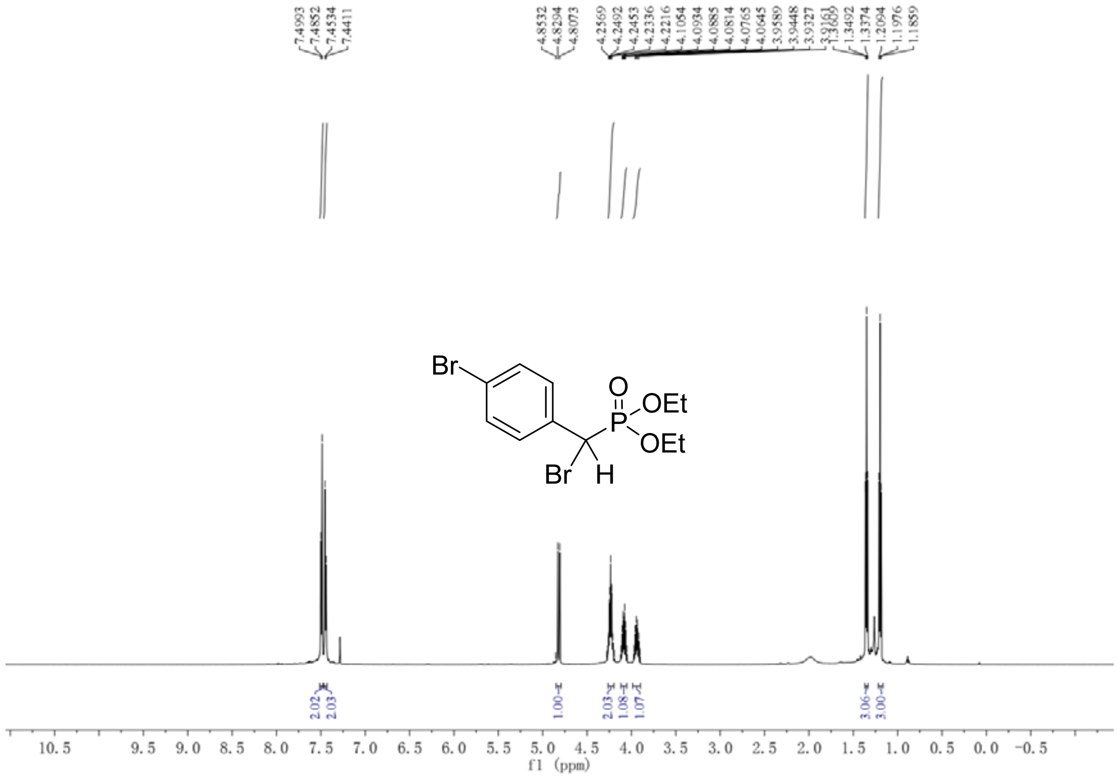

^{13}C NMR (100 MHz, $CDCl_3$) of **3e**:

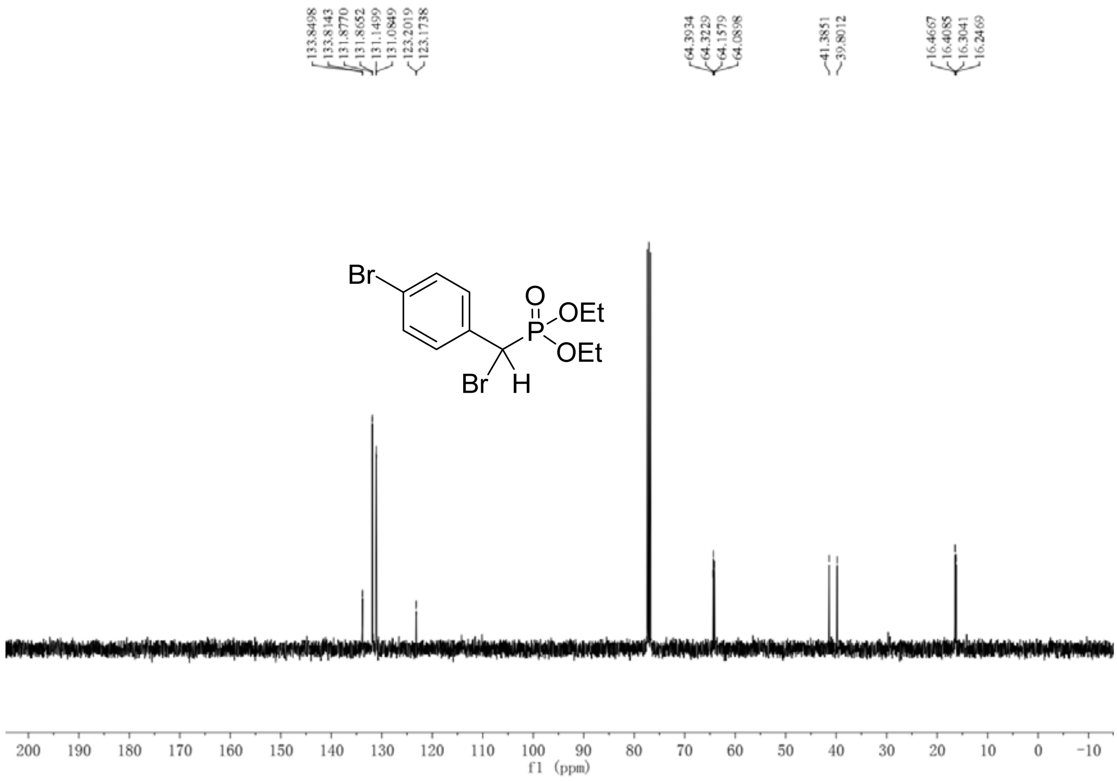

^{31}P NMR (162 MHz, $CDCl_3$) of **3e**:

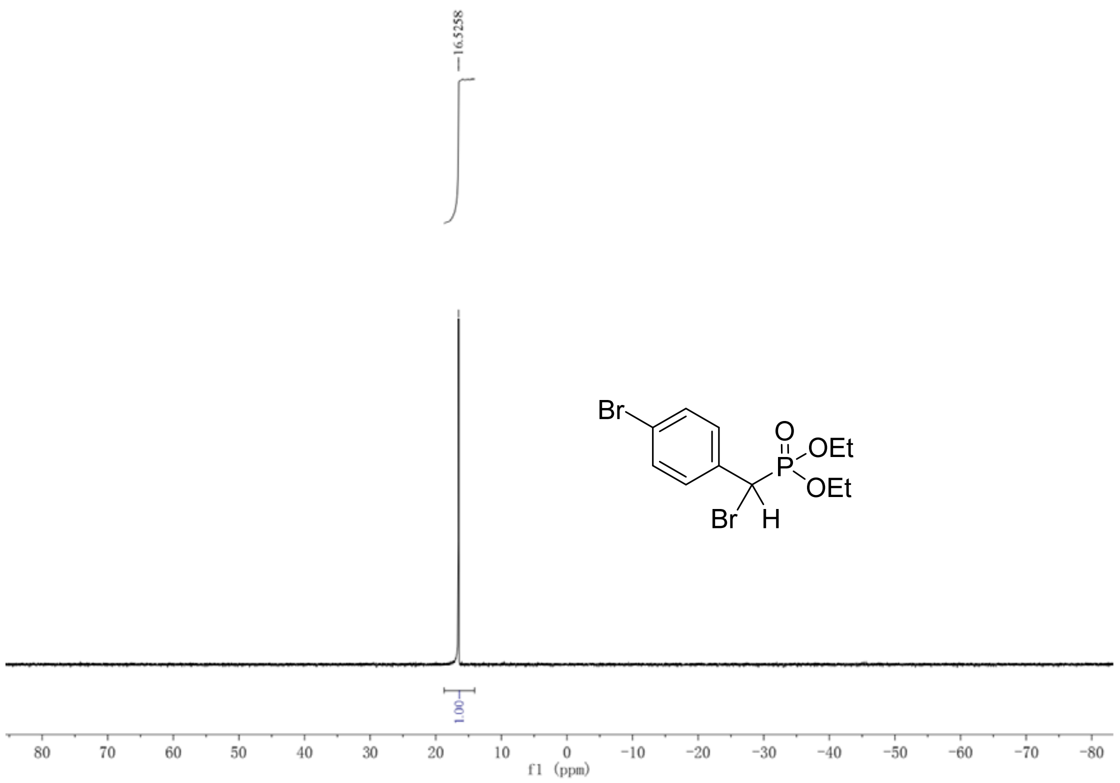

^{1}H NMR (400 MHz, CDCl3) of **3f**:

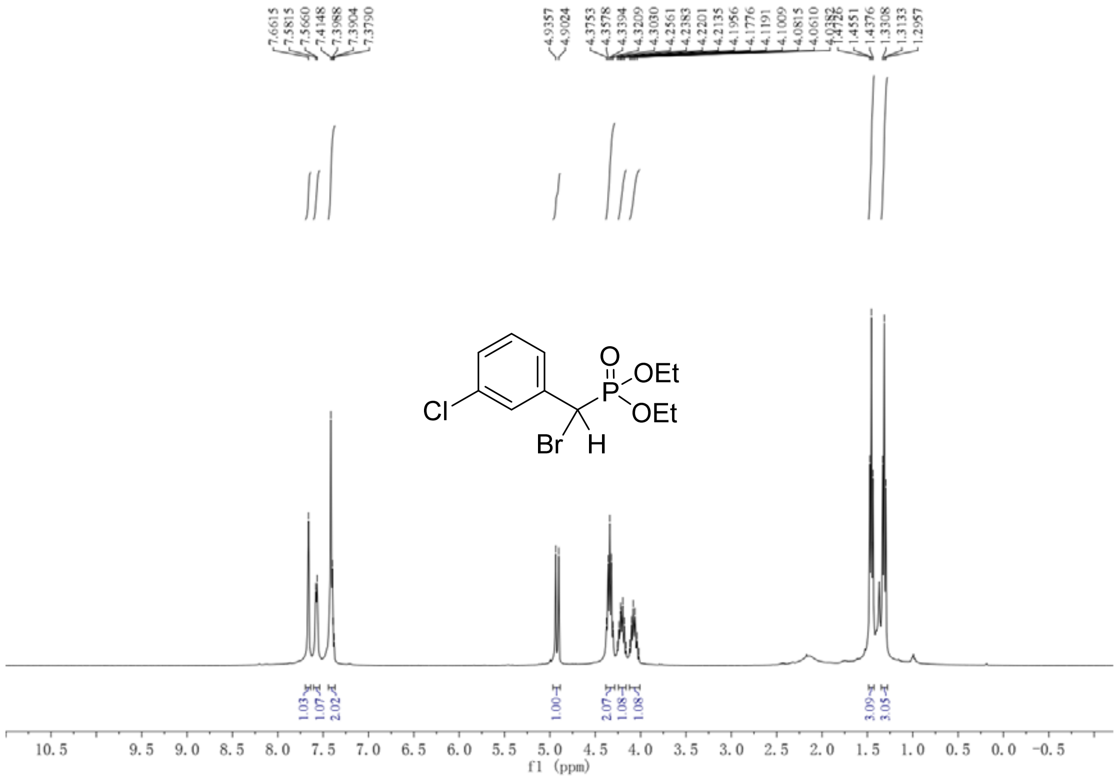

^{13}C NMR (100 MHz, $CDCl_3$) of **3f**:

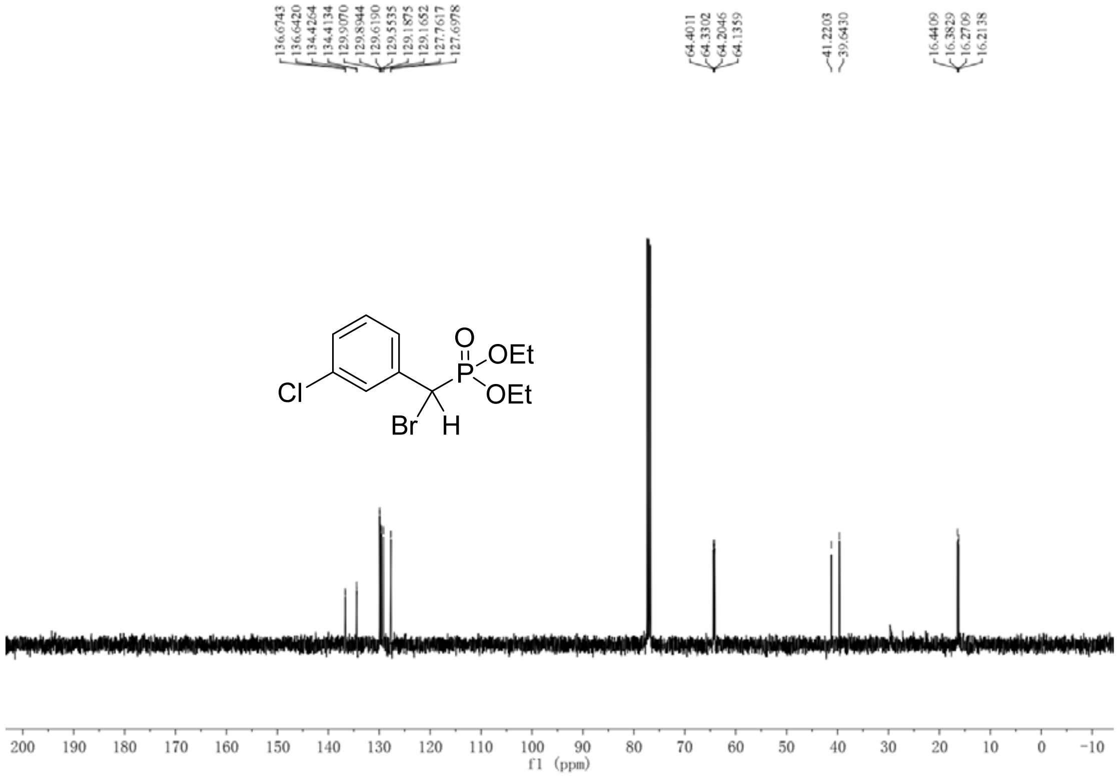

^{31}P NMR (162 MHz, $CDCl_3$) of **3f**:

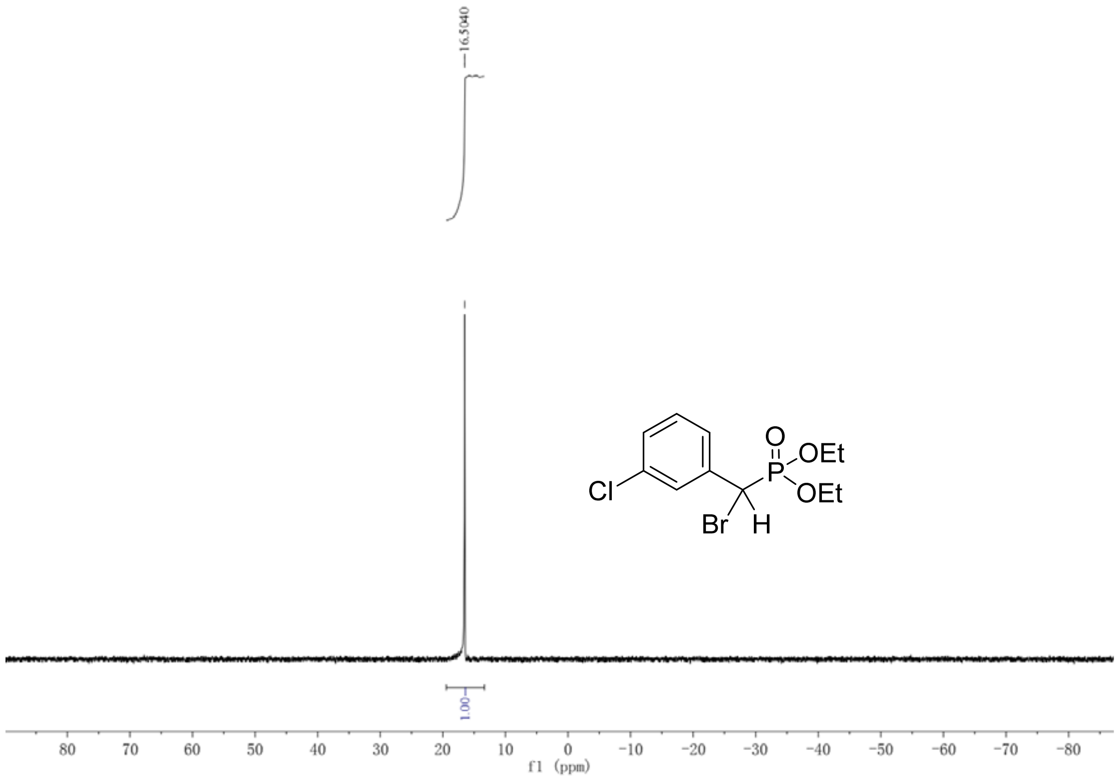

^{1}H NMR (400 MHz, CDCl3) of **3g**:

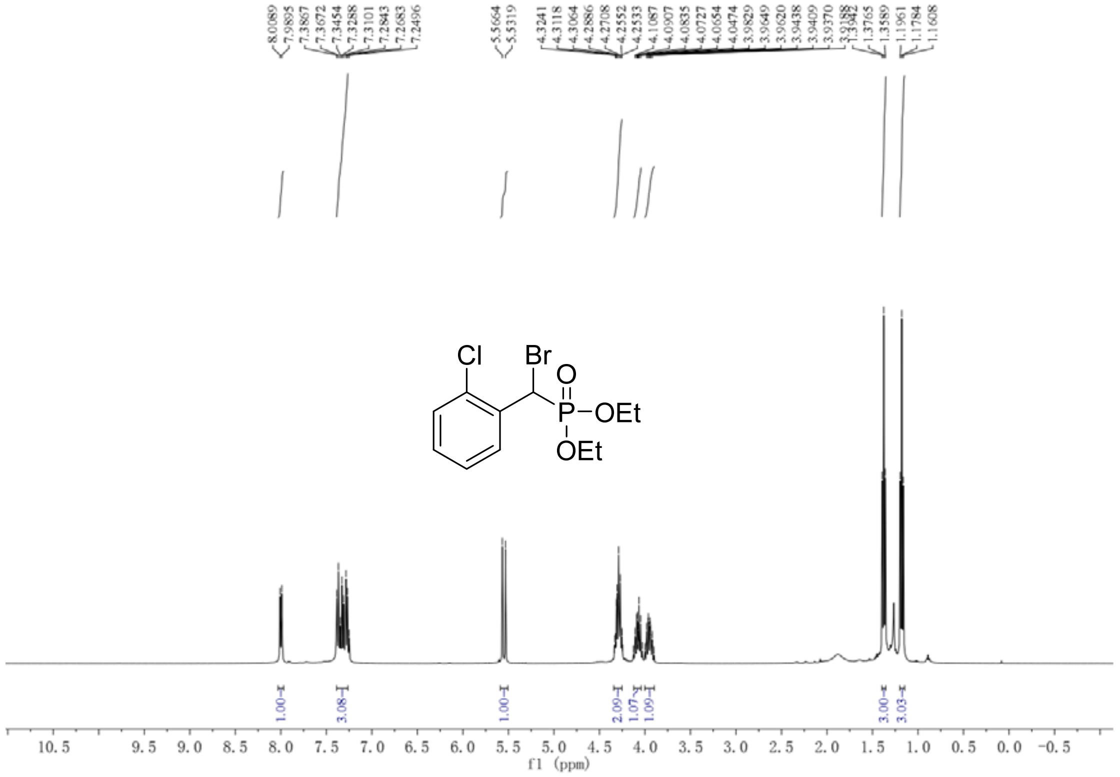

^{13}C NMR (100 MHz, $CDCl_3$) of **3g**:

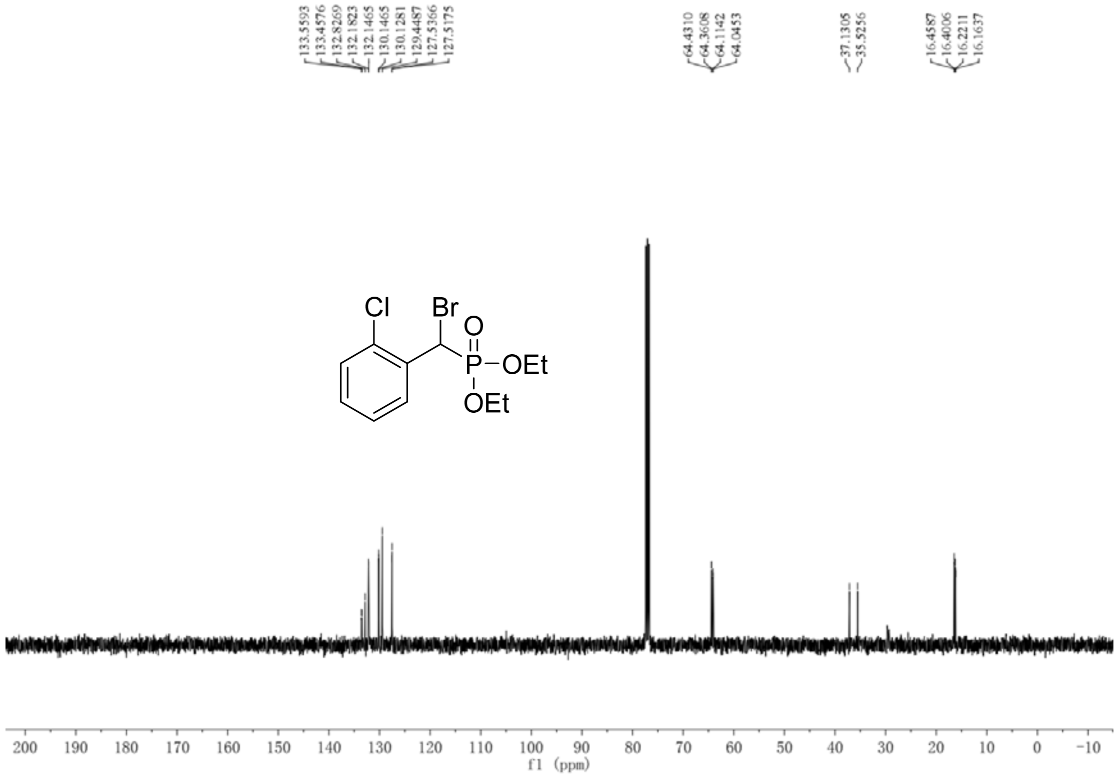

^{31}P NMR (162 MHz, $CDCl_3$) of **3g**:

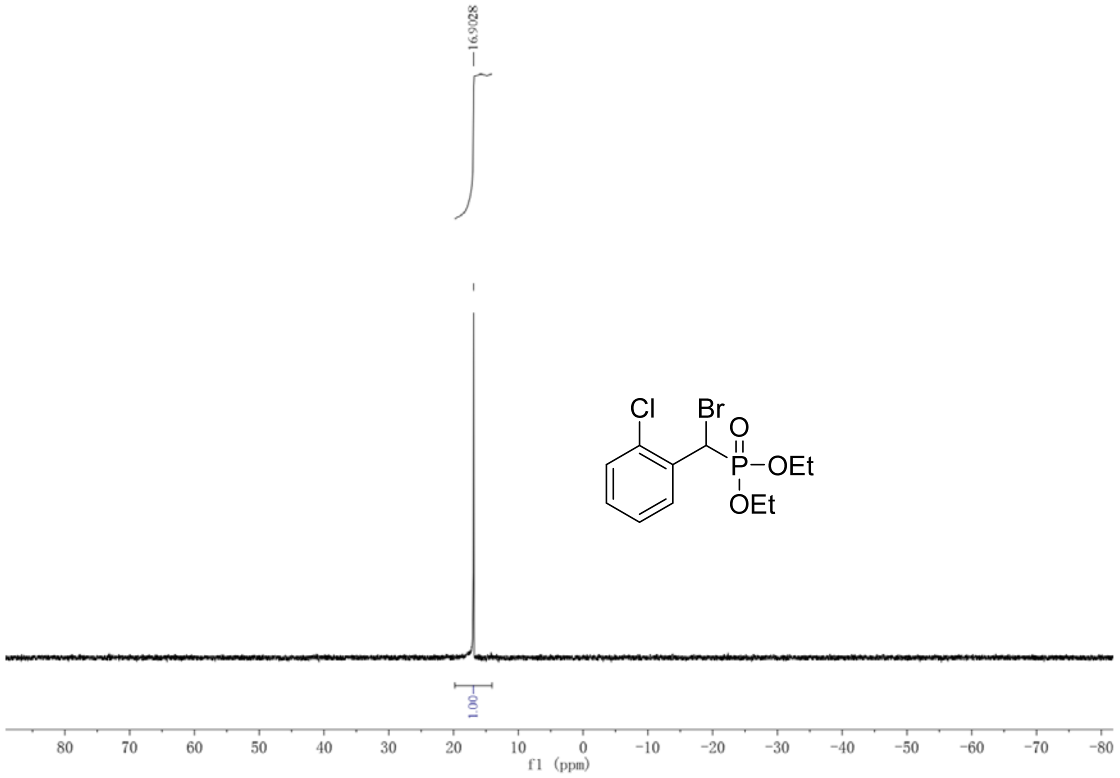

^{1}H NMR (400 MHz, CDCl3) of **3h**:

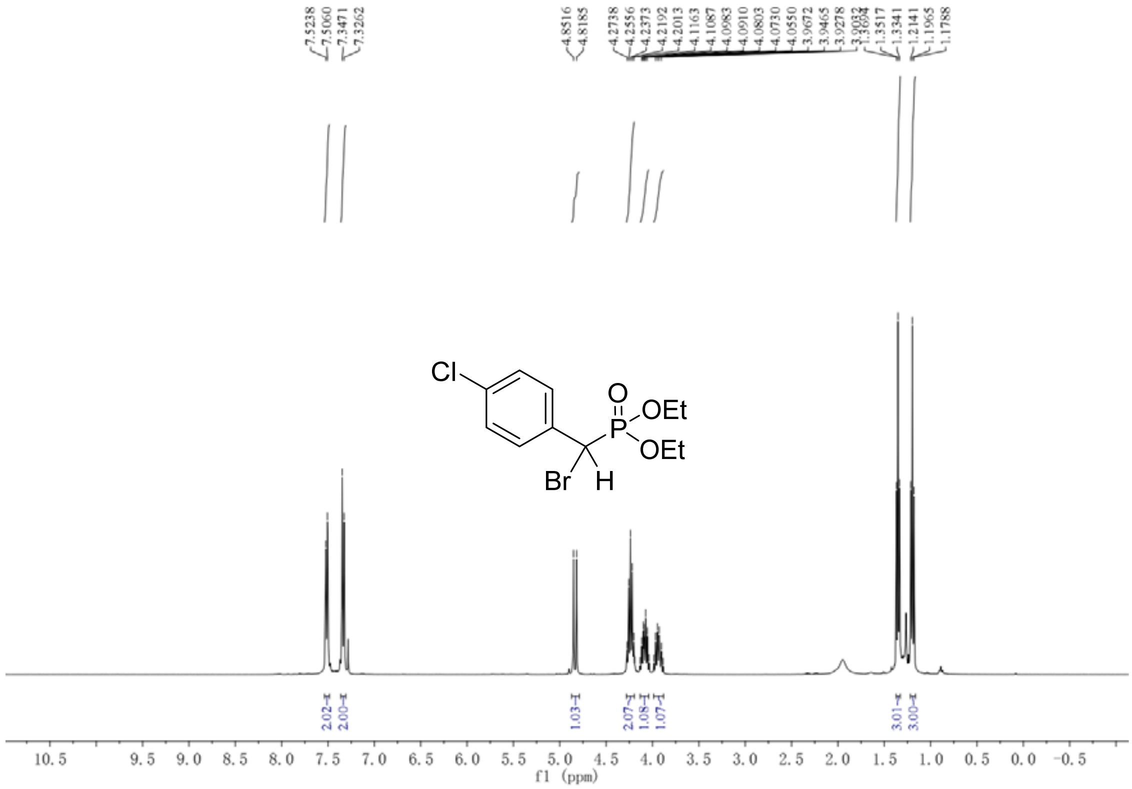

^{13}C NMR (100 MHz, $CDCl_3$) of **3h**:

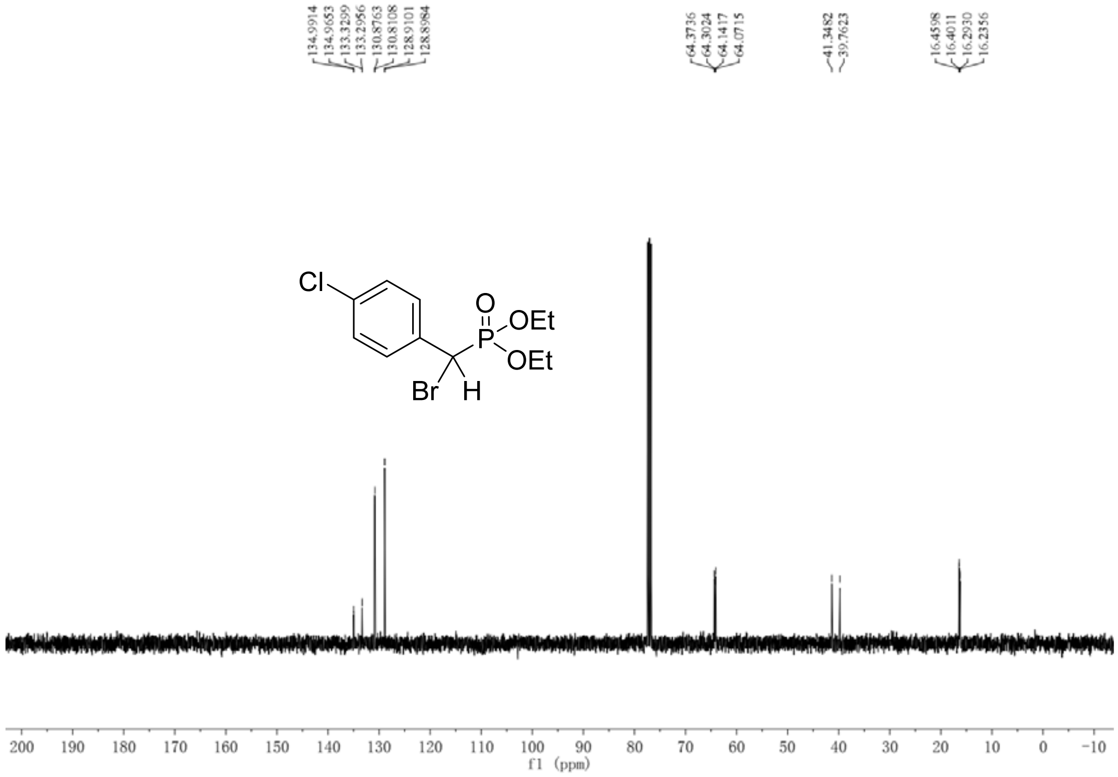

^{31}P NMR (162 MHz, $CDCl_3$) of **3h**:

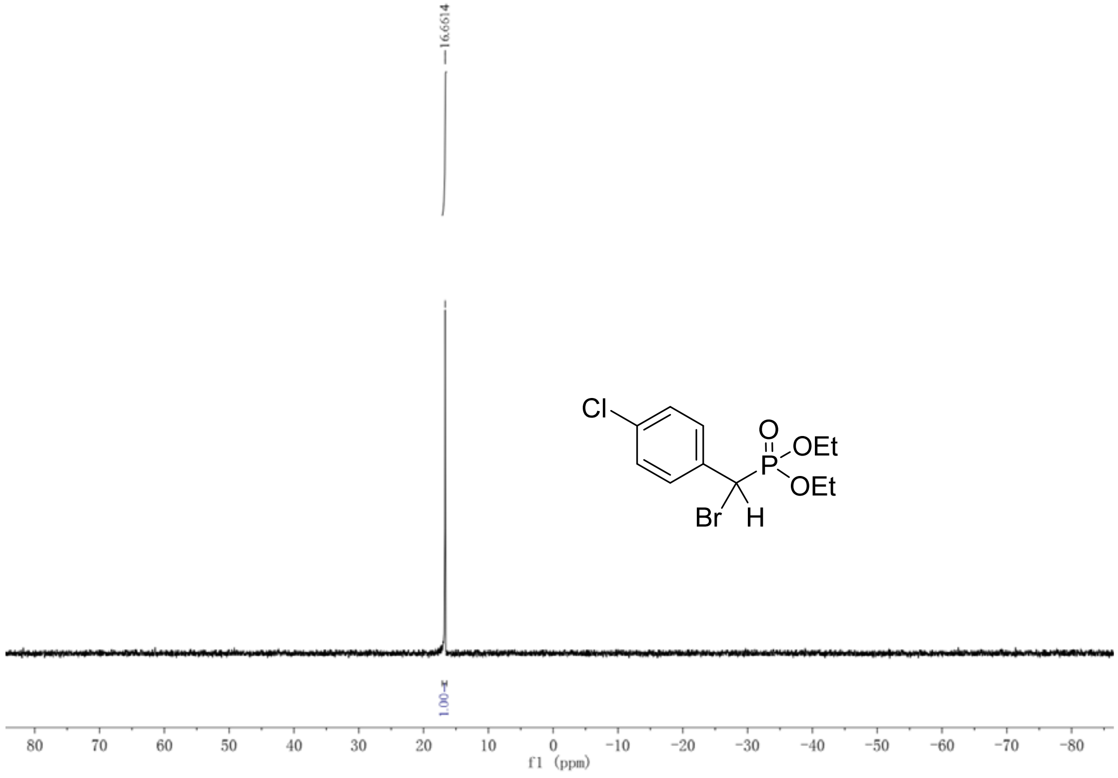

^{1}H NMR (400 MHz, CDCl3) of **3i**:

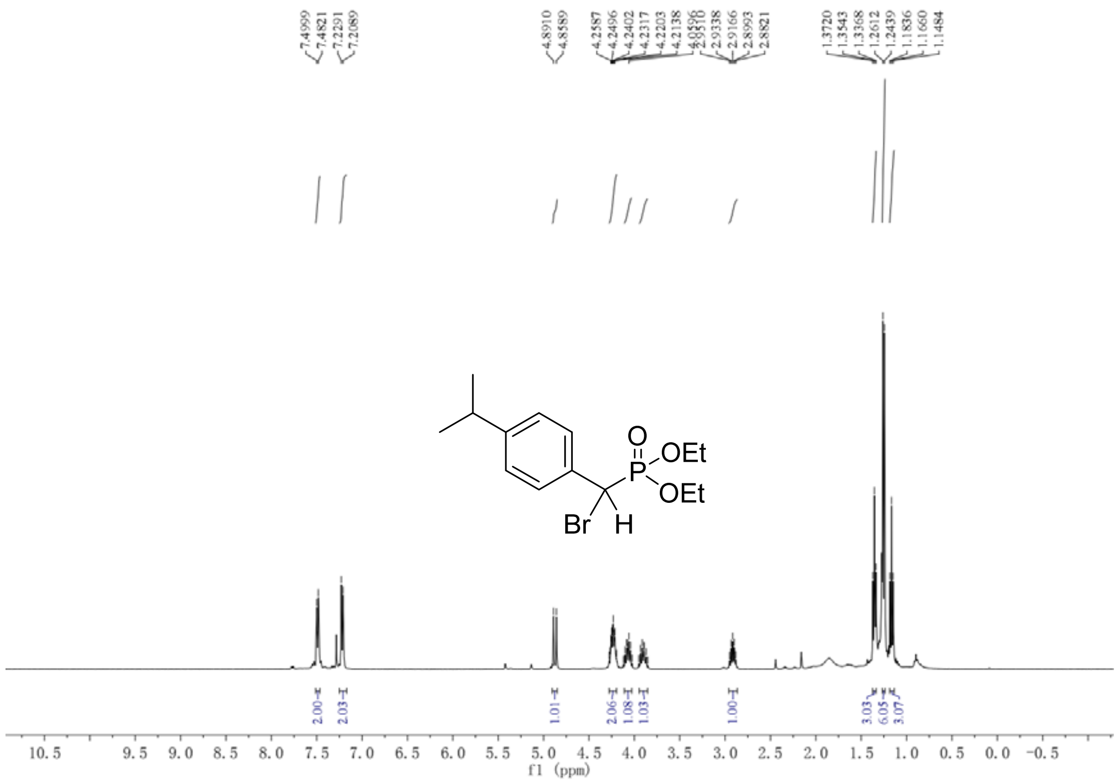

^{13}C NMR (100 MHz, $CDCl_3$) of **3i**:

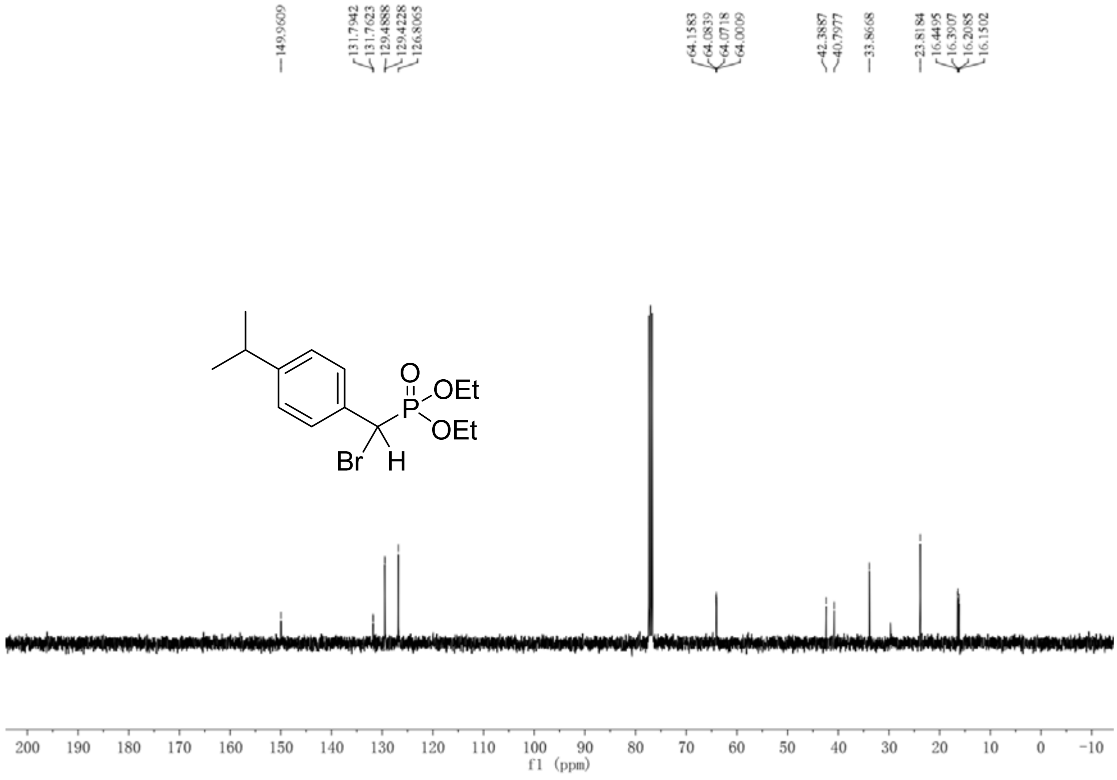

^{31}P NMR (162 MHz, $CDCl_3$) of **3i**:

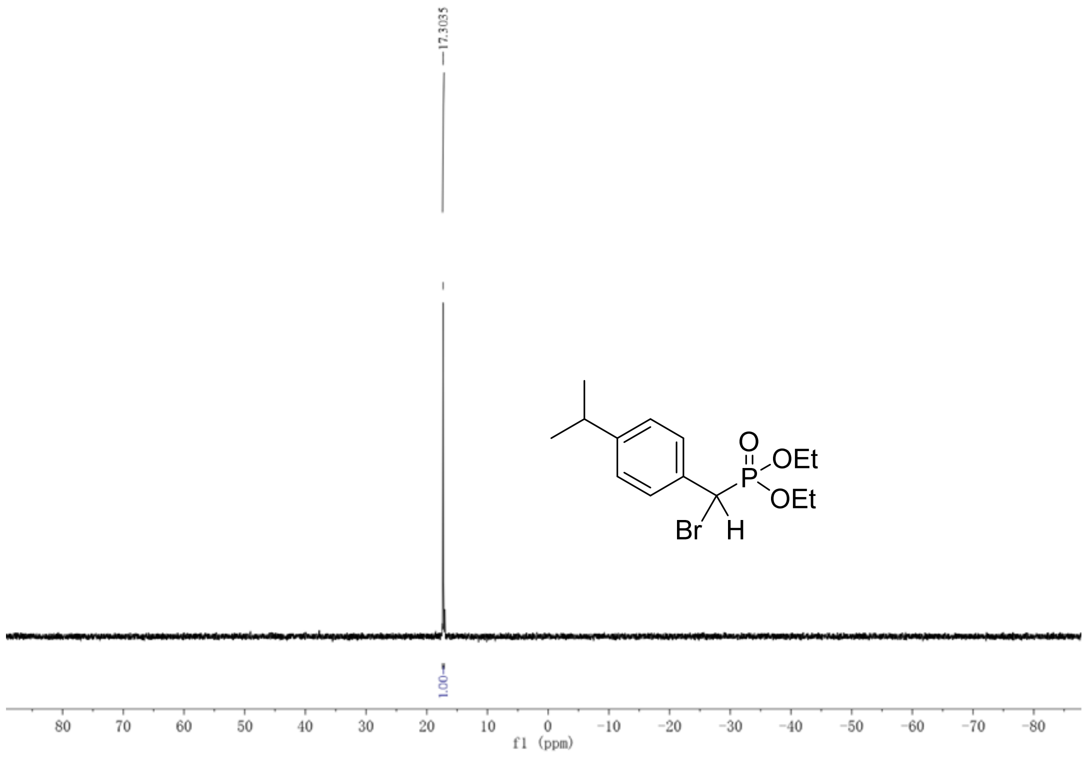

^{1}H NMR (400 MHz, CDCl3) of **3j**:

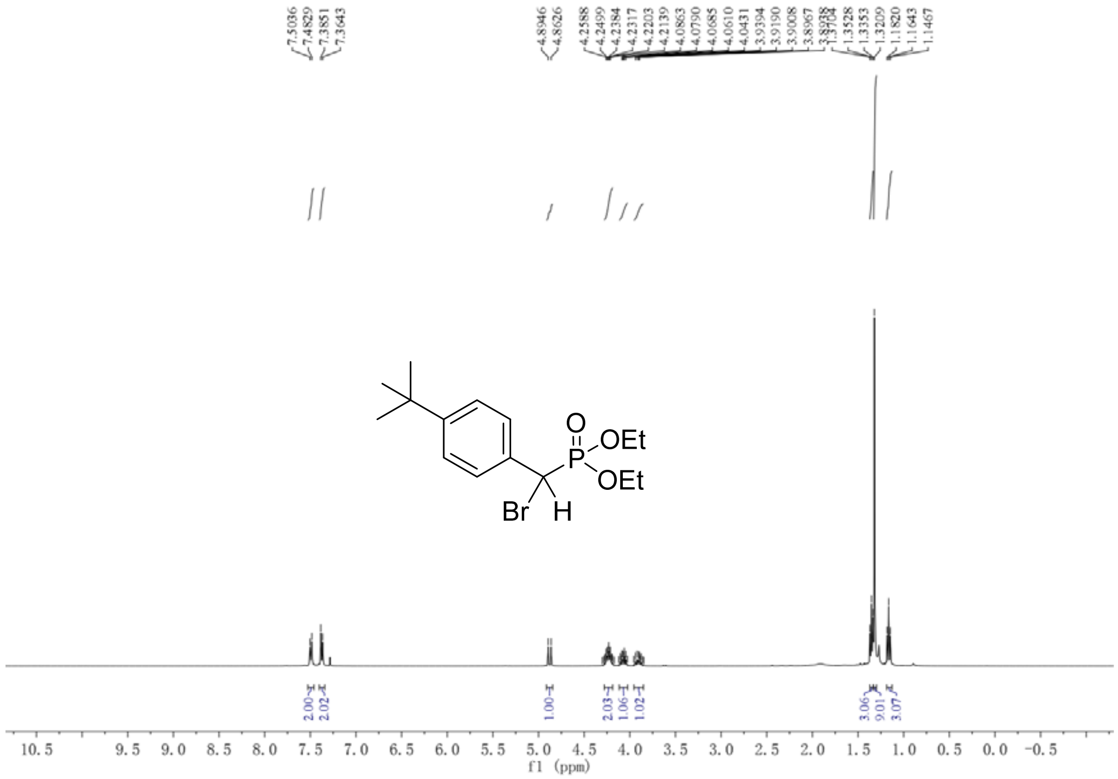

^{13}C NMR (100 MHz, $CDCl_3$) of **3j**:

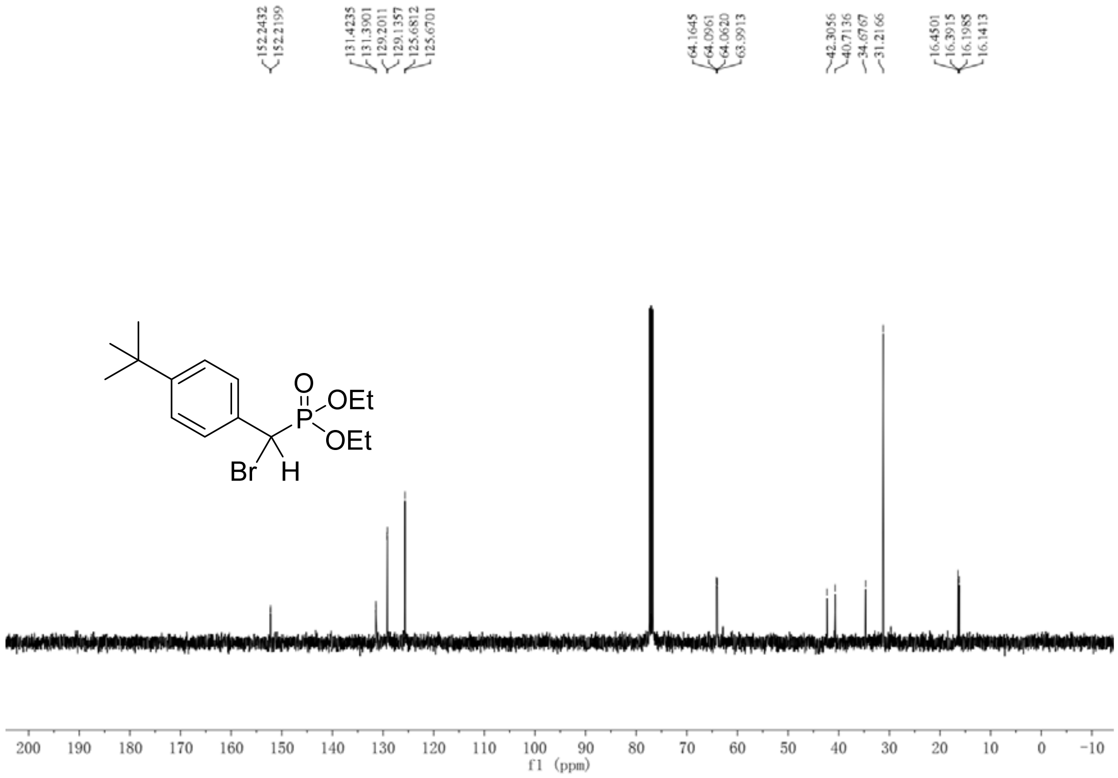

^{31}P NMR (162 MHz, $CDCl_3$) of **3j**:

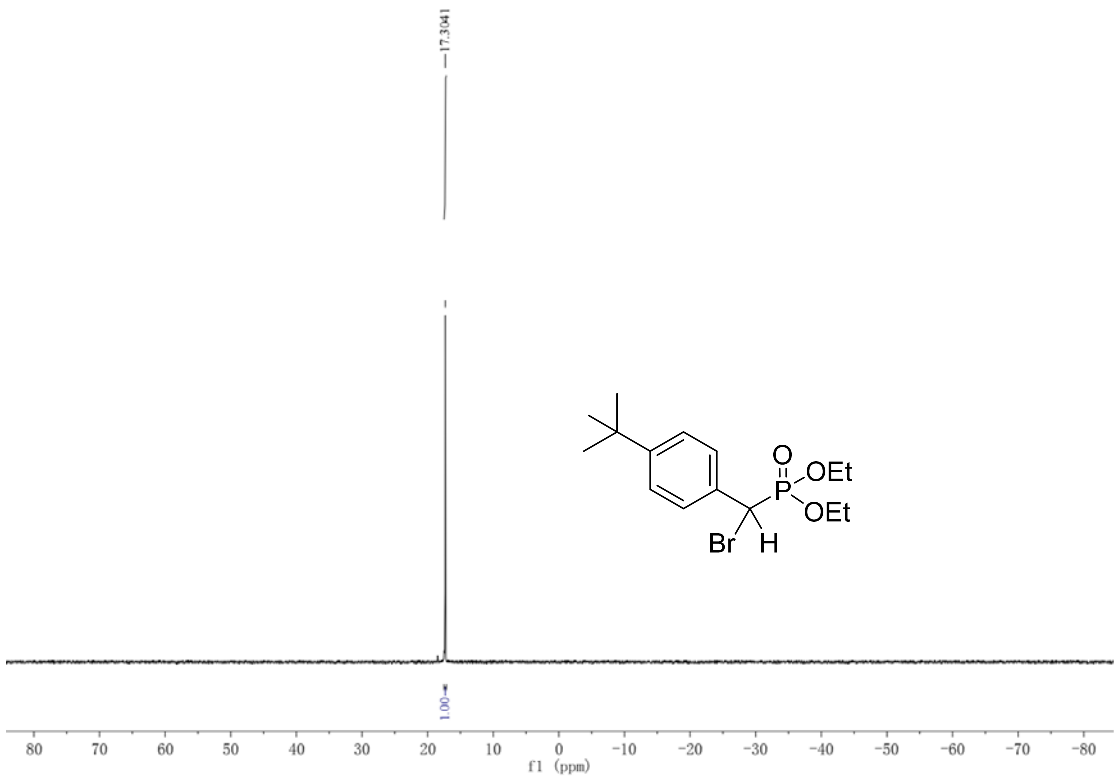

^{1}H NMR (600 MHz, CDCl3) of **3l**:

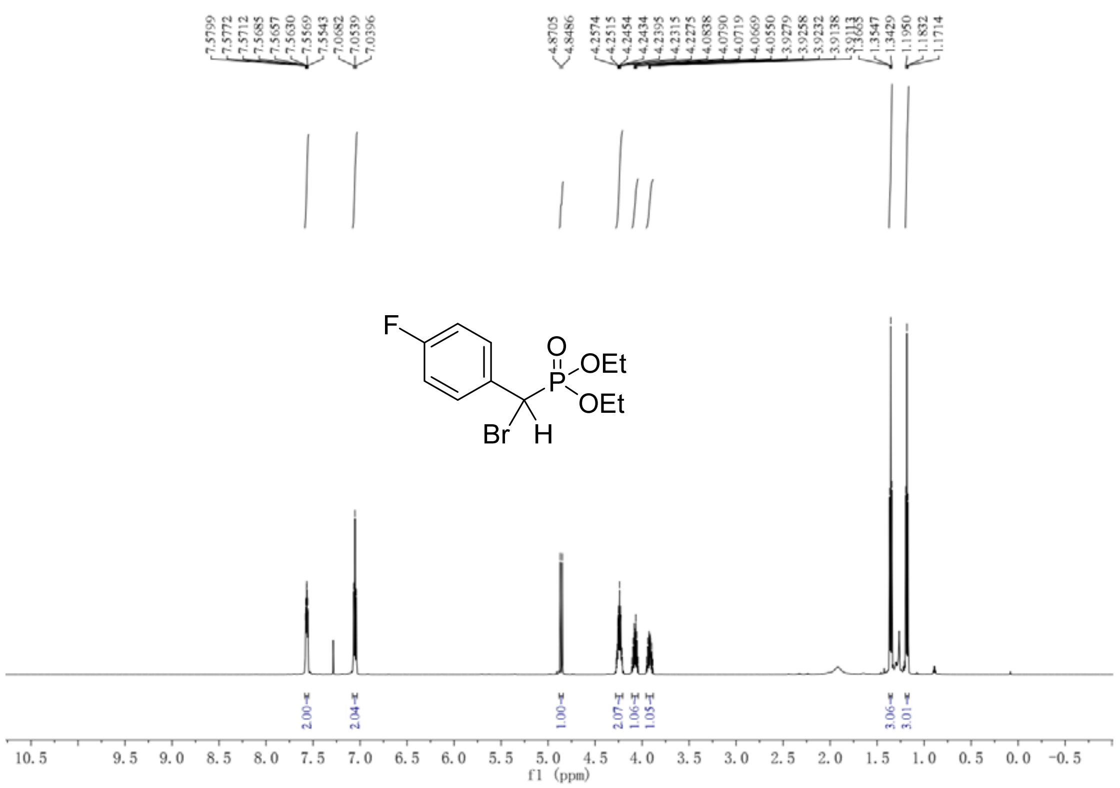

^{13}C NMR (100 MHz, $CDCl_3$) of **3l**:

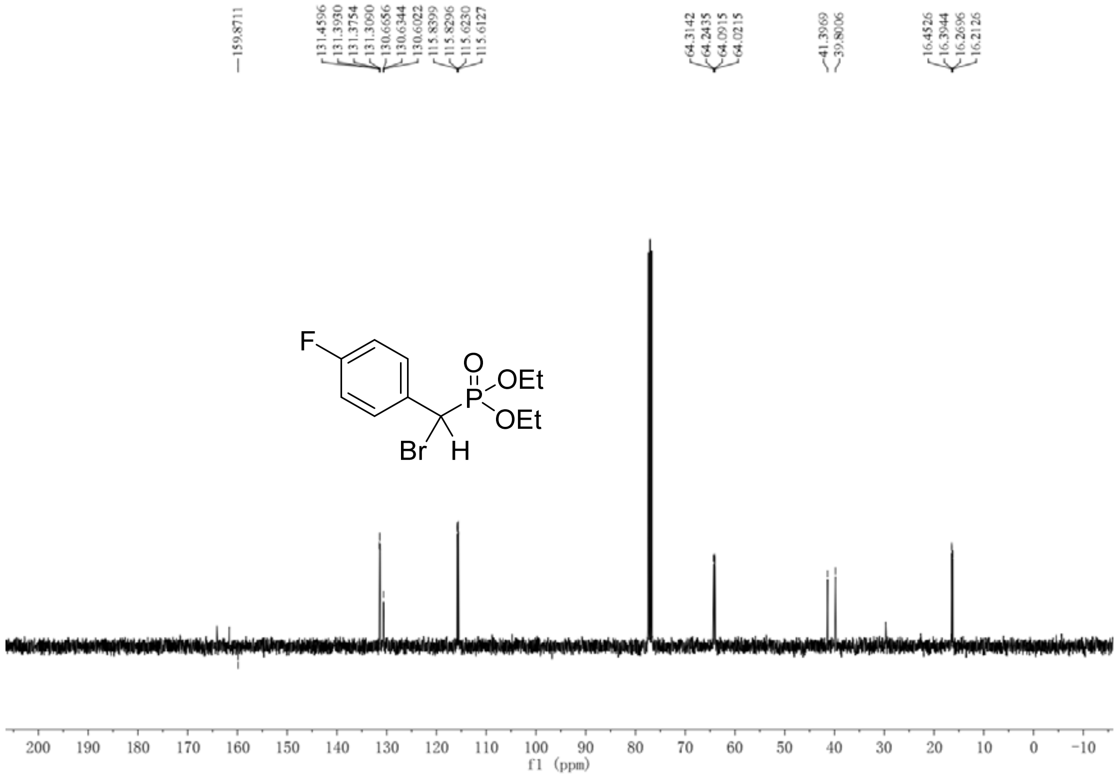

^{31}P NMR (162 MHz, $CDCl_3$) of **3l**:

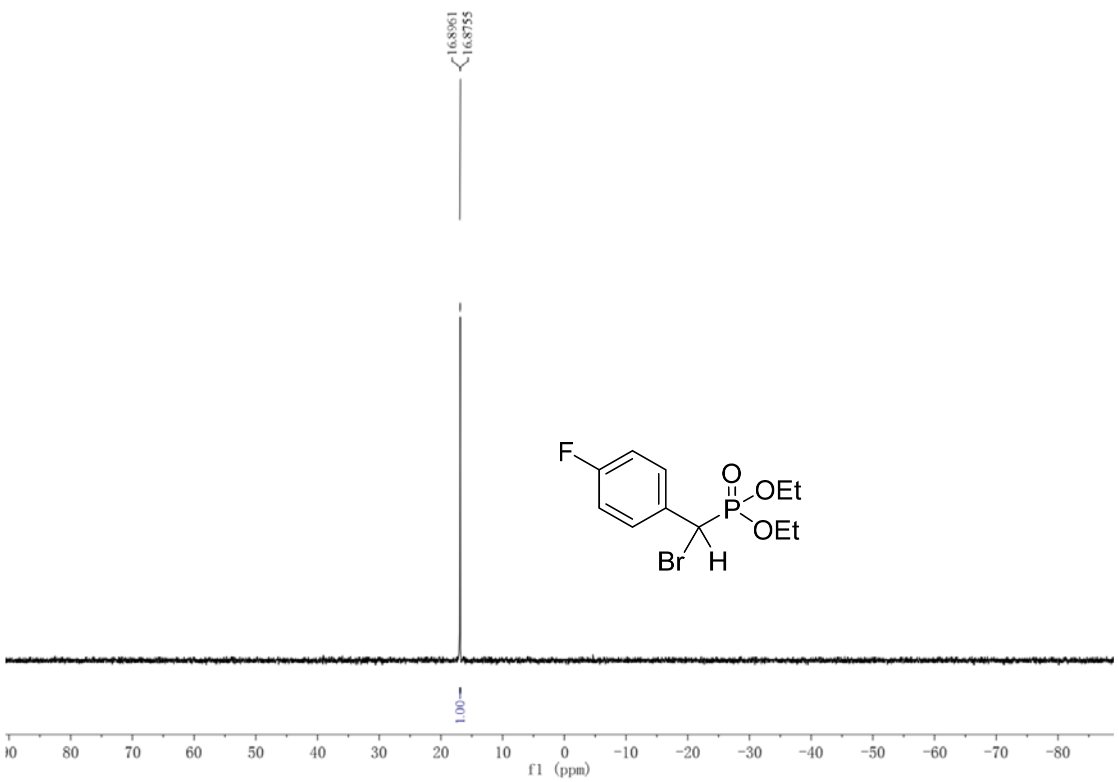

^{19}F NMR (376 MHz, $CDCl_3$) of **3l**:

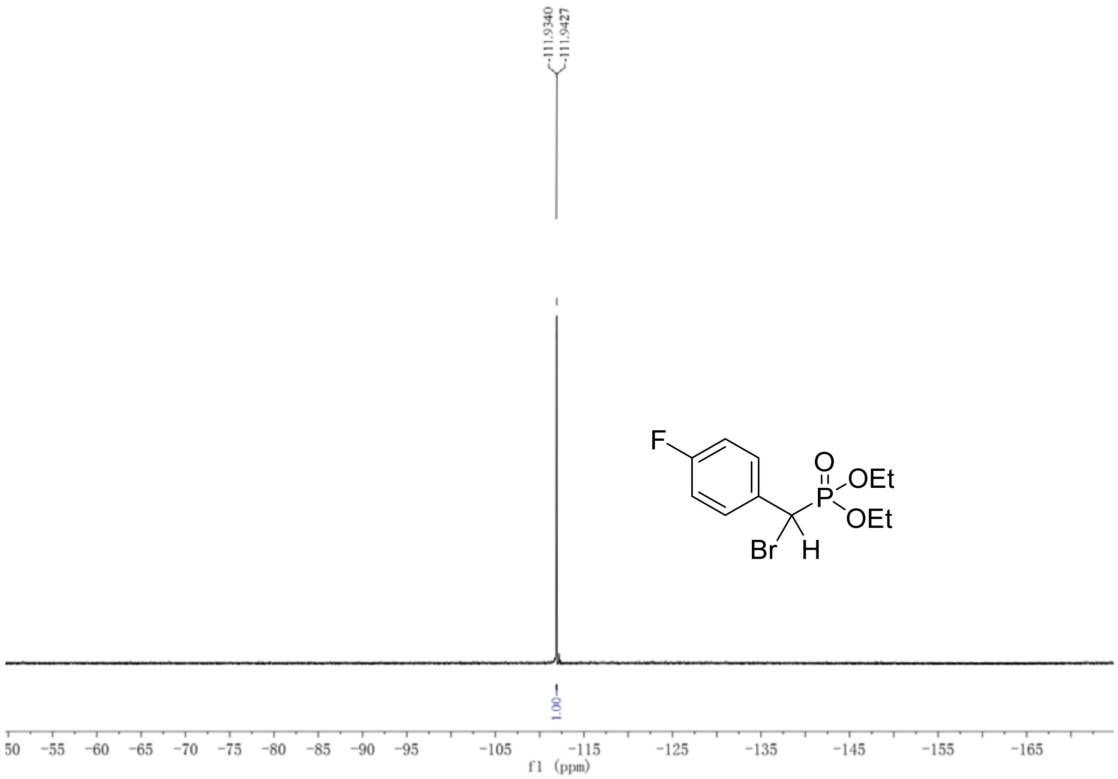

^{1}H NMR (600 MHz, CDCl3) of **3m**:

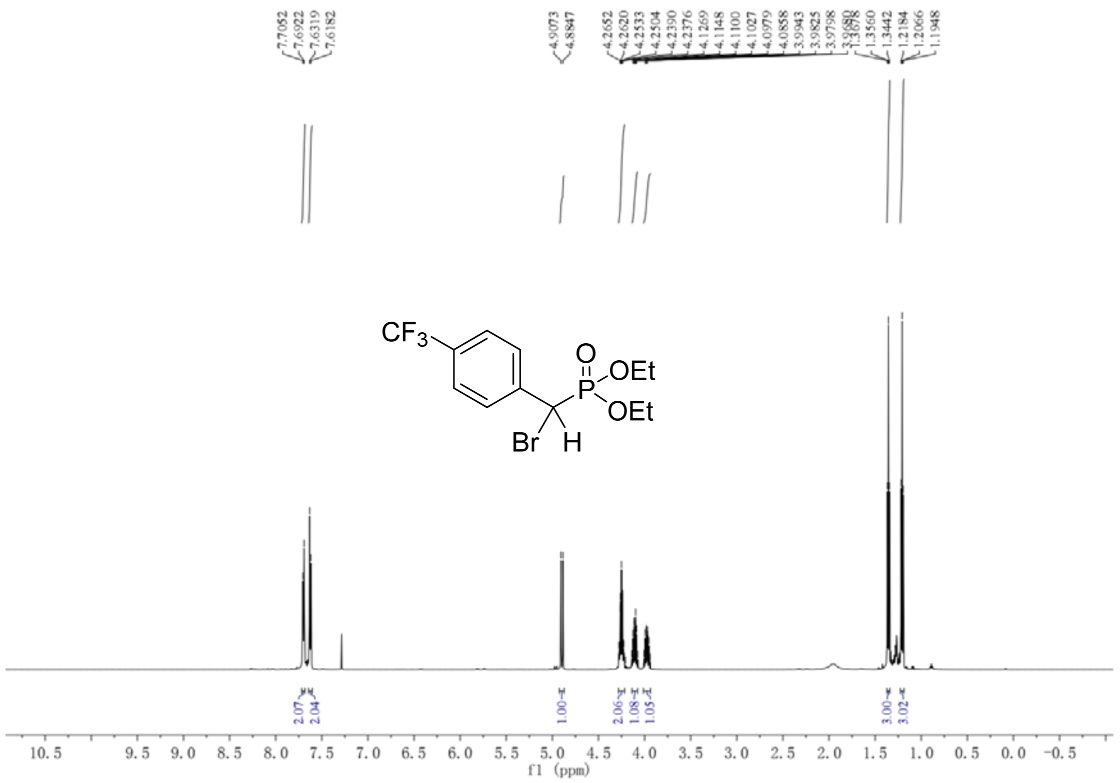

^{13}C NMR (100 MHz, $CDCl_3$) of **3m**:

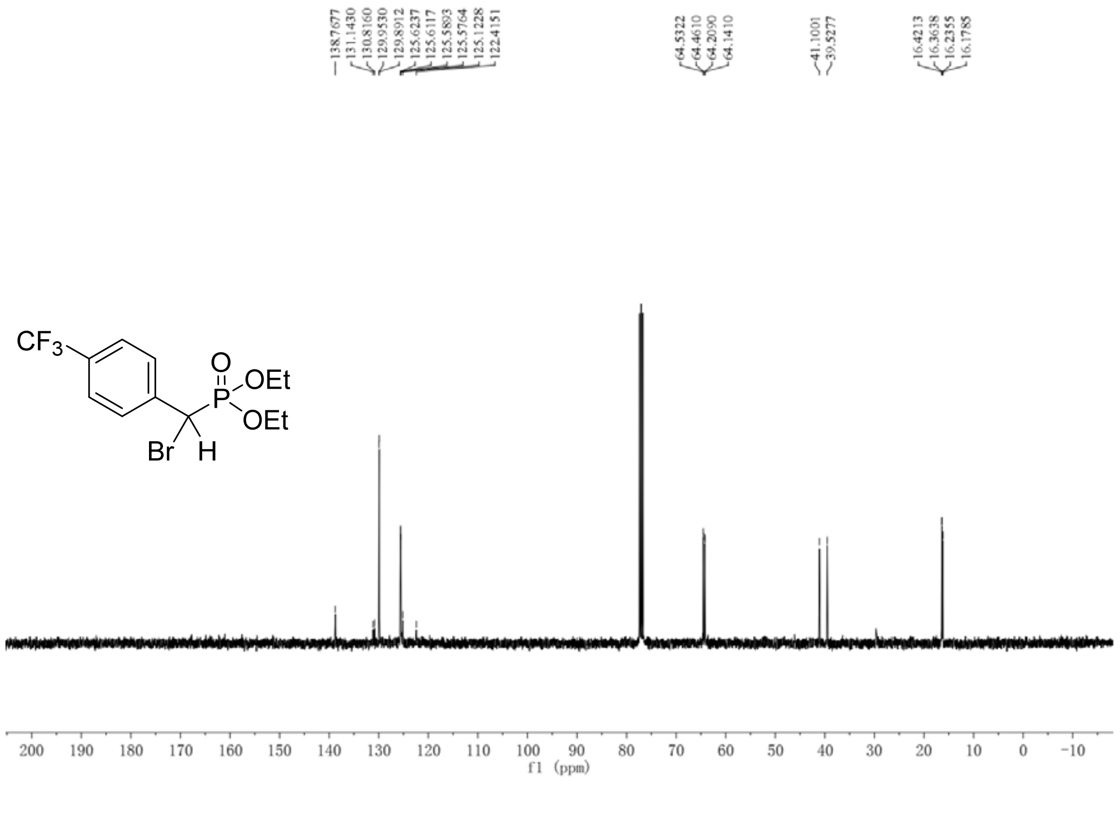

^{31}P NMR (162 MHz, $CDCl_3$) of **3m**:

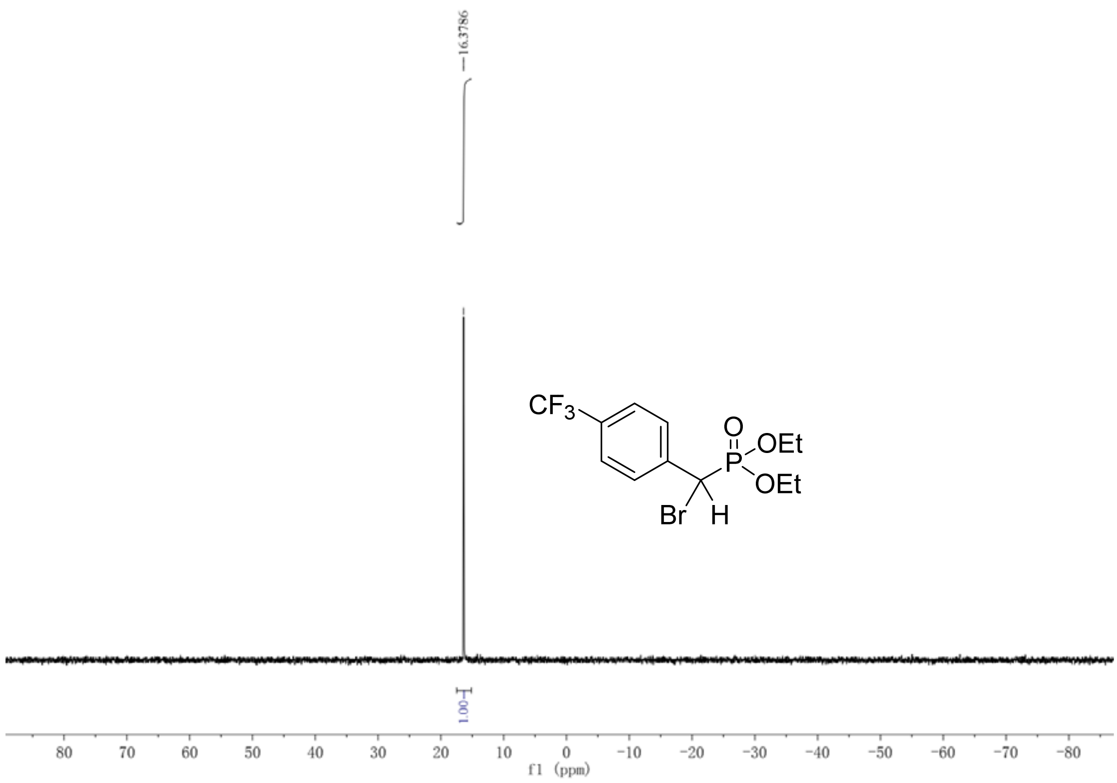

^{19}F NMR (376 MHz, $CDCl_3$) of **3m**:

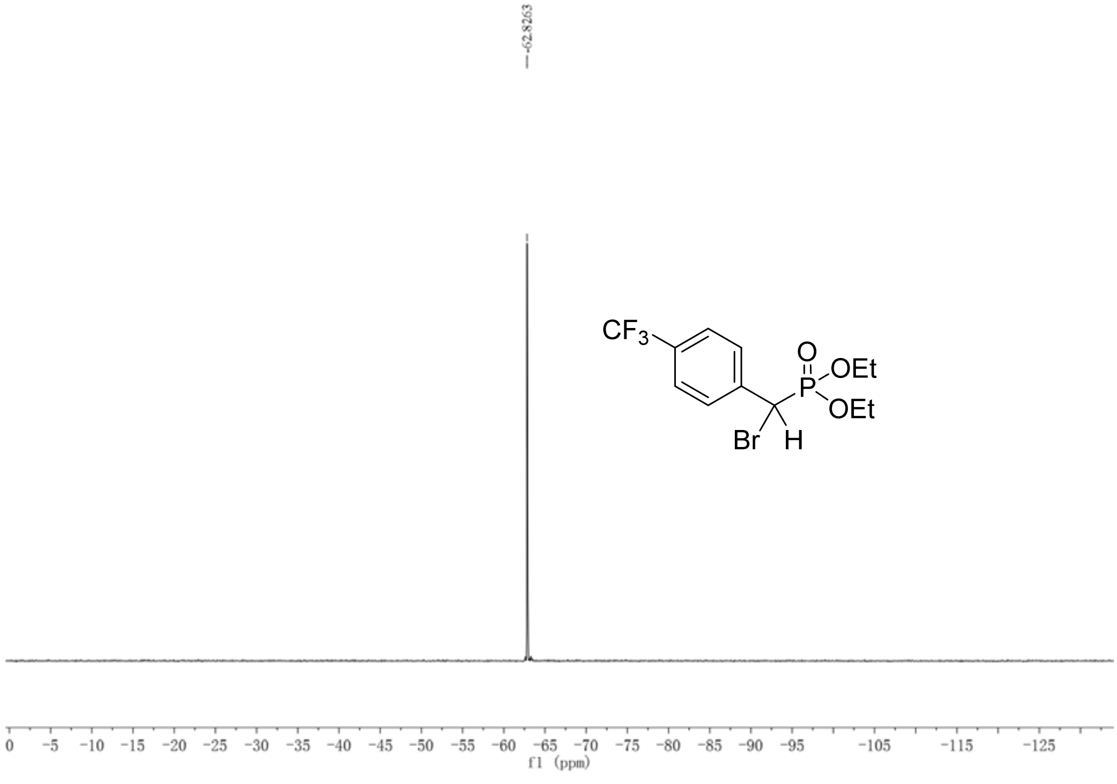

^{1}H NMR (600 MHz, CDCl3) of **3n**:

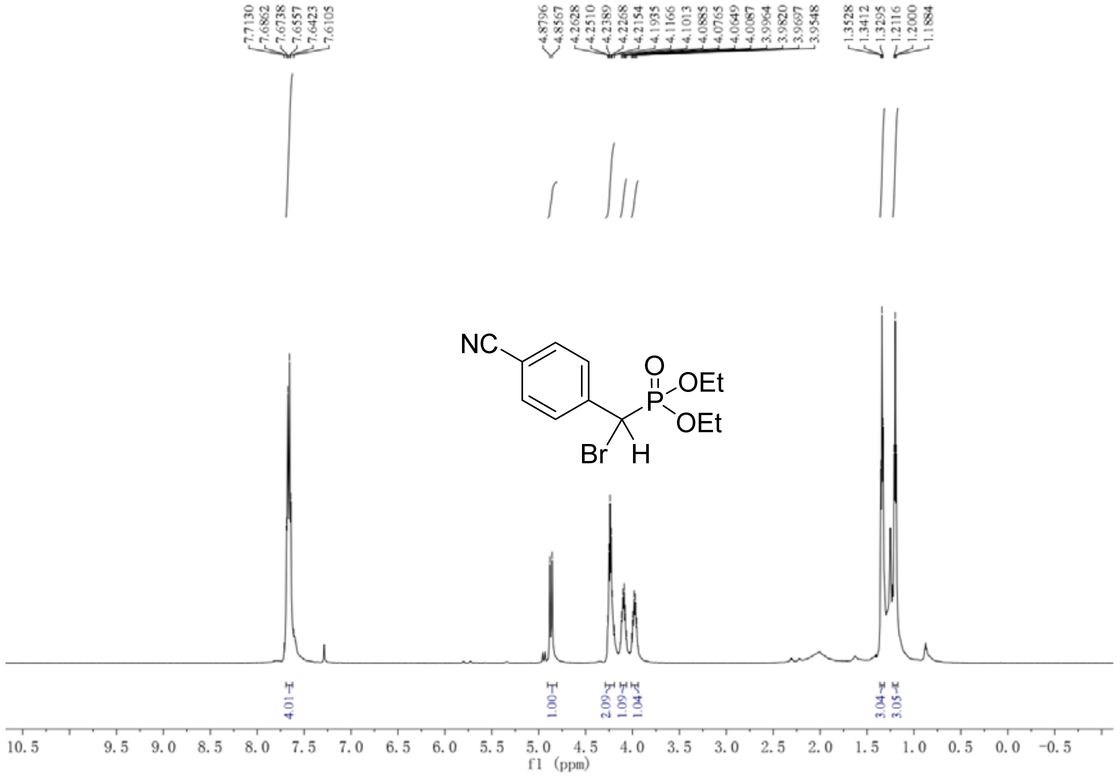

^{13}C NMR (100 MHz, $CDCl_3$) of **3n**:

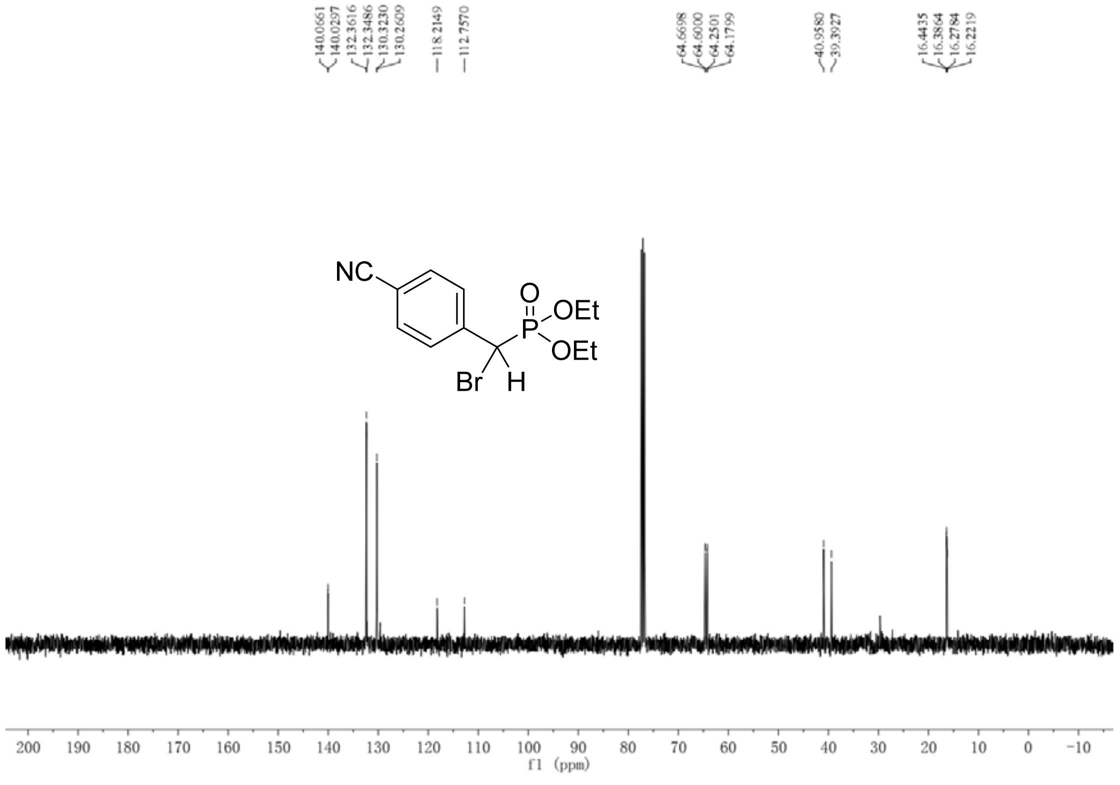

^{31}P NMR (162 MHz, $CDCl_3$) of **3n**:

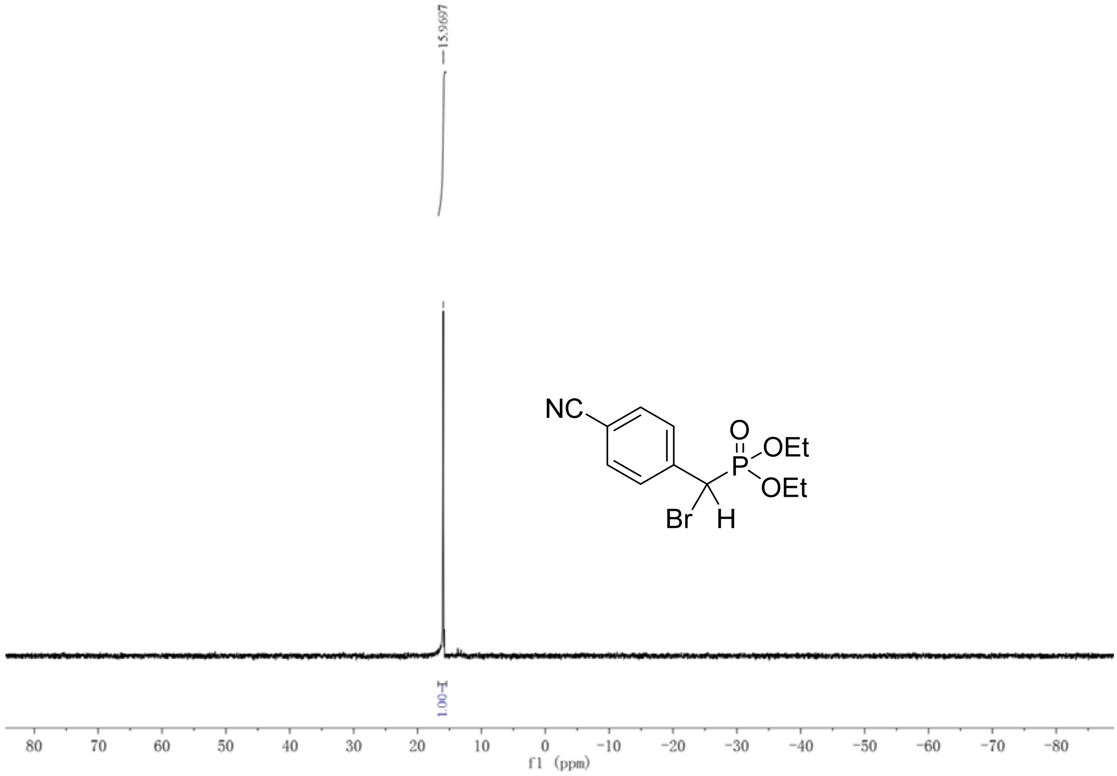

^{1}H NMR (600 MHz, CDCl3) of **3o**:

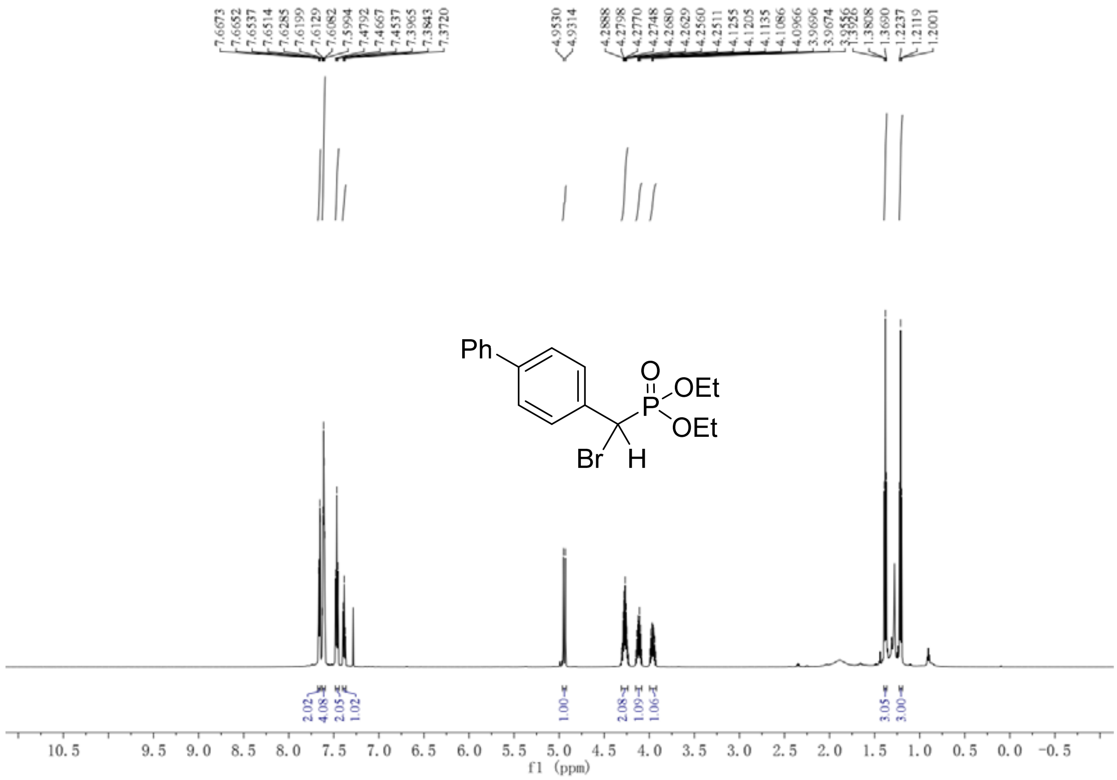

^{13}C NMR (100 MHz, CDCl$_3$) of **3o**:

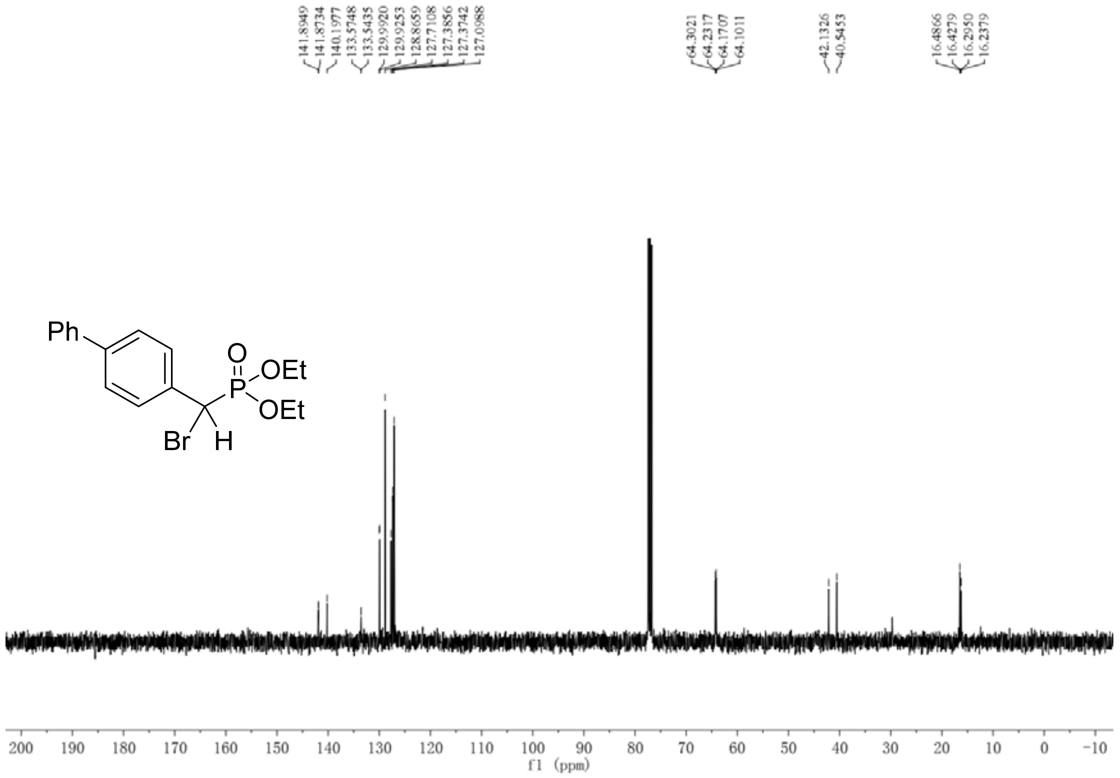

^{31}P NMR (162 MHz, CDCl$_3$) of **3o**:

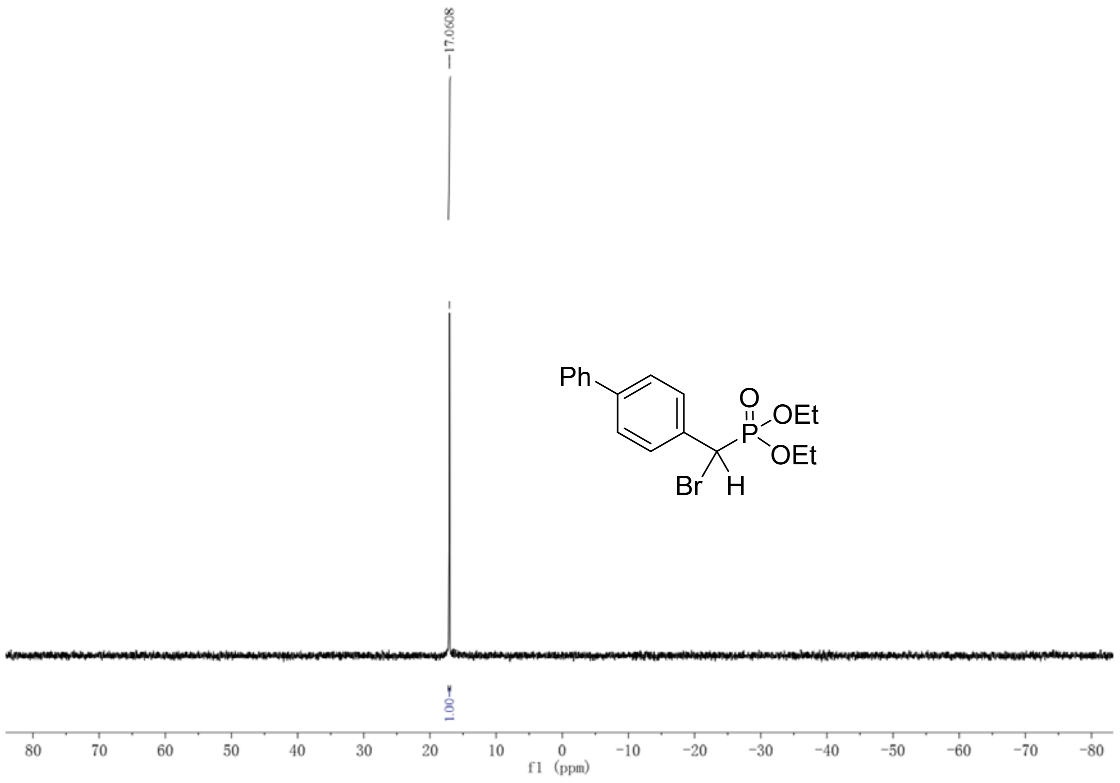

^{1}H NMR (400 MHz, CDCl3) of **3p**:

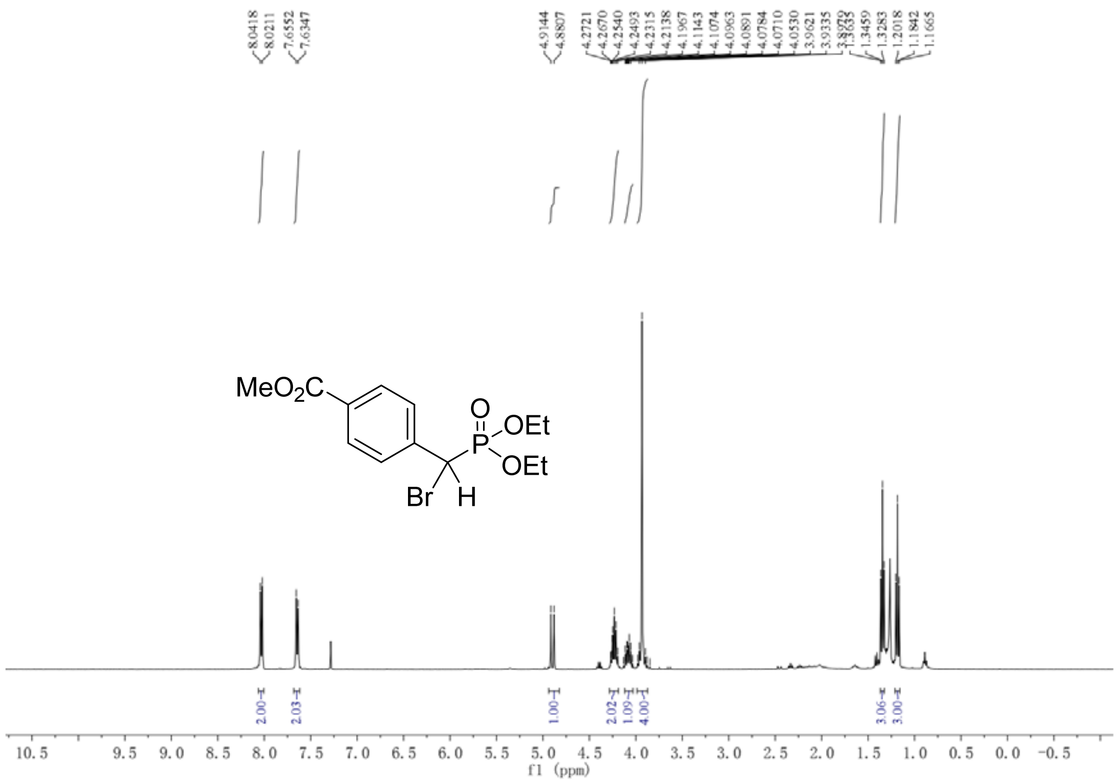

^{13}C NMR (100 MHz, $CDCl_3$) of **3p**:

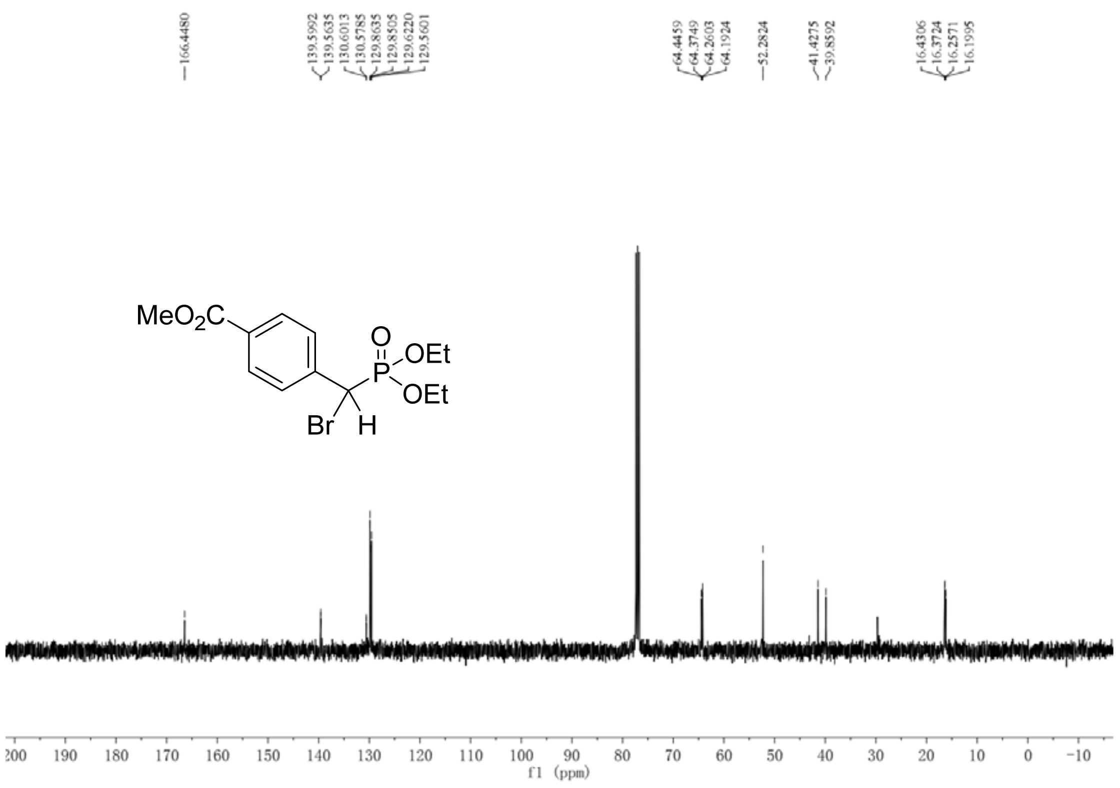

^{31}P NMR (162 MHz, $CDCl_3$) of **3p**:

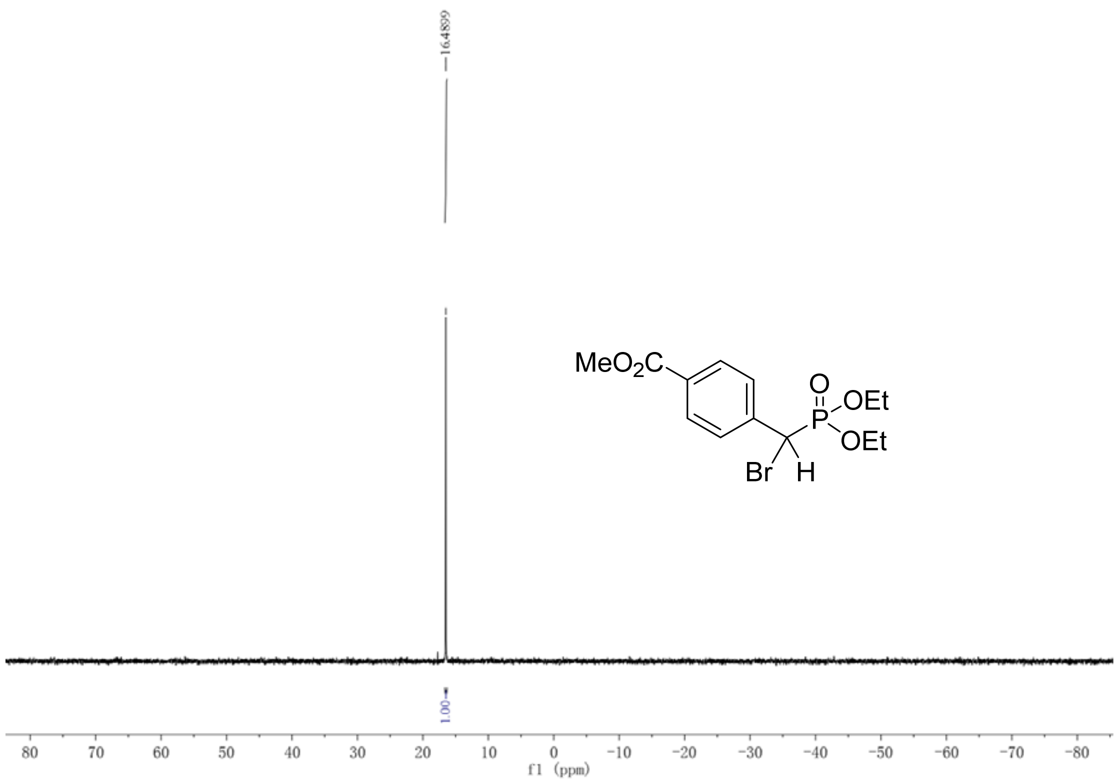

^1H NMR (400 MHz, CDCl3) of **3q**:

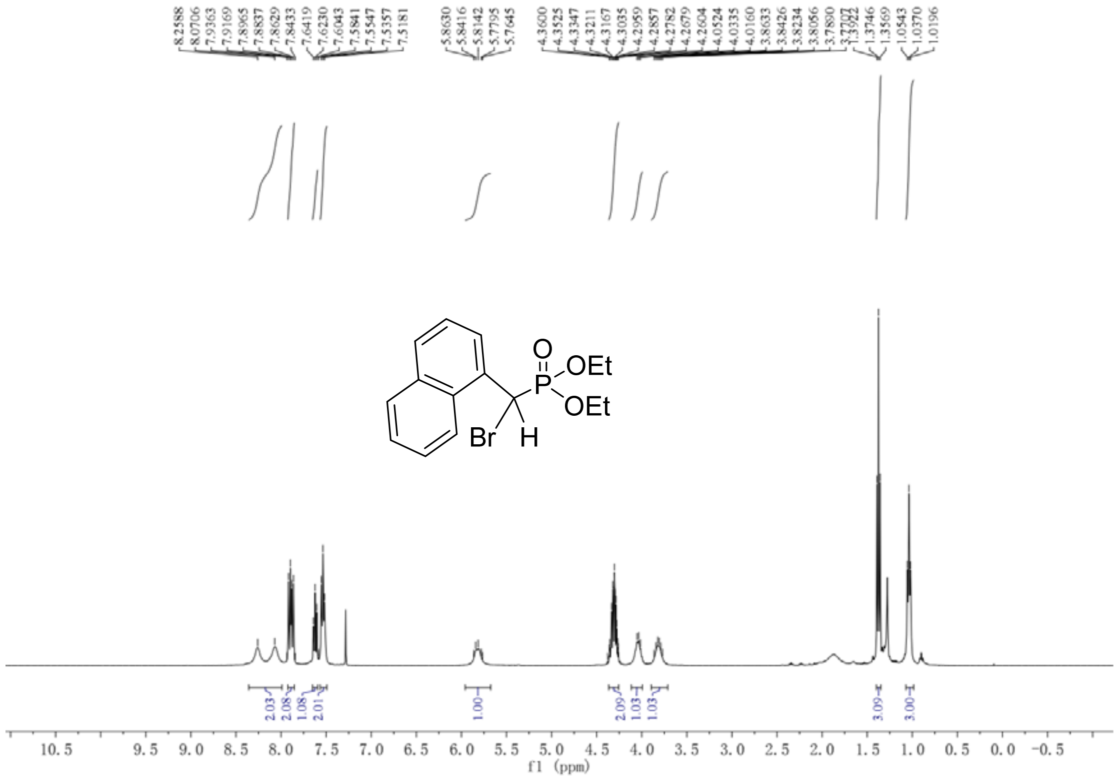

^{13}C NMR (100 MHz, $CDCl_3$) of **3q**:

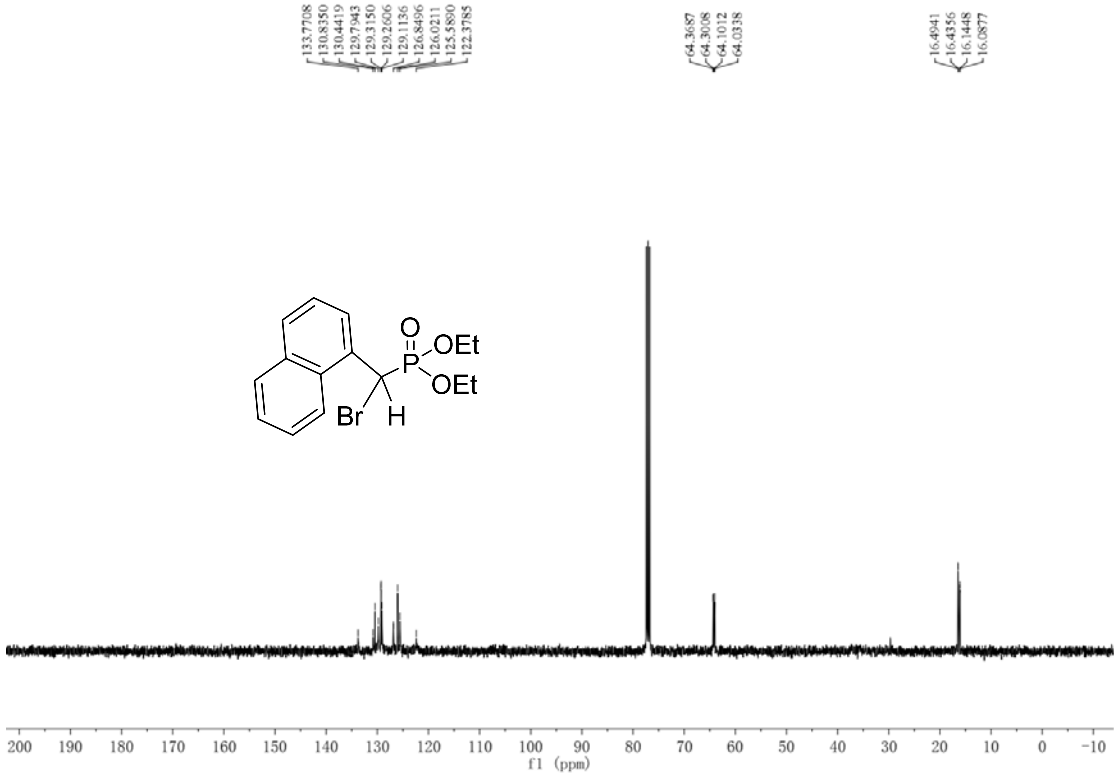

^{31}P NMR (162 MHz, $CDCl_3$) of **3q**:

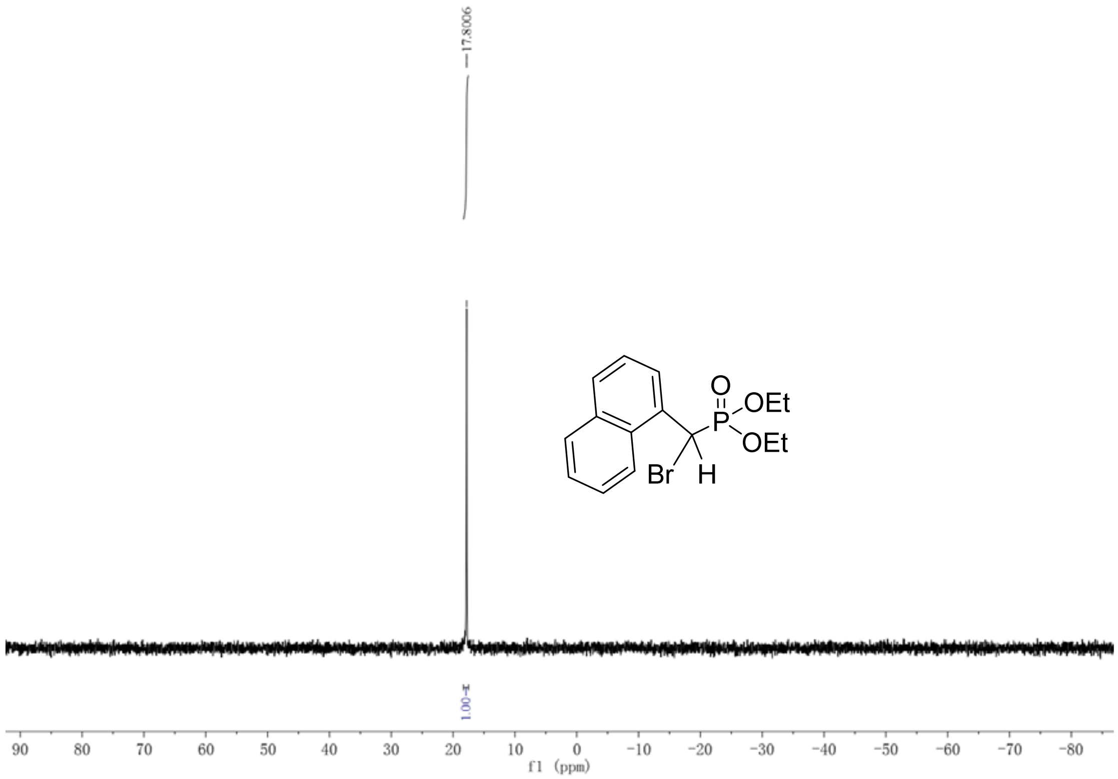

^{1}H NMR (400 MHz, CDCl3) of **3r**:

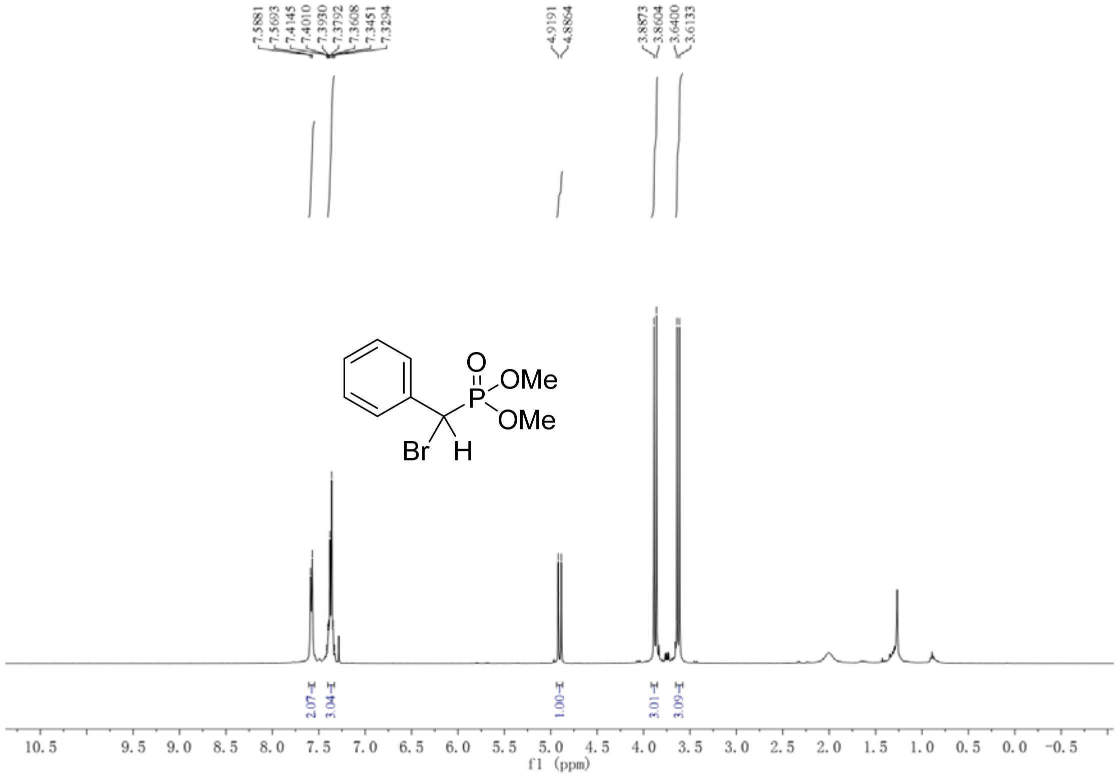

^{13}C NMR (100 MHz, $CDCl_3$) of **3r**:

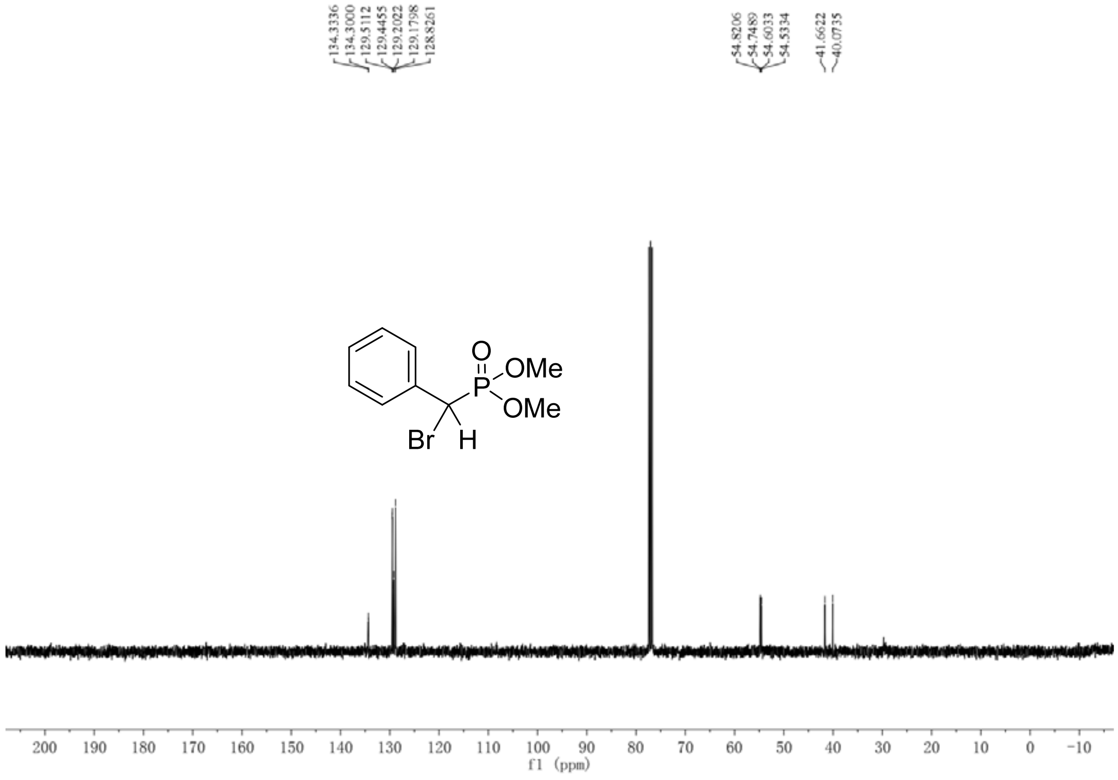

^{31}P NMR (162 MHz, $CDCl_3$) of **3r**:

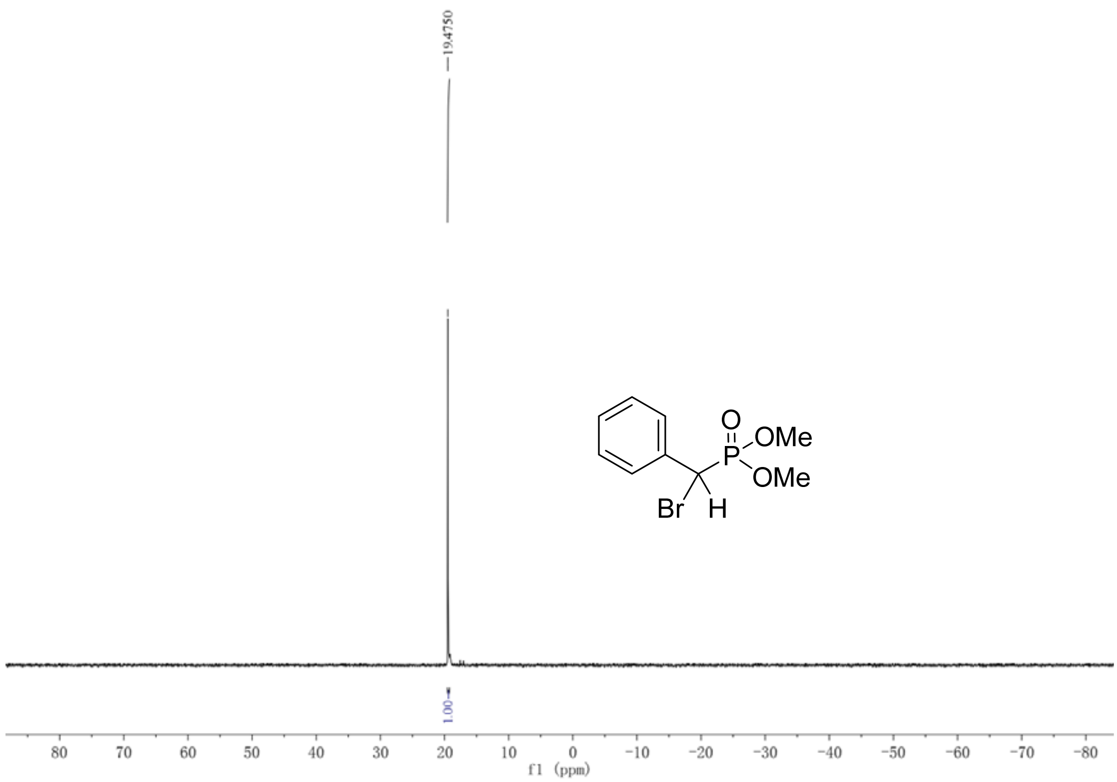

^{1}H NMR (400 MHz, CDCl3) of **3s**:

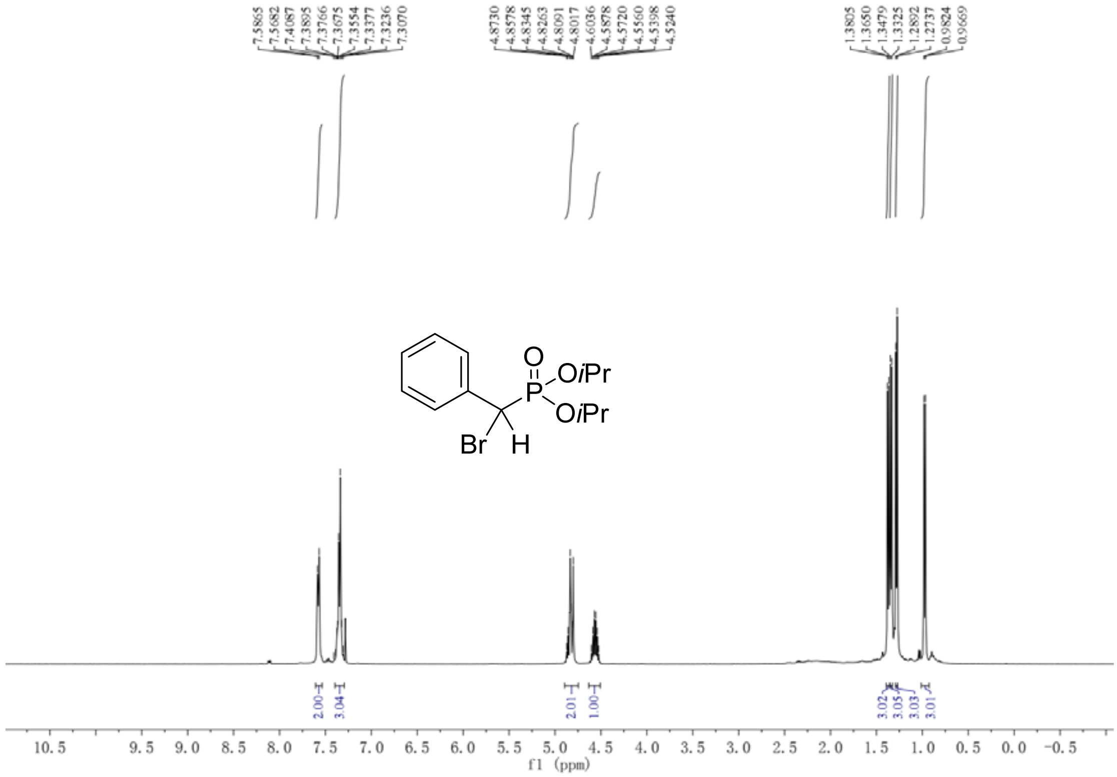

^{13}C NMR (100 MHz, $CDCl_3$) of **3s**:

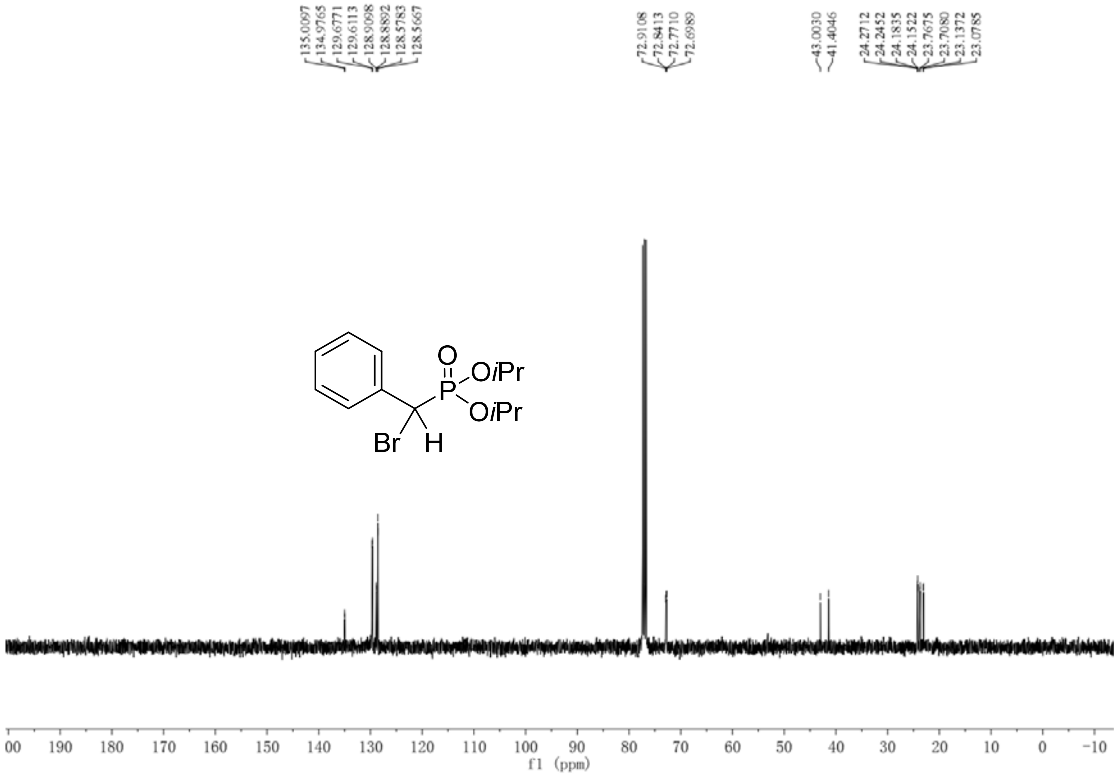

^{31}P NMR (162 MHz, $CDCl_3$) of **3s**:

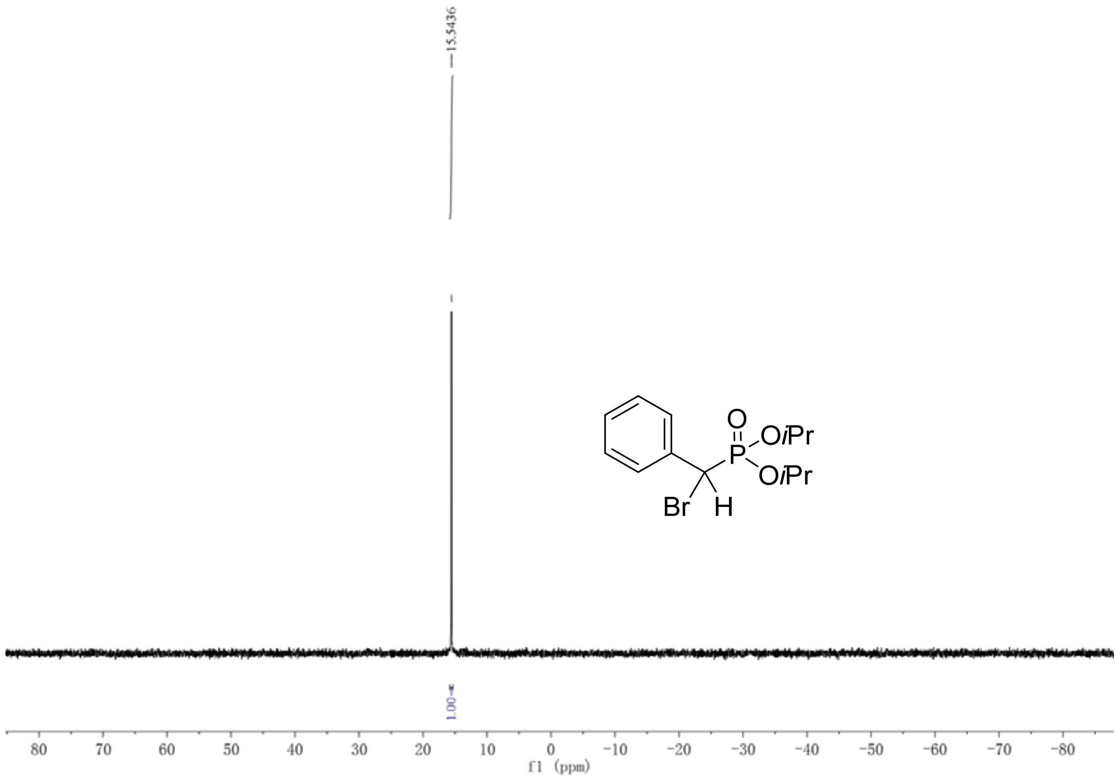

^{1}H NMR (400 MHz, CDCl3) of **3t**:

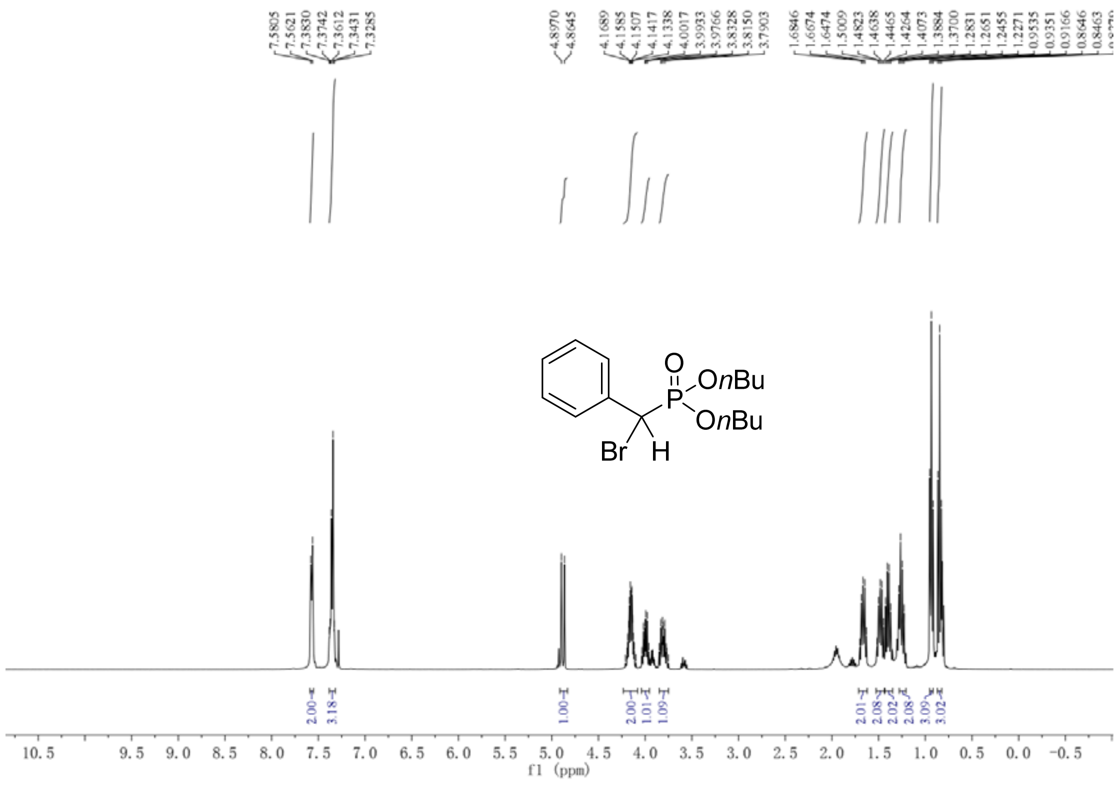

^{13}C NMR (100 MHz, $CDCl_3$) of **3t**:

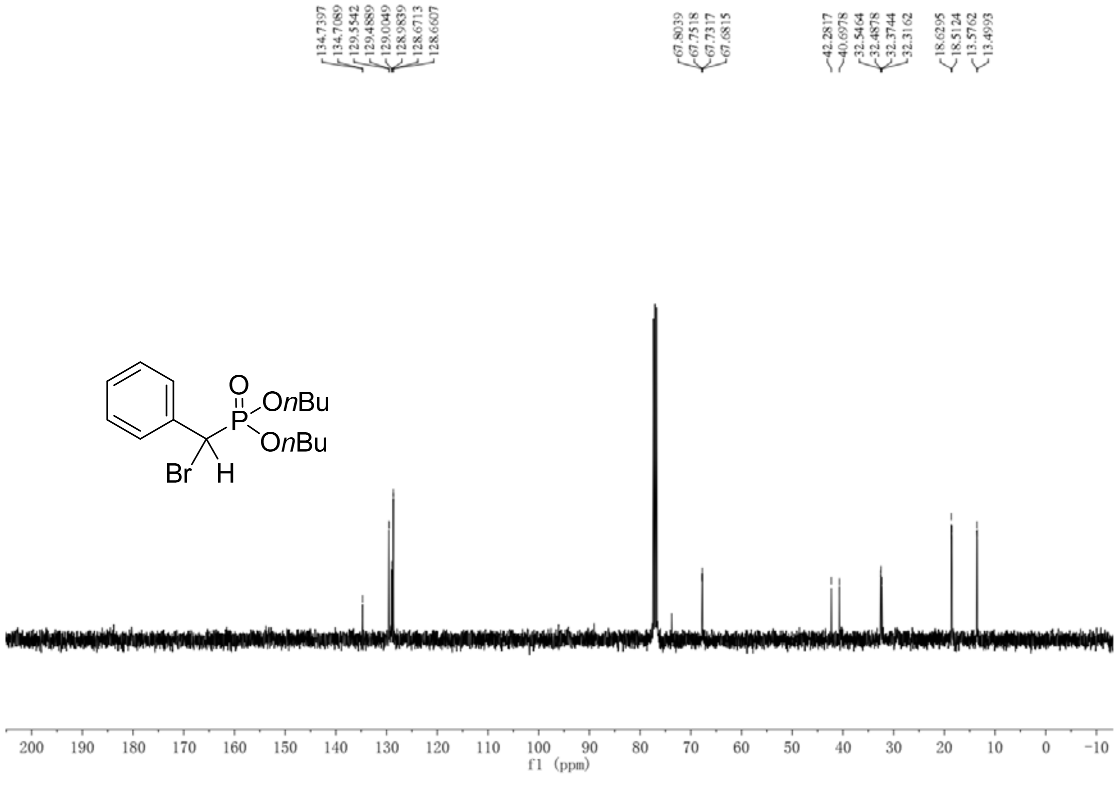

^{31}P NMR (162 MHz, $CDCl_3$) of **3t**:

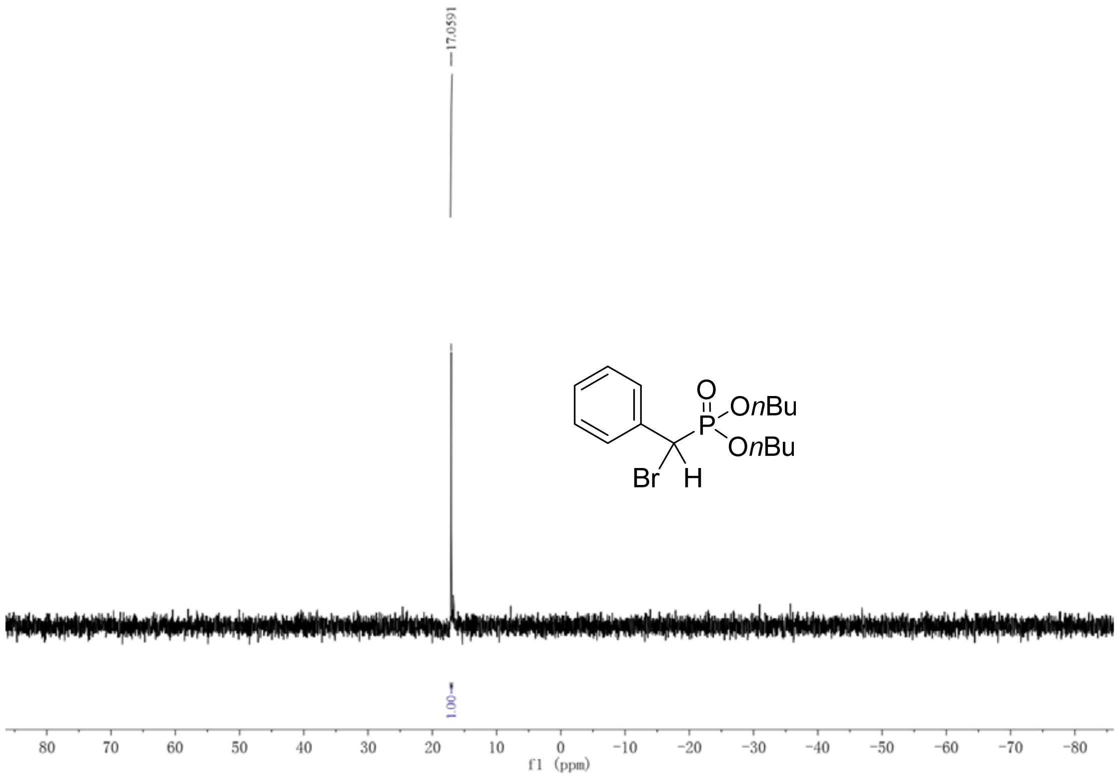

^{1}H NMR (400 MHz, CDCl3) of **5**:

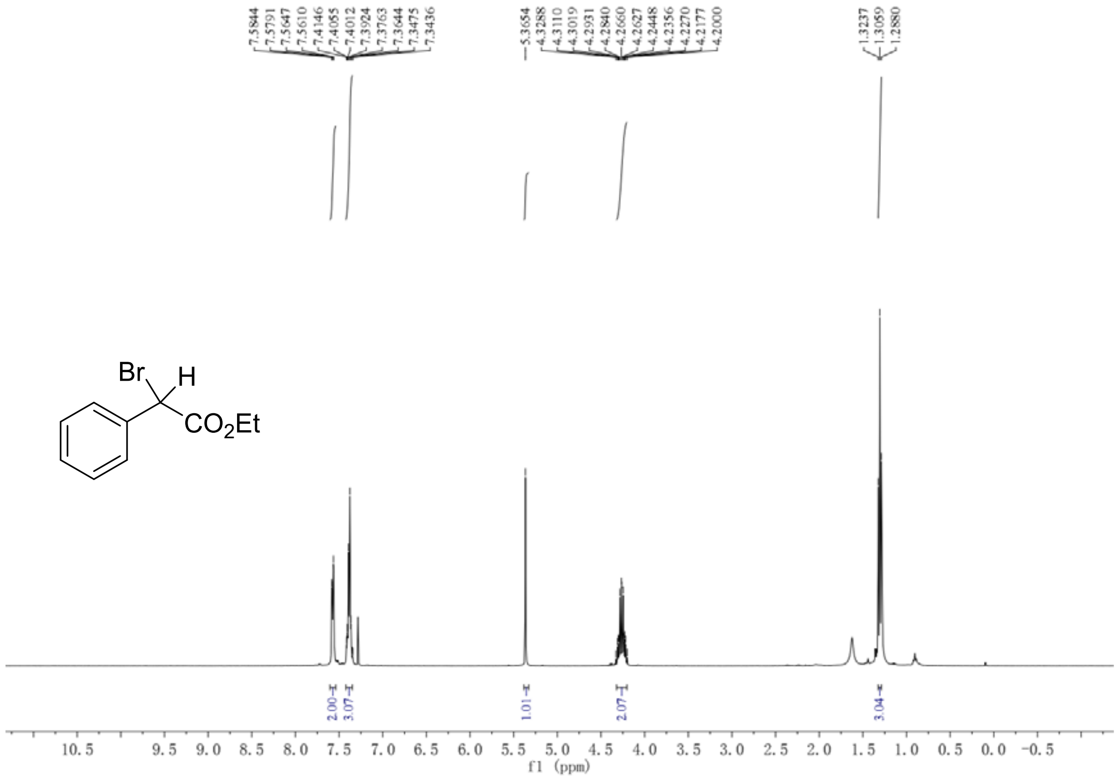

^{13}C NMR (100 MHz, $CDCl_3$) of **5**:

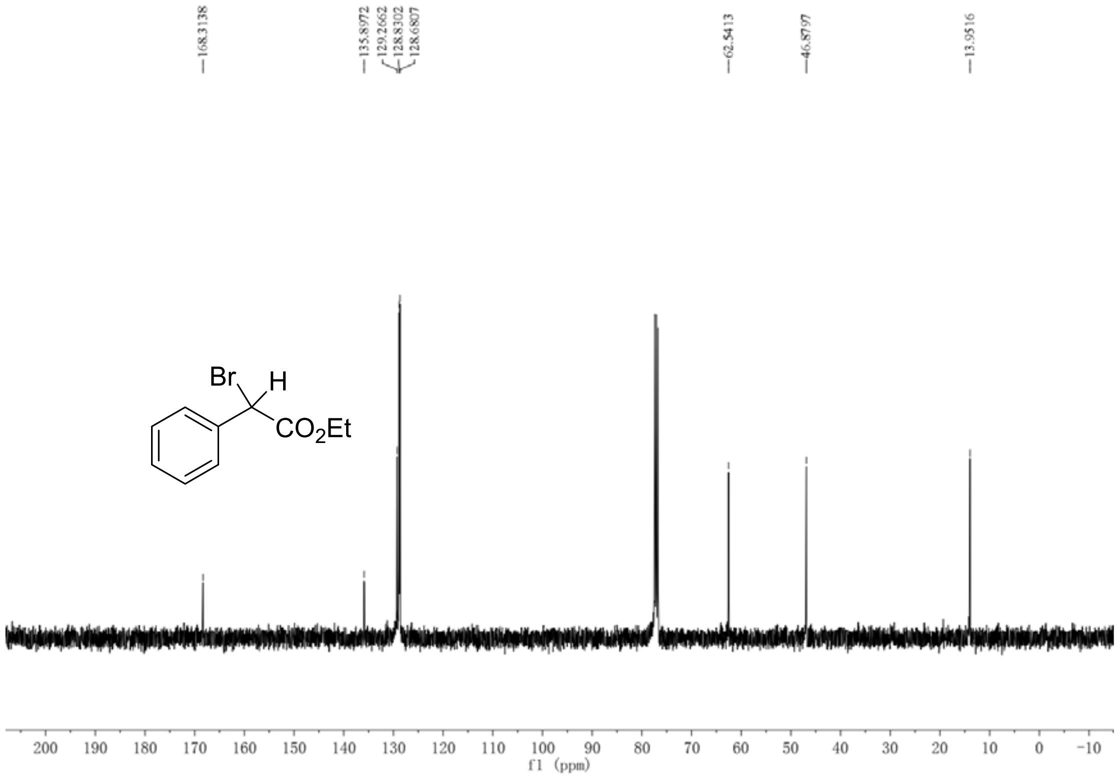

^{1}H NMR (400 MHz, CDCl3) of **6**:

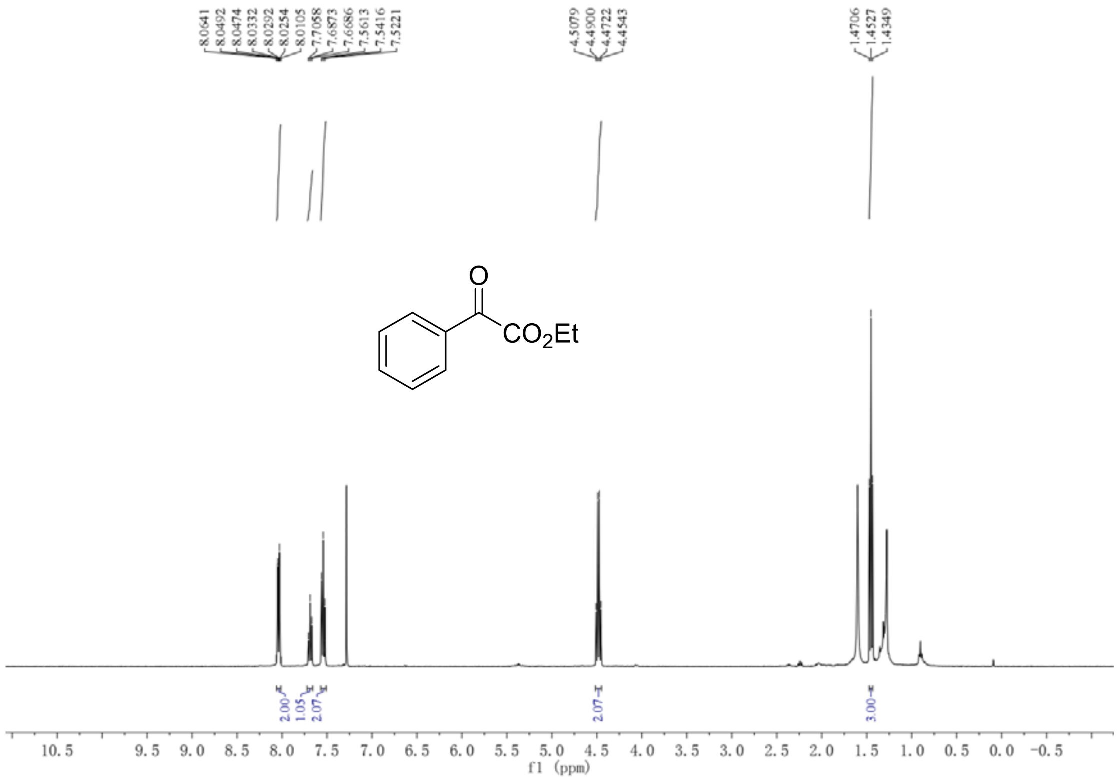

^{13}C NMR (100 MHz, $CDCl_3$) of **6**:

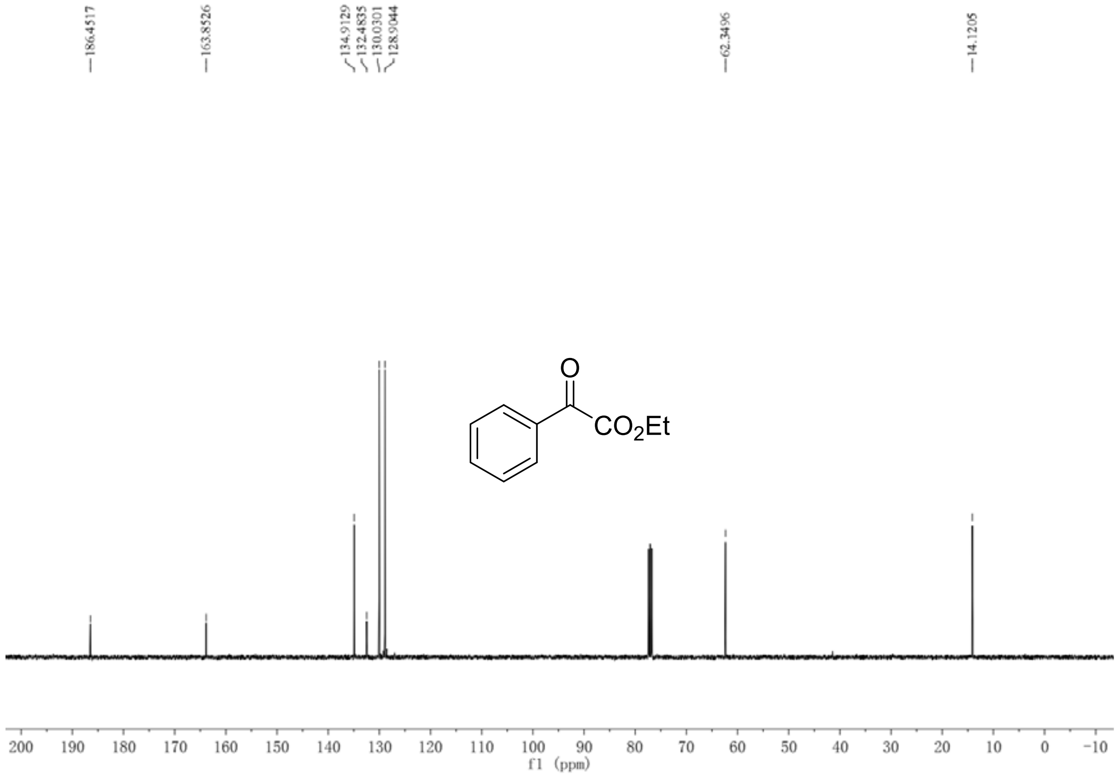

^{1}H NMR (400 MHz, CDCl3) of **7**:

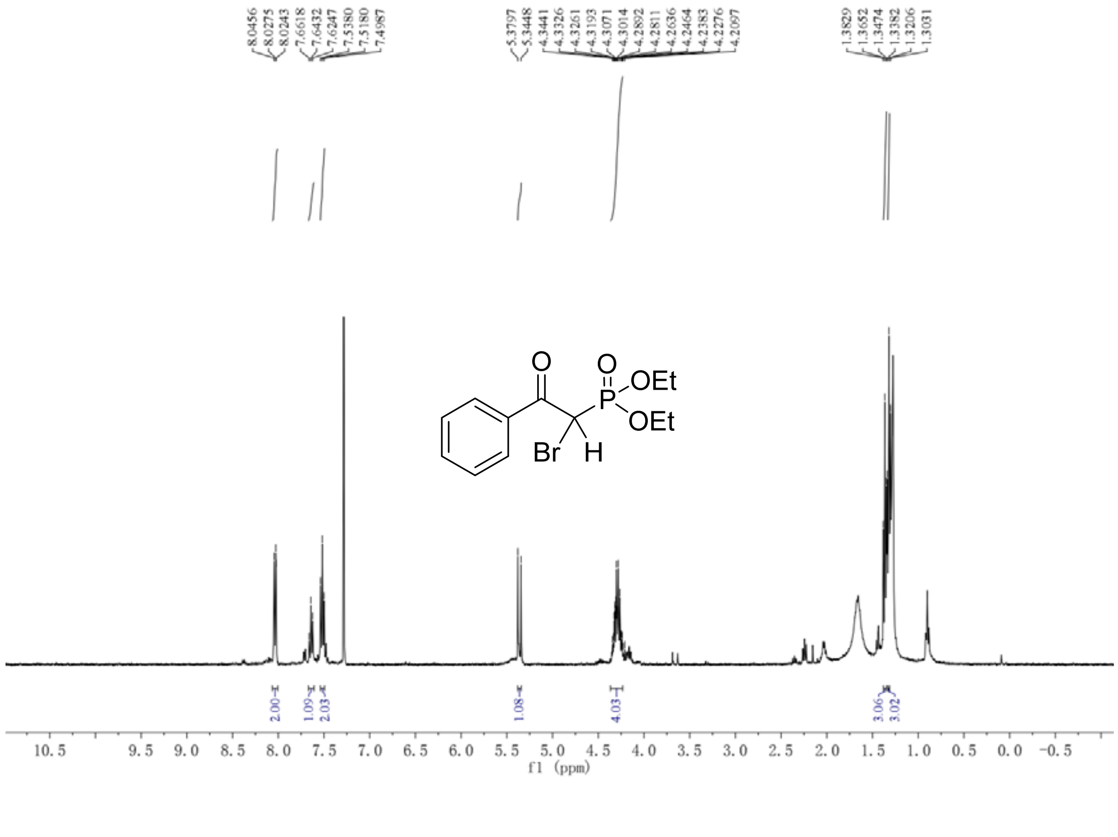

^{13}C NMR (100 MHz, $CDCl_3$) of **7**:

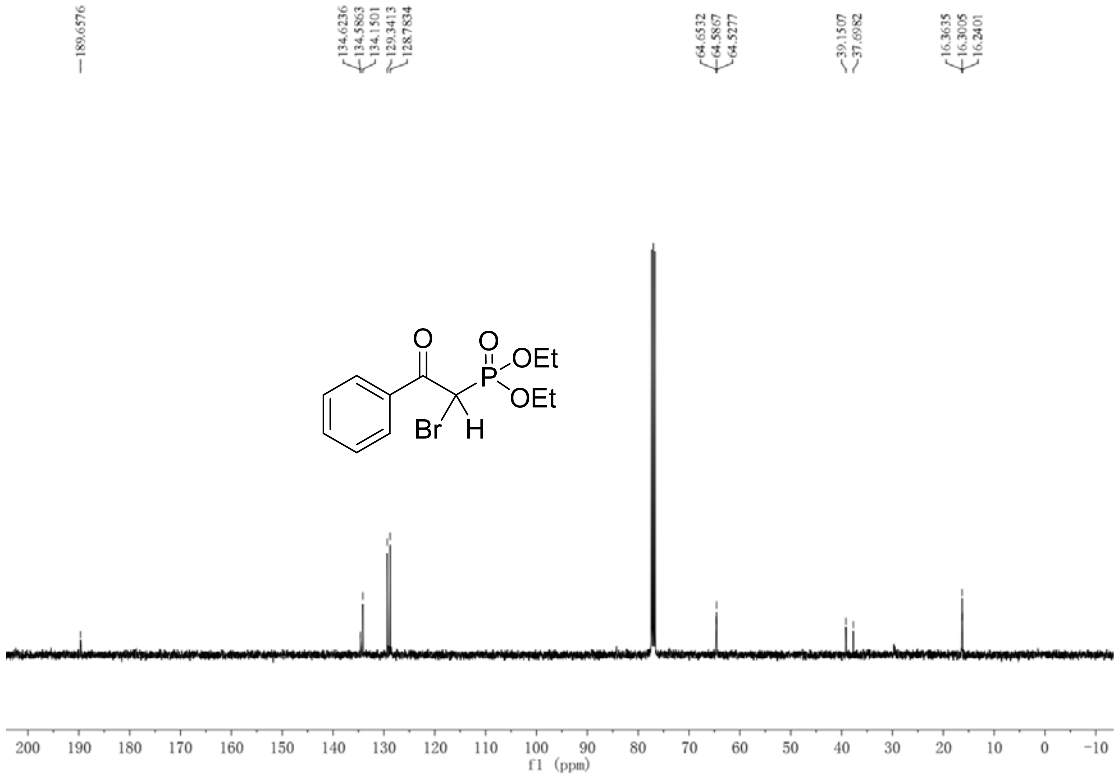

^{31}P NMR (162 MHz, $CDCl_3$) of **7**:

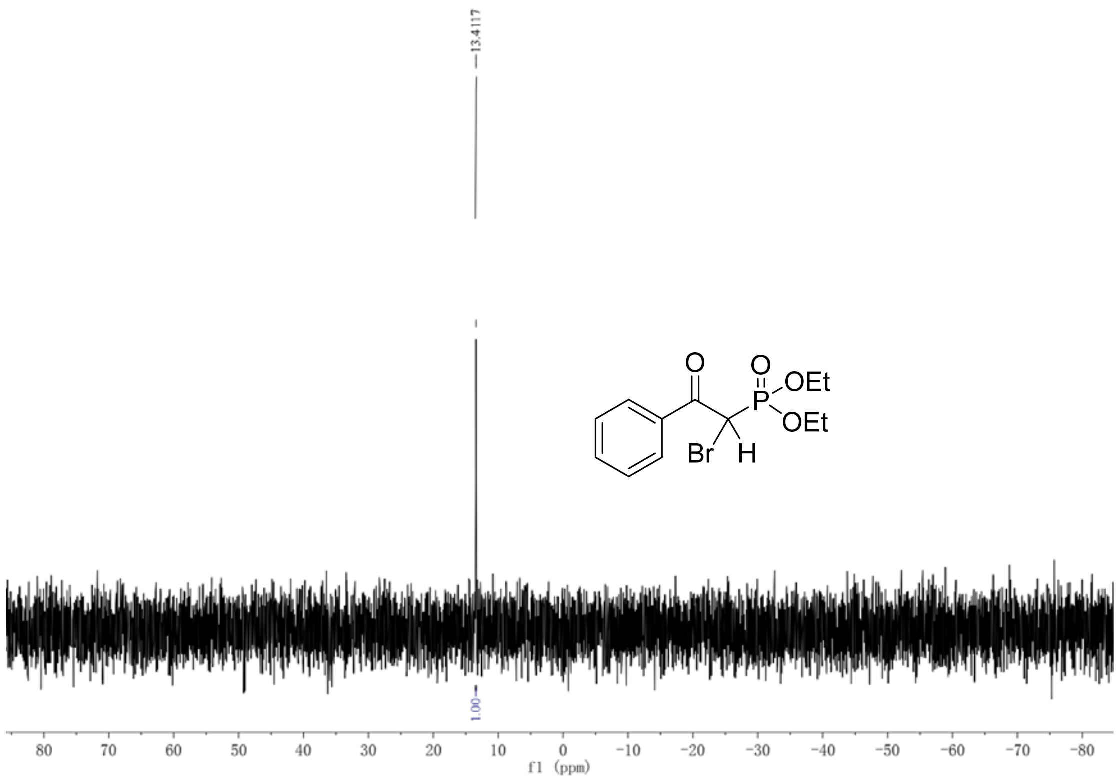

^1H NMR (400 MHz, CDCl3) of **8**:

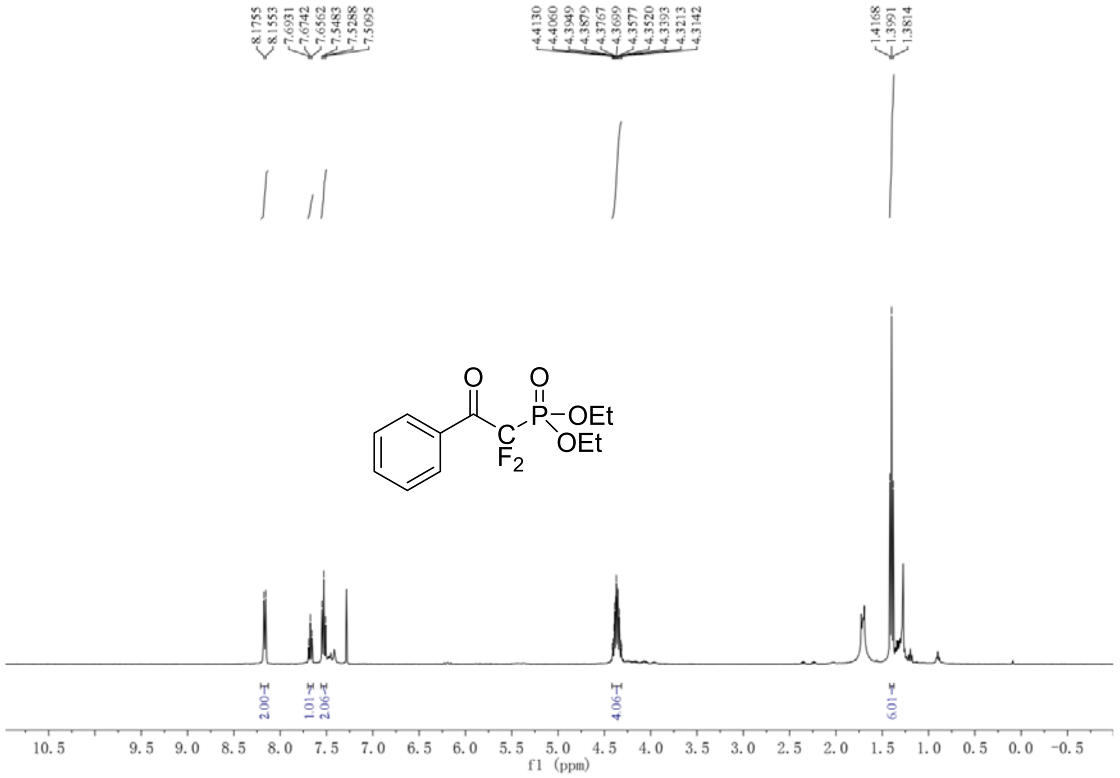

^{13}C NMR (100 MHz, $CDCl_3$) of **8**:

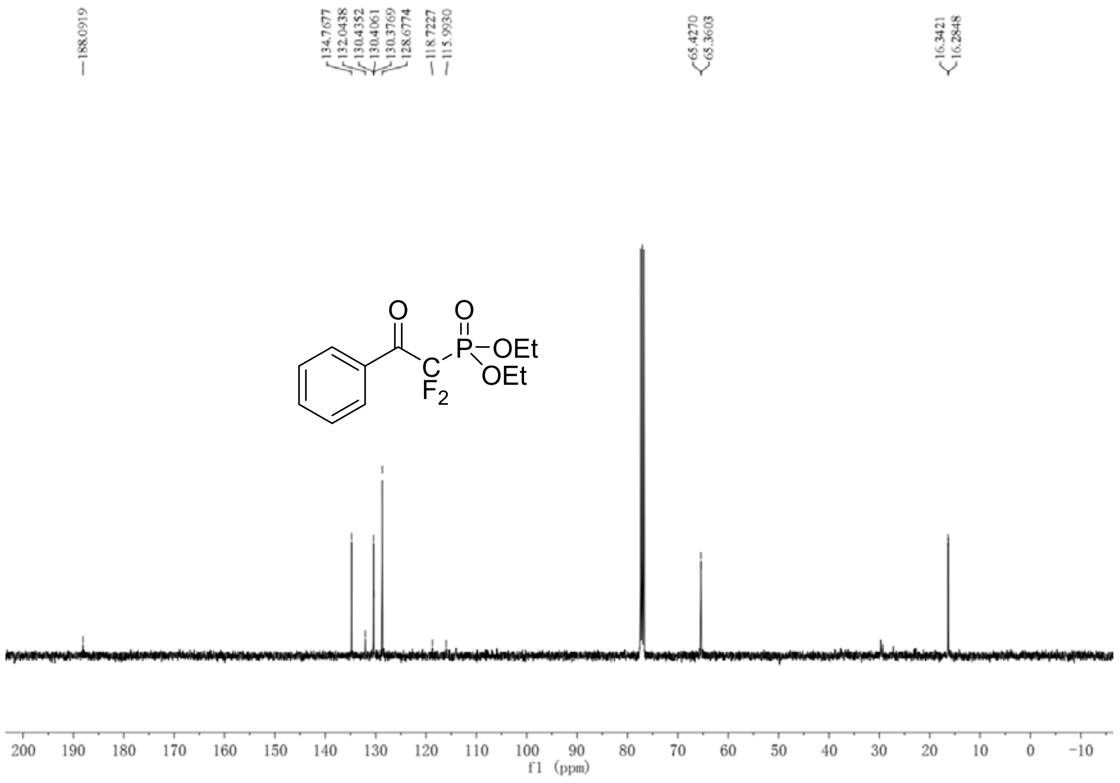

^{31}P NMR (162 MHz, $CDCl_3$) of **8**:

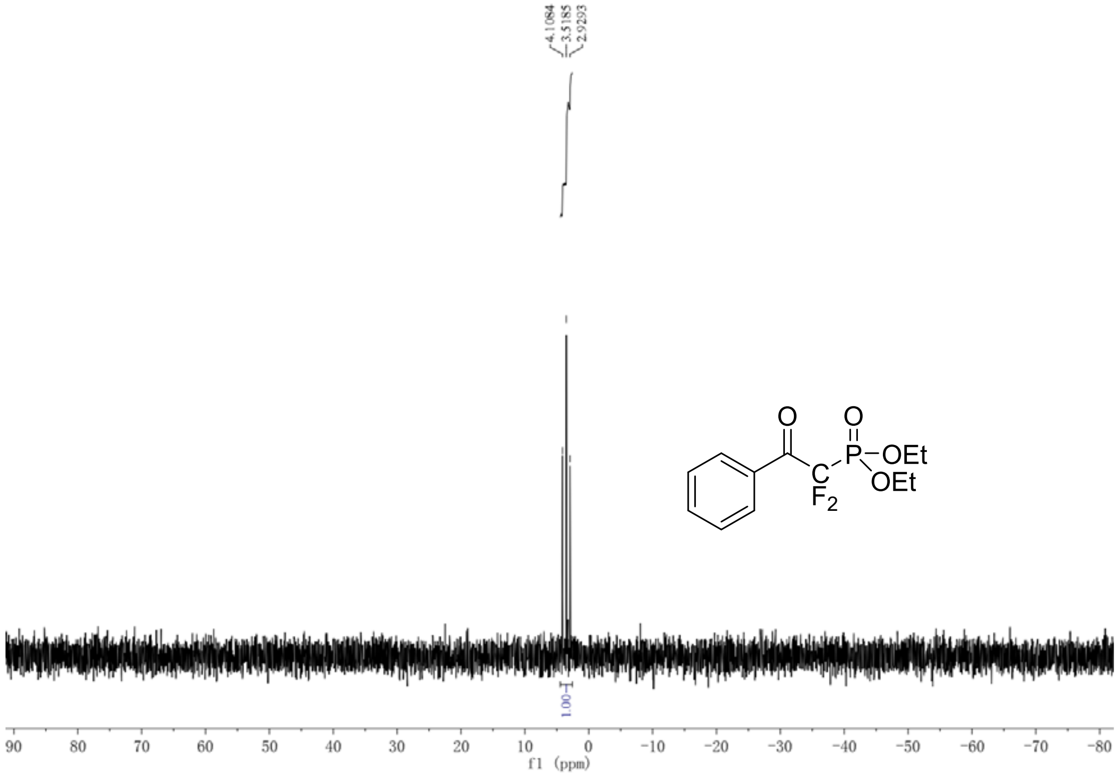

^{19}F NMR (376 MHz, $CDCl_3$) of **8**:

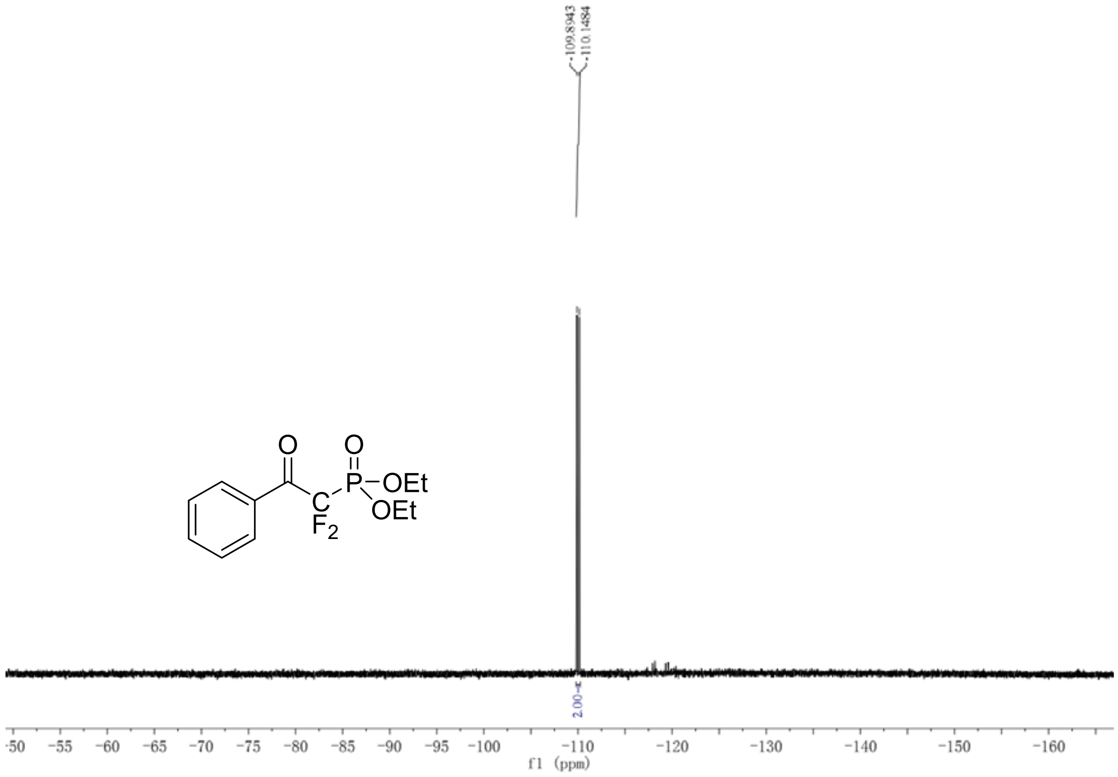

Supplement: Supplementary file 1 [file ol5c05051_si_001.pdf]
